# Zirconium-mediated carbon–fluorine bond functionalisation through cyclohexyne “umpolung”†‡

**DOI:** 10.1039/d4sc08522a

**Published:** 2025-01-16

**Authors:** Sara Bonfante, Theo F. N. Tanner, Christian Lorber, Jason M. Lynam, Antoine Simonneau, John M. Slattery

**Affiliations:** a LCC-CNRS, Université de Toulouse, CNRS UPS205route de Narbonne BP44099 F-31077 Toulouse cedex 4 France; b Department of Chemistry, University of York Heslington York YO10 5DD UK

## Abstract

Polarity reversal, or “umpolung”, is a widely acknowledged strategy to allow organic functional groups amenable to react in alternative ways to the usual preference set by their electronic features. In this article, we demonstrate that cyclohexyne umpolung, realized through complexation to zirconocene, makes the small strained cycloalkyne amenable to C–F bond functionalisation. Such strong bond activation chemistry is unprecedented in “free” aryne and strained alkyne chemistry. Our study also reveals that the reactivity of the Zr–cyclohexyne complex is highly sensitive to the degree of fluorination of the heteroarene. In addition, parasitic reactions of the ancillary ligand PMe_3_ were observed when pentafluoropyridine was the substrate.

## Abstract 2

Polarity reversal of cyclohexyne through its coordination to a metal center makes it capable of C–F bond activation.

## Graphical abstract

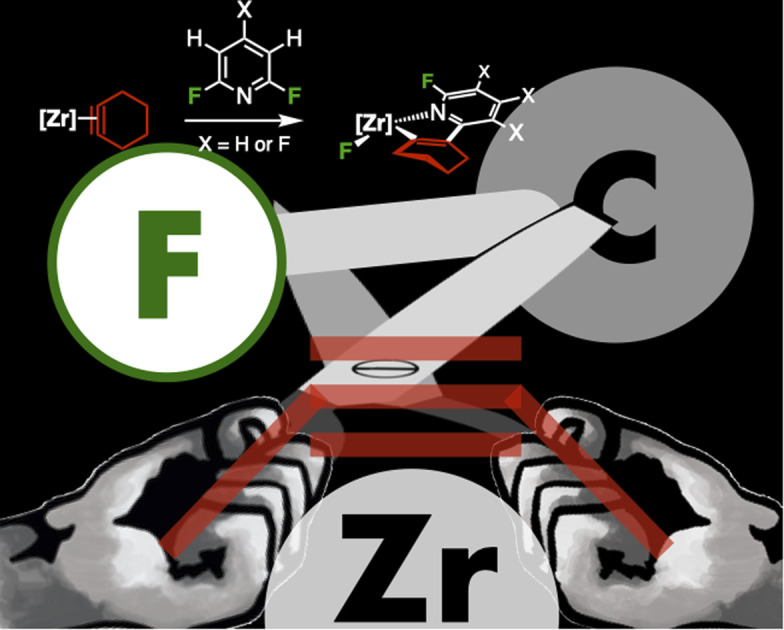

## Introduction

**Scheme 1:**
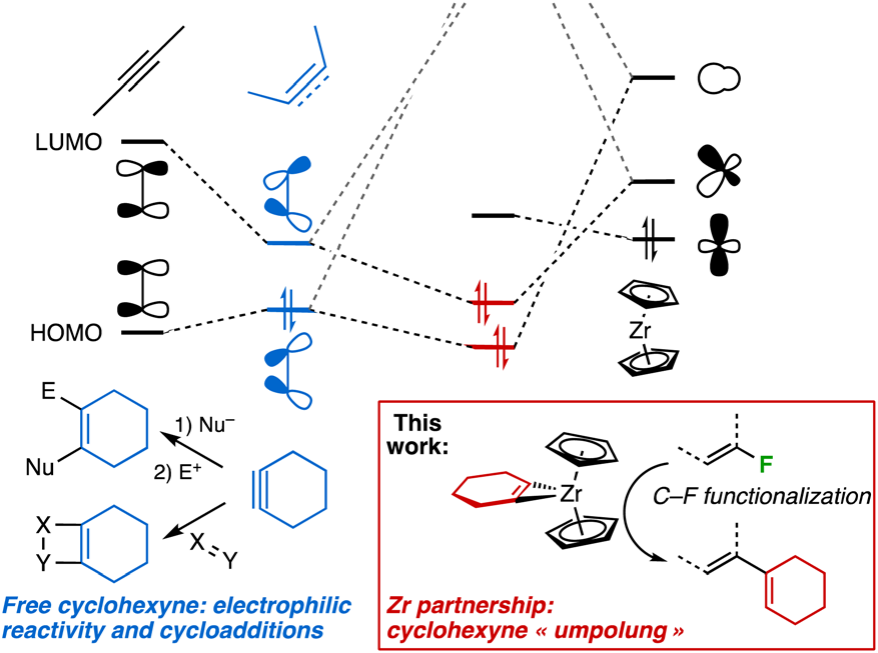
As a result of strong back-bonding to the LUMO of cyclohexyne, polarity reversal (“umpolung”) ensues from Zr complexation and makes it amenable to C–F bond activation.

Arynes and strained small cycloalkynes are fleeting intermediates that have experienced renewed interest over the past 15 years.^1^ Easier methods for their *in situ* generation have allowed the development of several useful synthetic protocols. In particular, cycloadditions or multicomponent reactions as well as formal insertions into polar X–Y bonds have been achieved, whereas their combination with late transition-metal-based methodologies have further expanded the portfolio of reactions achievable for these fascinating species.^1*e*,*g*^ Their intrinsic electrophilicity, driving their reactivity, is the consequence of poor overlap of the p-orbitals involved in the second π bond, resulting in significant lowering of the LUMO compared to linear alkynes.^2^ Arynes and strained alkynes can be stabilized by coordination to a low-valent transition metal center,^3^ from which a high degree of back-bonding may impart the strained fragment a nucleophilic behaviour (Scheme 1). This is a manifestation of the ability of transition metals to reverse the innate reactivity of organic ligands.^4^

Unlike transition-metal alkylidenes, alkylidynes or imides, which can promote the cleavage of C–H^5^ and C–F^5*c,f*,6^ bonds, there are only very limited examples in which aryne/strained alkyne complexes undergo bond activation and functionalisation processes on exogenous substrates.^7^ Conceptually, this would demonstrate that their “umpolung”^8^ may lead to bond activation processes that have remained elusive for “free” arynes and small cycloalkynes. We now report a successful application of this concept for C–F bond functionalisation with a metal-coordinated strained alkyne. Such a transformation is attractive given the ubiquity of C–F bonds in pharmaceuticals or agrochemicals,^9^ as a late-stage derivatization strategy, or to elaborate fluorinated synthons from simple and widely available per- or poly-fluorinated molecules.^10^

An important body of work exists concerning the functionalisation of polyfluoro-pyridines and -azines employing transition metals in either stoichiometric or catalytic amounts. Approaches based on the use of late transition metals^10*a*^ dominate this chemistry and generally rely on oxidative addition of the C–F bond followed by transmetalation and allow the introduction of aryl,^11^ vinyl,^12^ alkyl,^13^ acyl^14^ as well as silyl^12*b*^ or boryl^15^ groups. Although group 4 and 5 transition-metal complexes have been shown to be competent for C_Ar_–F bond activation^5*c*,*f*,6,16^ and their catalytic hydrodefluorination,^17^ there is in contrast only a handful of early transition metal (ETM) species reported to mediate C–F bond functionalisation of polyfluoropyridines. *In situ* generated imido^5*c*^ or η^2^-cyclopropene^16*i*^ zirconocene complexes as well as a titanium alkylidyne^5*f*,6^ react with C–F bonds of fluoropyridines to form C–N or C–C bonds through C–F-addition over the metal–ligand bond. The undeniable advantage of these methods is their perfect regioselectivity for the 2-position of the aryl compound, especially challenging when the 4-position also carries a fluorine atom.^16*i*,18,19^ Of note, the groups of Rosenthal and Beckhaus have explored the reactivity of group 4 metallocene–alkyne complexes with fluoropyridines, revealing the preference of the systems for 1,2-C–H addition or C–F bond oxidative addition, but no coupling of the alkyne with the fluorinated heterocycle was observed.^16*d*,*e*^ This work demonstrates that a cyclohexyne–zirconocene complex is effectively capable of C–F bond functionalisation of fluoropyridines. This is achievable thanks to the ring strain of the small cyclic alkyne ligand as well as its “umpolung” in the vicinity or zirconium, and results in net alkenylation of the heterocycle.

## Results and discussion

**Scheme 2:**
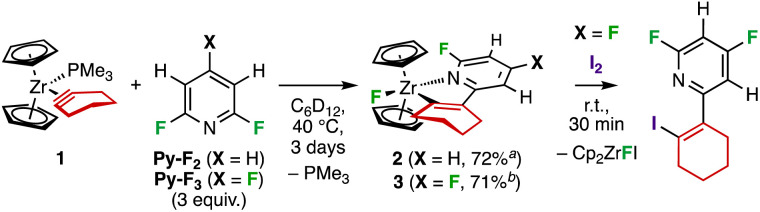
Reaction of 1 with Py-F*_n_* (*n* = 2, 3) leads to C–F alkenylation. ^*a*^NMR yield. ^*b*^Isolated yield.

The Zr complex [Cp_2_Zr(PMe_3_)(*c*-C_2_(CH_2_)_4_)] 1 (Scheme 2) was first prepared by Buchwald and co-workers. It was selected for this study as it reacts with various unsaturated organic molecules by insertion into the Zr–C bond.^20^ We commenced our study by treating 1 with 3 equiv. of 2,6-difluoropyridine (Py-F_2_) or 2,4,6-trifluoropyridine (Py-F_3_) in C_6_D_6_. In both cases, the targeted C–F functionalisation took place, unlike the previously reported reactivity of group 4 metallocene–alkyne complexes with polyfluoropyridines that leads to C–F metalation.^16*d*,*e*^ We thus obtained compounds [Cp_2_ZrF(κ^2^*C*,*N*–C_11_H_11−*n*_NF_1+*n*_)] (*n* = 0 or 1) 2 and 3, respectively (Scheme 2), which were identified on the basis of their ^1^H and ^19^F NMR spectra. Liberation of PMe_3_ was evidenced by its resonance at −62.0 ppm in the ^31^P NMR spectrum. The addition product 3 was characterised by a singlet at *δ* (^19^F) = −35.4 ppm, corresponding to the zirconium fluoride, in the ^19^F NMR spectrum. Moreover, a doublet at *δ* (^19^F) = −60.7 ppm (^4^*J*_FF_ = 24.0 Hz) and a doublet of doublets of doublets at *δ* (^19^F) = −94.8 ppm (^4^*J*_FF_ = 24.0, ^3^*J*_FH_ = 9.5 Hz, ^3^*J*_FH_ = 7.5 Hz) were associated with the *ortho*- and *para*-fluorine of the pyridine ligand, respectively. Both 2 and 3 are highly soluble and sensitive compounds, making their purification and isolation from the reaction mixture difficult. However, an optimal isolated yield of 71% for 3 was obtained when the reaction was carried out in cyclohexane instead of benzene.

After treatment of the reaction mixture containing 3 with I_2_, gas chromatography-mass spectrometry (GC-MS) analysis resulted in the detection of 2-(2-iodocyclohexen-1-yl)-4,6-difluoropyridine. This confirmed that products 2 and 3 were the result of 1,3-addition of the C–F bond over the metal–cyclohexyne linkage (Scheme 2). To the restricted portfolio of Zr-mediated C–F bond functionalizations, allowing introduction of H,^16*c*,17^ alkyl,^16*i*^ aryl^16*a*^ or amino^5*c*^ groups, this reaction adds an efficient and regioselective way to insert an alkene moiety. The strain imparted to the alkyne is instrumental in this outcome since zirconocene complexes of unstrained alkynes preferentially lead to oxidative addition of aromatic C–F bonds,^16*d*^ underpinning the special reactivity of the cyclohexyne ligand.

**Fig. 1:**
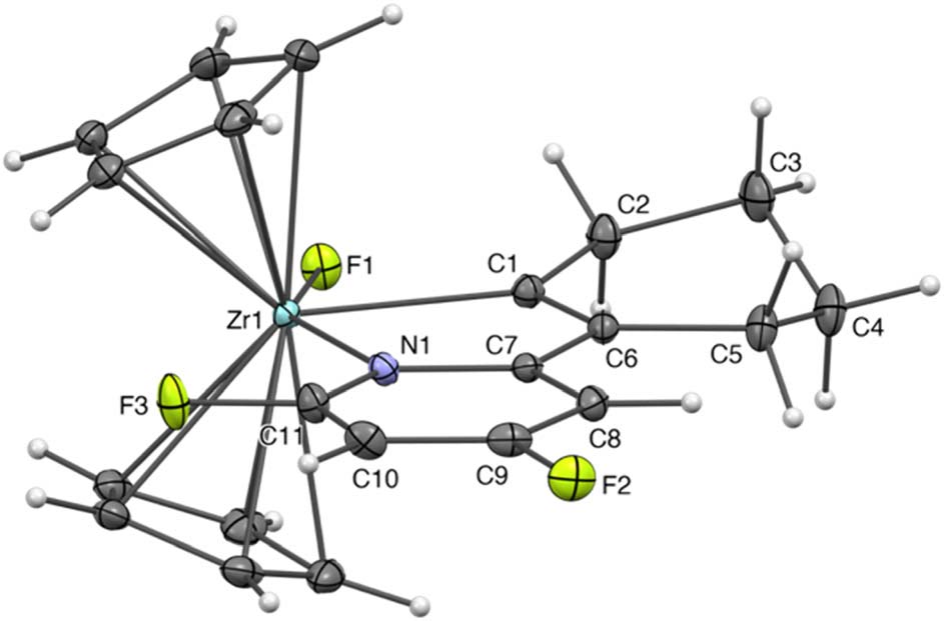
Molecular structure of 3 in the solid state. Ellipsoids drawn at the 25% probability level. Selected bond lengths (Å) and angles (°): Zr1–F1 2.008(1), Zr1–C1 2.321(2), Zr1–N1 2.479(2), N1–C7 1.372(3), C1–C6 1.354(3), C6–C7 1.456(3), F1–Zr1–C1 80.26(6), C1–Zr1–N1 68.35(6), F1–Zr1–N1 148.46(6), N1–C11–F3 114.7(2).

The molecular structure of 3 was determined by single crystal X-ray diffraction (Fig. 1) and provided definitive evidence for a C–F bond functionalisation at the *ortho* position of the fluoropyridine. The Zr atom lies in a distorted trigonal bipyramid environment, with apical sites occupied by the nitrogen of the pyridine ligand and the fluoride. The former belongs to a 1-zircona-2-aza-cyclopentadiene motif that connects it to the alkenyl carbon found in the equatorial plan, forcing the F–Zr–N angle to deviate significantly from linearity [F1–Zr1–N1 148.46(6)°] and imposing a tilt angle for the Cp–Zr–Cp moiety of 135.8°. Similar deviations from the ideal trigonal bipyramid geometry are found in other crystallographically characterized five-coordinated, Cp-supported 1-zircona-2-aza-cyclopentadienes.^21^ Compared to structurally related species (Table S3‡),^21,22^ the angles measured within the five-membered ring as well as the bond lengths of the conjugated part show no significant irregularity. However, the Zr–C bond is rather short and the Zr–N one, rather long. In the family of compounds selected for comparison, the former varies slightly in length, while the Zr–N distance fluctuates depending on the L or X character of the nitrogen ligand and the N-substituent. In the present case, we explain the long Zr–N bond by the presence of the fluoride ligand in a mutually *trans* position, coupled to the electron-poor properties of the difluoropyridine ring. The elongation of the Zr–N bond mechanically impacts the length of the Zr–C one due to the rigidity of the 1-zircona-2-aza-cyclopentadiene core. An additional observation is that the N1–C11–F3 angle is quite acute [114.7(2)°], presumably because of a long-range interaction of F3 with the electropositive Zr atom (Zr1–F3 3.469(2) Å < Σ*r*_W_).

**Scheme 3:**
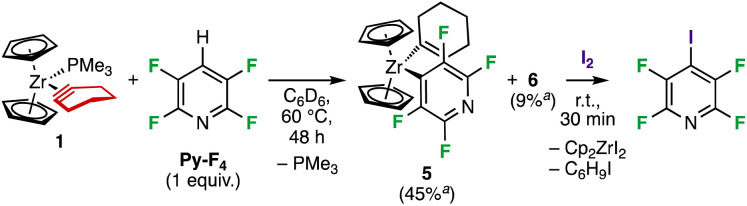
Reaction of 1 with Py-F_4_ leads to C–H activation. ^*a*^NMR yields.

We next turned our attention towards the reaction between 1 and 2,3,5,6-tetrafluoropyridine (Py-F_4_). When 1 was reacted with an equimolar amount of Py-F_4_ at 60 °C in C_6_D_6_, the main product was found to be the C–H activation product [Cp_2_Zr(1-C_6_H_9_)(4-C_5_F_4_N)] 5 (Scheme 3), similar to what was observed in the reaction of the Cp_2_Zr(η^2^-Me_3_SiC_2_SiMe_3_) complex with Py-F_4_.^16*d*^ This species was characterised by two multiplets in the ^19^F NMR spectrum at *δ* = −97.7 and −114.8 ppm associated with the *ortho*- and *meta*-F of the pyridine ligand, respectively. Small amounts of 1-cyclohexenylzirconocene fluoride (6, 9% after 48 h) also formed according to NMR analysis, that were found to increase with time as 5 decreased (NMR yield of 5 was 53% after 27 h but dropped to 45% after 48 h). We believe 5 arises from 1,2 C–H addition over the Zr–(η^2^-C≡C) bond in 1, a hypothesis that was further supported by the absence of deuterium incorporation from C_6_D_6_ into the cyclohexenyl ligand. Further evidence for C–H activation was obtained from NMR analysis of the reaction mixture after treatment with I_2_, which indicated the presence of 4-iodo-2,3,5,6-tetrafluoropyridine. Of note, the reaction mixture became more complex, with a range of unidentified products, when excess Py-F_4_ was employed.

**Scheme 4:**
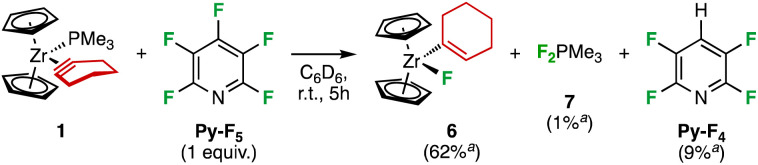
Species identified from the reaction of 1 with Py-F_5_. ^*a*^NMR yields.

Finally, we investigated the reactivity of 1 towards pentafluoropyridine (Py-F_5_). After four hours at room temperature in C_6_D_6_, 1 was fully converted into 1-cyclohexenylzirconocene fluoride ([Cp_2_ZrF(1-C_6_H_9_), 6, 62% NMR yield) (Scheme 4). An unidentified dark red solid that precipitated at the end of the reaction, amounting roughly 25–30% of the reactant mass and proving insoluble in usual organic solvents, including highly polar ones, as well as in water. The elemental analysis of two different samples showed the presence of nitrogen (*ca*. 3.5%), suggesting it may incorporate pyridinic moieties. While the carbon content was consistent (*ca*. 35%), the hydrogen varied from a sample to the other (2.7 to 4.9%). Inductively Coupled Plasma Optical Emission Spectroscopy (ICP-OES) revealed that the Zr and P contents fluctuate from 0.5–1.5% to 3–11%, respectively (Table S2‡).

These conflicting analyses point to an uncontrolled precipitation of this material. Infrared spectroscopy (ATR method) shows two common intense bands at 957–980 and 487–496 cm^−1^ within non-superimposable spectra (Fig. S12‡). The first band could be the result of P–F stretches that generally result in strong absorption in the 700–1000 cm^−1^ region for fluorophosphoranes and around 900 cm^−1^ for fluorophosphoniums.^23^ We also identified in the reaction mixture the hydrodefluorination product 2,3,5,6-tetrafluoropyridine (Py-F_4_, 9% NMR yield), trimethyldifluorophosphorane (F_2_PMe_3_, 7, 1% NMR yield) and a third product presumably resulting from the reaction of pentafluoropyridine with a phosphorus species, the precise identification of which remained elusive. The formation of 6, Py-F_4_ and 7 was ascertained by multinuclear NMR spectroscopy. To further evidence the presence of 6, 1 equivalent of I_2_ was added into the reaction mixture and the formation of 1-iodocyclohexene, Cp_2_ZrFI, Cp_2_ZrF_2_ and Cp_2_ZrI_2_ was observed by ^1^H NMR analysis. Moreover, the addition of hydrochloric acid to the crude mixture led to the liberation of cyclohexene (see ESI‡ for detailed NMR analysis). Different reaction conditions were investigated: changing solvent, concentration, reagent ratio and lowering of the temperature to 0 °C had only little influence on the reaction outcome. At −40 °C, the system was unreactive.

**Scheme 5:**
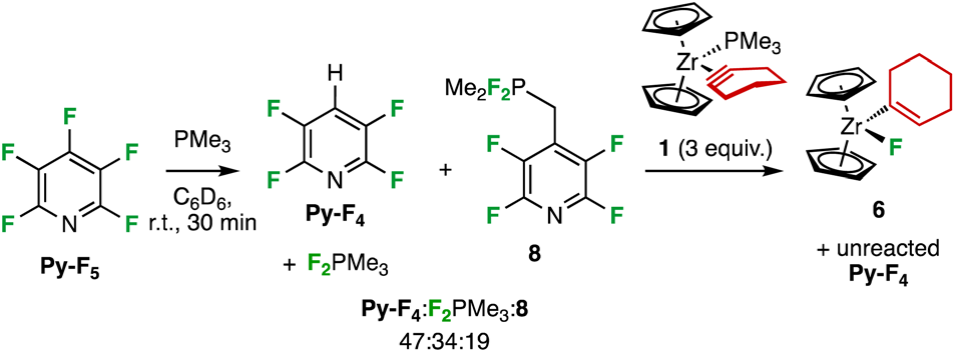
The reaction of PMe_3_ with Py-F_5_ affords Py-F_4_ and difluorophosphoranes F_2_PMe_3_ and 8. The difluorophosphoranes react with 1 to give 6.

Even though the mechanism leading to 6 is still unclear, it is proposed that the vinylic proton in 6 comes from the phosphine, since when the solvent used for the reaction was deuterated, no deuterium incorporation was found. The presence of the formal hydrodefluorination product Py-F_4_ recalls recent works demonstrating the ability of pnictogen compounds to achieve this transformation either stoichiometrically or catalytically.^24^ In particular, our groups jointly reported that simple trialkylphosphines are able to catalytically hydrodefluorinate Py-F_5_ to Py-F_4_,^24*a*^ and mechanistic investigations revealed that the catalytic cycle involves P(iii)/P(v) redox cycling and initiates with nucleophilic attack of the phosphorus atom onto the electron-deficient heterocycle^24*a*,*b*,*f*,25^*via* a Meisenheimer-like transition state. It is thus likely that the ancillary ligand PMe_3_ plays a role in the formation of Py-F_4_. Indeed, PMe_3_ reacts rapidly in C_6_D_6_ at room temperature with one equivalent of Py-F_5_ to afford a mixture in which Py-F_4_ and difluorophosphoranes F_2_PMe_3_ and 8 were identified as the major products (47 : 34 : 19 ratio, respectively, >90% NMR yield according to ^19^F NMR integration, Scheme 5). 8 is likely the result of S_N_Ar of Py-F_5_*ortho*-fluoride by the fluorophosphorus ylide Me_2_FP=CH_2_ (ref. 26) for which the way and means of its *in situ* formation are yet to be elucidated. When this mixture was treated with one equivalent of 1, the latter was immediately converted to 6 rather selectively, along with some insoluble materials. Addition of two more equivalent of 1 were necessary to completely consume F_2_PMe_3_ and 8, leaving 6 and unreacted Py-F_4_ as the main identifiable products of the reaction (see ESI‡ for details). The significant P content and low Zr one in the red solid, as well as a strong absorption in the P–F region indicates it presumably stems from another uncontrolled parasitic reaction between PMe_3_ and Py-F_5_, but its absence in the case of the reaction thereof without 1 suggests Zr plays a role in its formation.

Several hypotheses may be given to explain the differences in reactivity of the four polyfluoropyridines towards 1. The absence of a formal 1,2-C–F addition product after treatment with Py-F_5_ can reasonably be attributed to the fast reaction of the latter with dissociated PMe_3_. The low Lewis basicity of Py-F_5_ may also inhibit binding to the phosphine-free cyclohexyne complex, which may be an important prerequisite for the C–F bond activation event. Interestingly, during the synthesis of 1, an unsaturated [Cp_2_Zr(η^2^-*c*-C_6_H_8_)] cyclohexyne complex is supposed to form, to which PMe_3_ is then complexed to furnish 1. Addition of Py-F_5_ to the reaction mixture before addition of PMe_3_ resulted in no reaction, suggesting the Zr centre is unable to coordinate Py-F_5_ and trigger C–F bond activation. Additionally, the structure of 3 (Fig. 1) reveals a 1,3-allylic (^1,3^A) strain between the cycles' backbones (carbons C5 to C8), along the ring junction. This destabilizing interaction would be exacerbated if the heteroaromatic ring was fully fluorinated and may exist before the C–C bond formation event, imparting a kinetic penalty for a C–F bond activation pathway. The observation of the C–H activation product in the reaction with Py-F_4_ may be the result of two factors: on the one hand, the high level of fluorination of the heterocycle makes it, akin to Py-F_5_, a poor Lewis base that is not likely to strongly bind the Zr center. Besides, a potential C–F/CH_2_ repulsion, resulting in the previously mentioned ^1,3^A strain in the cyclometalated product (Fig. 1), is likely to arise from Py-F_4_ coordination. On the other hand, the metalation of the acidic C–H bond provides a metal-4-pyridyl compound with highly stabilizing two-fold *ortho* fluorine substitution that considerably strengthen the Zr–C bond.^27^ Delineation of the factors that govern C–F *vs.* C–H bond activation in fluoroaromatics has been the object of several experimental and theoretical studies,^28^ revealing marked differences between metals. Focusing on Zr, our observation seems to be in line with the one of the Rosenthal group with [Cp_2_Zr(η^2^-Me_3_SiC≡CSiMe_3_)].^16*d*^ The latter shows preference for C–H over C–F bond activation when reacted with 2,3,5,6- or 2,4,5,6-tetrafluoropyridine. In the case of Py-F_3_ and Py-F_2_, higher Lewis basicity may allow the heteroarene to better interact with the Zr atom after PMe_3_ dissociation and thus bringing its *ortho* C–F bonds closer to the reactive cyclohexyne ligand—the stage is set for a regioselective C–F bond activation to take place.

**Fig. 2:**
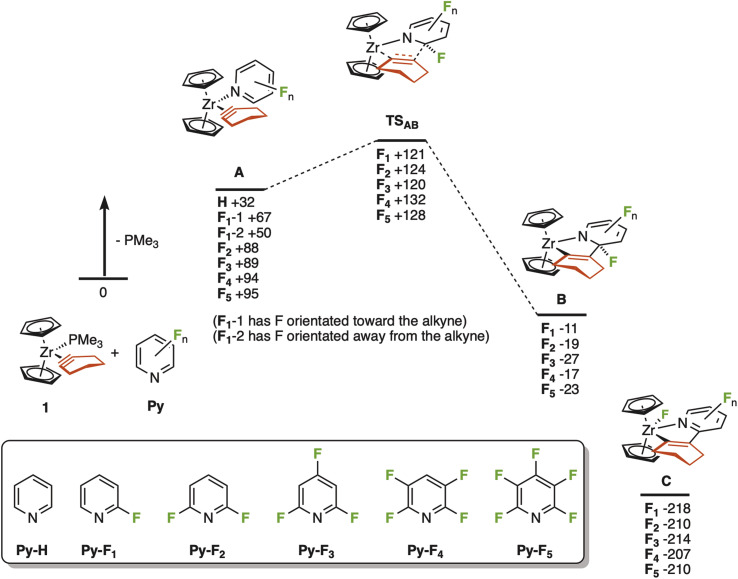
Potential energy surface for the reaction between 1 and substituted pyridines. Energies are Gibbs energies at 298 K in kJ mol^−1^ at the D3(BJ)-pbe0/def2-TZVPP//bp86/sv(p) level of theory with COSMO solvent correction in cyclohexane relative to 1 and the corresponding uncoordinated pyridine ligand. Insert shows the labelling code for the substituent patterns used.

A series of calculations using density functional theory (DFT) were undertaken to investigate this proposal. The interaction between the putative 16-electron complex [Cp_2_Zr(*c*-C_2_(CH_2_)_4_)] and a range of different fluorine-substituted pyridine ligands, as well as parent pyridine itself, was investigated. This was envisaged to give structure A (Fig. 2). The resulting calculations demonstrated that in all cases PMe_3_ binds more strongly to the zirconium than all the pyridine ligands investigated, in the order PMe_3_ ≫ Py-H > Py-F_1_ > Py-F_2_ ∼ Py-F_3_ ∼ Py-F_4_ ∼ Py-F_5_. In the case of A-F_1_, two isomers may be considered, with the fluorine orientated either towards or away from the cyclohexyne ligand. The former (A-F_1_-1) is 17 kJ mol^−1^ higher in energy than the latter (A-F_1_-2) which may represent a repulsive interaction between the fluorine lone pairs and the cyclohexyne ligand. Such an interaction could be playing a role in the polyfluorinated analogues too. An examination of the bond metrics within the optimised structures supported the arguments that the more fluorinated ligands might be expected to show weaker Zr⋯N interactions. As shown in Table S4,‡ there is notable lengthening of this distance with an increasing degree of fluorination.

A transition state corresponding to C–C bond formation between the cyclohexyne and pyridine was located (TS_AB_, Fig. 2a) for all the fluorinated analogues. There was little overall effect of the degree of fluorination on the barrier to this step with respect to 1 and the free pyridines, but it should be noted that when compared to complex A, the barrier was lower in the cases of F_2_–F_5_ (31–38 kJ mol^−1^) than F_1_ (54 kJ mol^−1^). This transition state connects A with a Zr(iv) complex, B, which is probably best viewed as containing a bidentate ligand with coordinating alkenyl and amido groups, the pyridine ligand having been dearomatized.^6*c*,16*i*^

An examination of the calculated bond metrics in the complexes A (Table S4‡) supports the notation that this C–C bond formation is best considered as a case of “umpolung” reactivity. The calculated alkyne C–C bond lengths range from 1.334–1.377 Å, much closer to the corresponding C=C bond length for cyclohexene (1.351 Å) than the C≡C length in cyclohexyne (1.237 Å) calculated at the same level of theory—it is worth noting that the cyclohexyne C–C bond in complex 1 has been measured at 1.295(25) Å,^20^ lying in between these two extrema. Indeed, in the case of the putative 16-electron complex [Cp_2_Zr(*c*-C_2_(CH_2_)_4_)] the C–C bond is 1.356 Å. These data indicate that there is extensive π-backbonding between the Zr and cyclohexyne in this system and is modulated by the electron-richness of the ligand. In the pyridine series, it is probably best considered as a Zr(iv) complex with a coordinated metallacyclopropene. Transition state TS_AB_ can therefore be viewed as an intramolecular organometallic nucleophilic attack onto the pyridine, which is templated by a weak Zr⋯N interaction, consistent with an “umpolung” view of alkyne reactivity.

In all cases, the experimentally observed product arising from the reaction between Py-F_2_ and Py-F_3_ (experimental complexes 2 and 3, corresponding to states C-F_2_ and C-F_3_) was significantly thermodynamically favourable, presumably representing the formation of a strong Zr–F bond.

The results from this computational study therefore demonstrate that a plausible pathway for the intramolecular “umpolung” reaction is viable in all cases. This provides some evidence to support the argument that in the cases of Py-F_4_ and Py-F_5_, the predominance of competing reactions overrides the C–F functionalisation pathway, rather than any intrinsic barrier.

Other polyfluoroarenes were evaluated for C–F bond functionalisation using 1: hexafluorobenzene (C_6_F_6_) proved unreactive, in line with N → Zr interaction being a pre-requisite for the C–F bond activation to occur. The reaction between 1 and 2-fluoropyridine (Py-F_1_) led to full consumption of the former and produced a mixture of Zr fluorides as attested by ^19^F NMR. Our attempts with 2,4-difluoropyridine (Py-F_2_′) were not reproducible, but led in any case to intractable mixtures of fluorinated and organometallic compounds. ^19^F NMR monitoring of these reaction did reveal 6 formed in significant amounts though.

## Conclusions

This work demonstrates for the first time that polarity reversal, or “umpolung”, of a small cycloalkyne is a reliable strategy to make fleeting strained intermediates amenable to strong bond activation processes they are normally not competent for. Thus, a metal–cycloalkyne complex can engage in C–F bond functionalisation of fluoropyridines, contrasting with the chemistry of acyclic alkyne complexes of group 4 metallocenes.^16*d*,*e*^ We are convinced that this reactivity should be reachable for other metal–aryne or -cycloalkyne complexes,^3^ such as Ni or Pd ones, showing reactivity that is reminiscent of strained alkyne “umpolung”.^4*b*–*e*^ The degree of fluorination of the substrate drastically changes the outcome; kinetic and thermodynamic reasons may be invoked to explain why the desired C–F functionalisation was not observed for Py-F_4_ and Py-F_5_. Our results may extend to Zr–aryne complexes giving a likely direction for our future work.

## Data availability

Raw NMR and IR data are freely available from the Recherche Data Gouv database (https://doi.org/10.57745/VGHSZN). Results of data treatment (NMR, crystallographic details, DFT) as well as atomic coordinates for computed species have been compiled in a .pdf file freely available as part of the ESI.‡ X-ray diffraction data have been deposited on the Cambridge Crystallographic Data Centre (CCDC) under the deposition number 2339708.

## Author contributions

S. B. ran the experiments, analysed the data and compiled the ESI. J. M. L. performed DFT calculations, analysed the theoretical results and wrote the computational part of the manuscript. T. F. N. T. recorded single-crystal X-ray diffraction data and solved the structures. C. L., J. M. L., A. S. and J. M. S. obtained funding for the project, managed it and took part in the analysis of the data. A. S. wrote the first draft of the manuscript. The manuscript and ESI were then further revised through contributions of all authors.

## Conflicts of interest

The authors declare no conflicts of interest.

## Supplementary Material

SC-OLF-D4SC08522A-s001

SC-OLF-D4SC08522A-s002
