# Supplementary material for: Zirconium-mediated carbon–fluorine bond functionalisation through cyclohexyne “umpolung”
Source: Chem Sci. 2025 Jan 16;16(8):3552–9. doi: 10.1039/d4sc08522a (PMC11758233; doi:10.1039/d4sc08522a)
Supplement: SC-016-D4SC08522A-s001 [file SC-016-D4SC08522A-s001.pdf]

Supplementary Information (SI) for Chemical Science.
This journal is © The Royal Society of Chemistry 2025

Zirconium-Mediated Carbon-Fluorine Bond Functionalization Through Cyclohexyne "Umpolung"

Sara Bonfante,[a,b] Theo F. N. Tanner,[b] Christian Lorber,[a*] Jason M. Lynam,[b*] Antoine Simonneau[a*] and John M. Slattery[b*]

[a] LCC-CNRS, Université de Toulouse, CNRS, UPS, 205 route de Narbonne, BP 44099, F-31077 Toulouse cedex 4, France.

[b] Department of Chemistry, University of York, Heslington, York YO10 5DD, UK.

Supporting Information

Contents

1 Experimental Details **3**

1.1 General methods *3*

1.2 Procedure for the synthesis of the 1-bromocyclohexene *4*

1.3 Procedure for the synthesis of the methylzirconocene chloride *5*

1.4 Procedure for the synthesis of complex 1 .... *6*

1.5 Procedure for the stability test of complex 1 .... *7*

1.6 Procedure for the synthesis of complex 3 .... *8*

1.7 Procedure for the synthesis of 2,4-difluoro-6-(2-iodocyclohexen-1-yl)-pyridine *11*

1.8 Procedure for the solvent optimisation in the synthesis of complex 3 .... *14*

1.9 Procedure for the synthesis of complex 2 .... *15*

1.10 Procedure for the synthesis of complex 5 and its iodinolysis *18*

1.11 Study of the reactivity of the zirconocene-cyclohexyne transient intermediate with Py-F_5 .... *23*

1.12 Procedure for the synthesis of 6 .... *24*

1.13 Procedure for the ligand liberation of complex 6 via HCl addition *26*

1.14 Procedure for the ligand liberation of complex 6 via I_2 addition *27*

1.15 Study on the solvent optimisation for the synthesis of complex 6 .... *28*

1.16 Study on the temperature optimisation for the synthesis of complex 6 .... *29*

1.17 Study on the concentration optimisation for the synthesis of complex 6 .... *30*

1.18 Study on the reagent ratio optimisation for the synthesis of complex 6 .... *30*

1.19 Procedure for the reactivity test of 1 with difluorophosphoranes 8 and F_2PMe_3 .... *31*

2 X-ray crystal structure **33**

2.1 Molecular structure of 3 .... *33*

2.2 Structural data comparison *34*

3 DFT calculations **36**

3.1 General Methods *36*

3.2 Collated Energies and xyz coordinates *37*

4 References **95**

1 Experimental Details

1.1 General methods

Unless otherwise indicated, all reactions were performed in flame- or oven-dried glassware with rigorous exclusion of air and moisture, using a nitrogen or argon-filled glove box (O_2 < 1 ppm, H_2O < 1 ppm) or regular Schlenk techniques.[1] Liquids were transferred using either plastic syringes, Teflon canulae with or without filtering tip, or Hamilton™ microsyringes. Anhydrous Et_2O, CH_2Cl_2, pentane and toluene were pre-dried by passing through a Puresolv MD 7 solvent purification machine. THF, deuterated-THF, deuterated toluene and C_6D_6 were dried over metallic sodium, purified by trap-to-trap transfer and degassed by three cycles of freeze-pump-thaw. CD_2Cl_2 and CD_3CN were dried over calcium hydride for three days, purified by trap-to-trap transfer and degassed by freeze-pump-thaw. Cyclohexane and deuterated cyclohexane were dried over molecular sieves prior to use. Reagents were purchased from commercial suppliers and purity was confirmed by NMR spectroscopy. The fluorinated aromatics were purchased from Sigma Aldrich, Fluorochem and Apollo Scientific and dried over molecular sieves prior to use.

All NMR spectra were obtained using J. S. Young's NMR tubes sealed under argon or nitrogen. ^1H, $^{13}C\{^1H\}$, ^{29}Si, ^{19}F and $^{31}P\{^1H\}$ NMR spectra were recorded on Bruker Avance II 400 MHz. Chemical shifts are in parts per million (ppm) downfield from tetramethylsilane and are referenced to the residual solvent resonances as the internal standard ($C_6\mathbf{H}D_5$: δ reported = 7.16 ppm; $C\mathbf{H}DCl_2$: δ reported = 5.32 ppm; $C_6H\mathbf{D}_{11}$ = 1.38 ppm; $C_4\mathbf{H}D_7O$ = 3.58 ppm; $C_7\mathbf{H}D_{12}$ = 2.09 ppm for ^1H NMR). $^{13}C\{^1H\}$ NMR spectra were calibrated according to the IUPAC recommendation using a unified chemical shift scale based on the proton resonance of tetramethylsilane as primary reference.[2,3] ^{19}F NMR chemical shift δ are reported in ppm, relative to the resonance shift of an external solvent $CFCl_3$, at δ 0.0 ppm. $^{31}P\{^1H\}$ and ^{31}P NMR chemical shifts reported in ppm, relative to the resonance shift of an external solvent H_3PO_4 at δ 0.0 ppm. Data are reported as follows: chemical shift, multiplicity (br = broad, s = singlet, d = doublet, t = triplet, q = quartet, p = quintet, sext = sextet, hept = heptet, m = multiplet, mc = multiplet center, tm = triplet of multiplets, dm = doublet of multiplets), coupling constant (Hz) and integration. ^1H and $^{13}C\{^1H\}$ resonance signals were attributed by means of 2D ^1H COSY, ^1H-^{13}C HSQC, ^1H-^{13}C HMBC, ^1H-^{31}P HMBC and ^1H-^{19}F HMBC experiments. The yields of compounds **1** and **3** are isolated yields. The NMR yields of compounds **2**, **5**, **6** were calculated from the ratio of the integrals of the signals of complex **1** and the deuterated solvent (C_6D_6 or C_6D_{12}) before the reaction (blank sample) and the ratio of the signals of the products and solvent at the end of the reaction.

Elemental analyses were performed in the facility available in Laboratoire de Chimie de Coordination (CNRS) using PerkinElmer 2400 Series Analyser. GC/MS analyses were performed with a Shimadzu QP2010 Ultra equipped with a ZB-5MS GC column (electronic impact ionizer and orbitrap analyzer). Mass spectral data are

quoted as the m/z ratio along with the relative peak height in brackets (base peak = 100). Mass to charge ratios (m/z) are reported in Daltons.

Single-crystals diffraction data were collected using a SuperNova diffractometer at 100 K using Cu Kα radiation (λ = 1.54184 Å) filtered through a graphite monochromator.

1.2 Procedure for the synthesis of the 1-bromocyclohexene

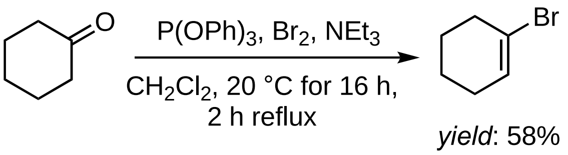

1-Bromocyclohexene was prepared according to a reported procedure.[4] To a cold solution of triphenyl phosphite (9.25 mL, 35.0 mmol, 1.1 equiv.) in anhydrous CH_2Cl_2 (100 mL) maintained at –60 °C under Ar flow, bromine (2.00 mL, 38.5 mmol, 1.2 equiv.) was added dropwise. Anhydrous triethylamine (6.00 mL, 42.0 mmol, 1.3 equiv.) and cyclohexanone (3.14 g, 32.0 mmol, 1.0 equiv.) were added to the resulting pale orange solution. The reaction mixture was stirred for 16 hours, while warming to room temperature, and then heated to reflux for a further 2 hours. Purification by chromatography column (pentane) of the crude (R_f = 0.7, pentane) followed by trap-to-trap transfer yielded a colourless liquid (2.98 g, 18.5 mmol, 58% yield).

^{1}H NMR (600 MHz, $CDCl_3$): δ 6.03 (tt, $^3J_{HH}$ = 4.0 Hz, $^4J_{HH}$ = 1.7 Hz, 1H), 2.44 – 2.39 (m, 2H), 2.10 – 2.04 (m, 2H), 1.77 – 1.70 (m, 2H), 1.64 – 1.57 (m, 2H). The NMR analysis is in agreement with that reported in the literature.[4]

1.3 Procedure for the synthesis of the methylzirconocene chloride

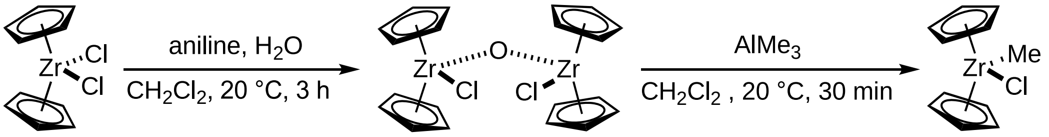

Methylzirconocene chloride was synthesised following a reported procedure.[5]

First step: synthesis of [($Cp_2ZrCl)_2(\mu$*-O)]*

A 250 mL Schlenk flask was charged with Cp_2ZrCl_2 (10.00 g, 34.2 mmol, 1.0 equiv.), CH_2Cl_2 (80 mL) followed by aniline (3.4 mL, 37.2 mmol, 1.1 equiv.). After stirring for 5 minutes, distilled water (0.43 mL, 23.8 mmol, 0.7 equiv.) was added and a white solid of aniline hydrochloride salt was observed instantly. Stirring was continued for about 3 hours at 20 °C. The flask was placed in the fridge (+4 °C) for 15 hours. Cold filtration under argon of the resulting mixture afforded a clear yellowish filtrate. The remaining solid was washed once with cold (0 °C) CH_2Cl_2 (10 mL) and the combined CH_2Cl_2 layers were concentrated to a yellowish solid. Dry pentane (11 mL) was added and the suspension was stirred for 20 minutes. The supernatant was filtered-off and the remaining white solid was washed once with dry pentane (11 mL) and dried under high vacuum for 1 hour to afford the zirconium bridged oxide [$(Cp_2ZrCl)_2(\mu$-O)] (7.04 g, 13.3 mmol, 78% yield) as a pale pink solid.

^{1}H NMR (400 MHz, C_6D_6): δ 6.02 (s, Cp).

Second step: synthesis of the methylzirconocene chloride

To a 100 mL Schlenk flask containing a suspension of [$(Cp_2ZrCl)_2(\mu$-O)] (7.04 g, 13.3 mmol, 1.0 equiv.) in dry CH_2Cl_2 (65 mL) was added a trimethylaluminium solution (2M in hexane, 16.7 mL, 33.3 mmol, 2.5 equiv.) at 20 °C. After a few seconds the mixture became a homogenous yellowish solution, no exotherm was observed. After 30 minutes, dry Et_2O (26 mL) was added, and stirring was stopped after 2 minutes. The solvent was slowly evaporated until the first yellow solids started to precipitate (about 3–5 mL left in the flask). Stirring on, dry pentane (26 mL) was added. After 10 minutes stirring, the white solid obtained was filtered under argon and washed twice with dry pentane (17 mL each) and finally dried under vacuum for 3 hours to afford the methylzirconocene chloride (4.47 g, 16.5 mmol, 62%) as a white solid.

^{1}H NMR (400 MHz, C_6D_6): δ 5.74 (s, 10H, Cp), 0.45 (s, 3H, CH_3). The NMR analysis is in agreement with that reported in the literature.[5]

1.4 Procedure for the synthesis of complex 1

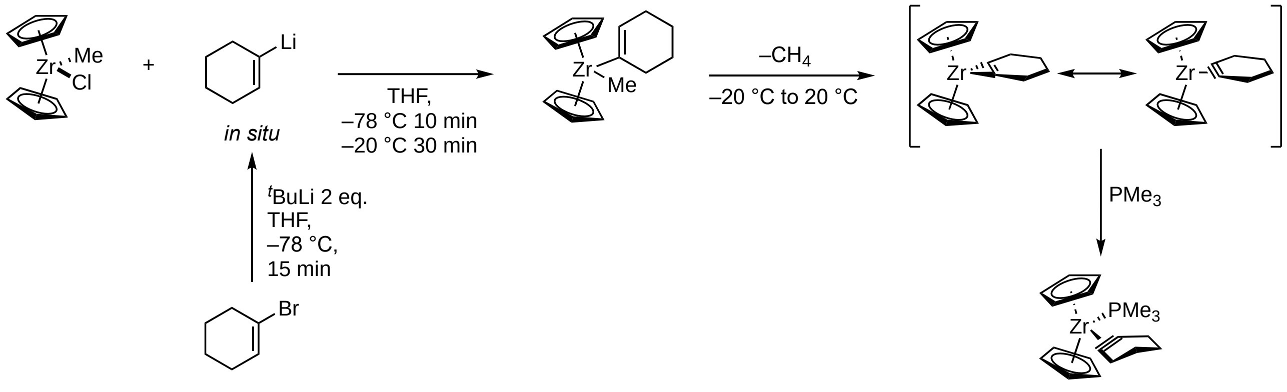

1

yield: 52%

Complex **1** was prepared according to a reported procedure.[6] To a Schlenk flask under argon was added dry THF (21 mL), which was cooled to –78 °C. ^{t}BuLi in pentane 1.72 M (5.8 mL, 10.7 mmol, 2.1 equiv.) was added with stirring. 1-Bromocyclohexene (0.86 g, 5.4 mmol, 1.1 equiv.) was added to the solution dropwise, *via* syringe and the yellow reaction mixture was allowed to stir at –78 °C for 15 minutes. The 1-lithiocyclohexene, so formed, was added dropwise *via* cannula to a –78 °C solution of methylzirconocene chloride (1.44 g, 5.1 mmol, 1.0 equiv.) in THF (28 mL). The reaction mixture was stirred for 10 minutes at –78 °C and was then warmed to –20 °C and stirred for an additional 10 min. At this point, trimethylsilyl chloride (0.05 mL, 0.3 mmol, 0.1 equiv.) was added *via* syringe (to destroy excess alkenyllithium) and the brown reaction mixture was stirred at –20 °C for additional 10 minutes. Trimethylphosphine (0.81 mL, 8.0 mmol, 1.6 equiv.) was added *via* syringe and the deep red reaction mixture was allowed to stir at 20 °C for 16 hours. The resulting mixture was concentrated *in vacuo* and the solid residue was extracted with toluene (7 mL) and filtered under argon. The remaining lithium salts were washed with five portions of toluene (10 mL each) and the toluene solution was concentrated to dryness to afford **6** (1.00 g, 2.7 mmol, 52%) as a dark red solid.

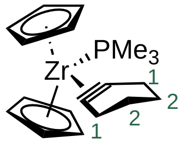

^{1}H NMR (400 MHz, C_6D_6): δ 5.25 (d, J_{HP} = 5.8 Hz, 10H, Cp), 2.85 (t, $^3J_{HH}$ = 5.9 Hz, 2H, 1-*H*), 2.28 (tt $^3J_{HH}$ = 5.8 Hz, $^4J_{HH}$ = 1.7 Hz, 2H, 1-*H*), 1.77 – 1.62 (m, 4H, 2-*H*), 0.96 (d, $^2J_{HP}$ = 5.8 Hz, 9H, PC*H*$_3$).

^{31}P NMR (243 MHz, C_6D_6): δ –2.22 (bs).

The NMR analysis is in agreement with that reported in the literature.[6]

1.5 Procedure for the stability test of complex 1

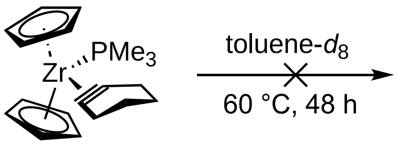

1

The following manipulation was carried out in an Ar-filled glovebox. Complex **1** (15.0 mg, 0.04 mmol) was dissolved in toluene-d_8 (0.7 mL) in an NMR tube equipped with a J. S. Young's valve. The mixture was heated up to 60 °C for 48 hours and analysed by ^{1}H and ^{31}P NMR spectroscopy, which showed that no decomposition occurred.

1.6 Procedure for the synthesis of complex 3

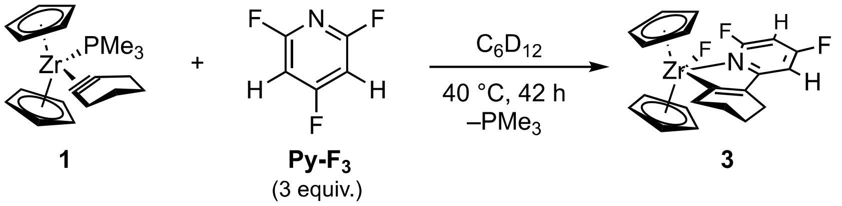

The following manipulation was carried out in an Ar-filled glovebox. Complex **1** (62.0 mg, 0.16 mmol, 1.0 equiv.) was dissolved in cyclohexane-d_{12} (0.7 mL) in an NMR tube equipped with a J. S. Young's valve. 2,4,6-trifluoropyridine (39.9 µL, 0.48 mmol, 3.0 equiv.) was added and the mixture was kept at 40 °C for 42 hours. The product was dried under reduced pressure to afford **3** as a dark red solid (49.4 mg, isolated yield 71%). 50 mg of the solid were dissolved in toluene and set for crystallisation by vapor diffusion with tetramethylsilane as antisolvent. The obtained crystals were suitable for X-ray diffraction analysis and are reported in section 3.1.

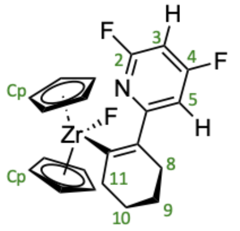

^{1}H NMR (400 MHz, C_6D_6): 6.35 (dd, 1H, $^3J_{HF}$ = 9.7 Hz, $^4J_{HH}$ = 2.3 Hz, 3-*H*), 5.96 (s, 10H, Cp), 5.58 (mc, 1H, 5-*H*), 2.96 (mc, 2H, 11-*H*), 1.94 (mc, 2H, 8-*H*), 1.66 (mc, 4H, 9-*H*, 10-*H*).

^{19}F NMR (377 MHz, C_6D_6): 35.3 (s, 1F, F–Zr), –60.7 (mc, 1F, 6-*F*), –94.7 (mc, 1F, 4-*F*).

^{13}C{^{1}H} NMR (101 MHz, C_6D_6): 215 (d, $^2J_{CF}$ = 3.7 Hz, 12-*C*), 185 (s, 7-*C*), 172.23 (dd, $^1J_{CF}$ = 264.1 Hz, $^3J_{CF}$ = 15.1 Hz, 6-*C*), 163 (dd, $^1J_{CF}$ = 246.2 Hz, $^3J_{CF}$ = 15.1 Hz, 4-*C*), 138 (d, $^3J_{CF}$ = 3.1 Hz, 2-*C*), 111 (s, Cp), 102 (dd, $^2J_{CF}$ = 19.4 Hz, $^4J_{CF}$ = 3.4 Hz, 3-*C*), 92.4 (dd, $^2J_{CF}$ = 38.6 Hz, $^2J_{CF}$ = 24.4 Hz, 5-*C*), 38.3 (d, $^3J_{CF}$ = 14.3 Hz, 11-*C*), 27.6 (s, 10-*C*), 24.4 (d, $^4J_{CF}$ = 3.6 Hz, 8-*C*), 23.5 (s, 9-*C*).

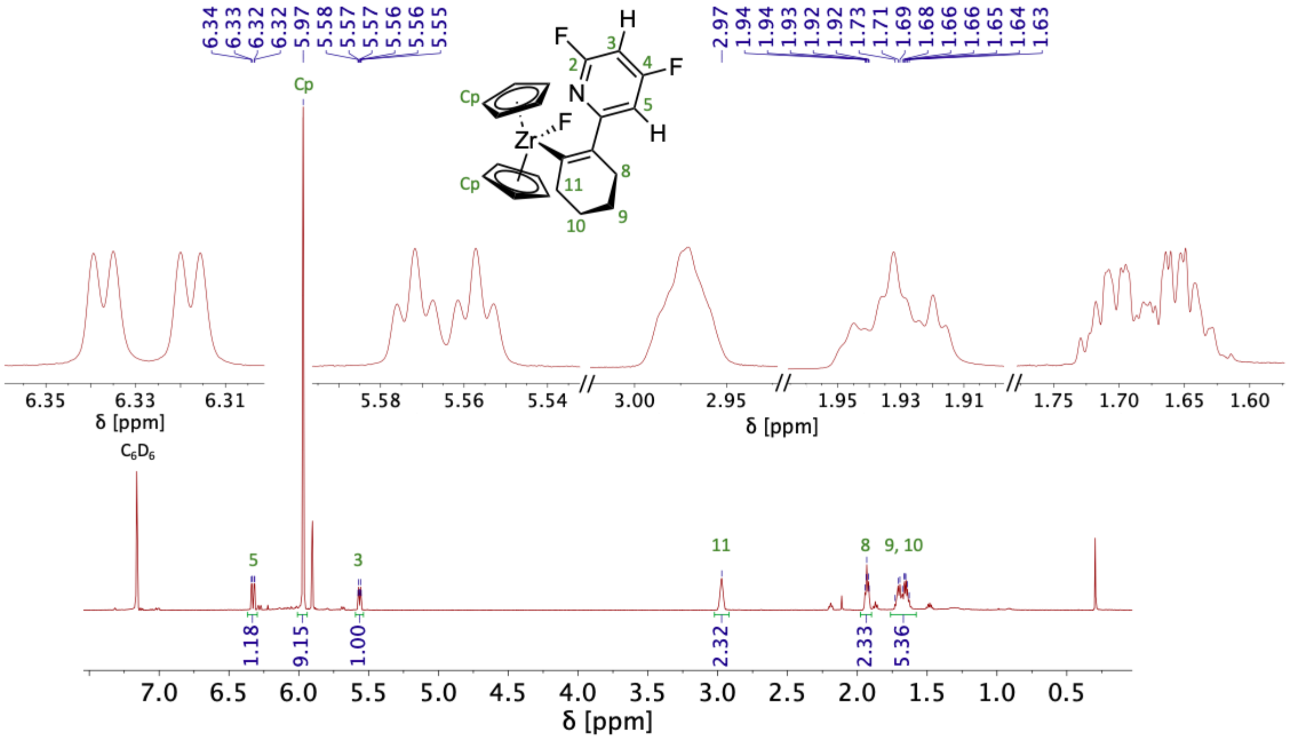

Figure S1 ^{1}H NMR (500 MHz, C_6D_6) spectrum of the crude reaction mixture of the reaction between the zirconocene-PMe_3 adduct **1** (1 equiv.) with **Py-F$_3$** (3 equiv.) in cyclohexane at 40 °C for 42 hours, dried and dissolved in C_6D_6. Magnifications of the signals of complex **3** are given.

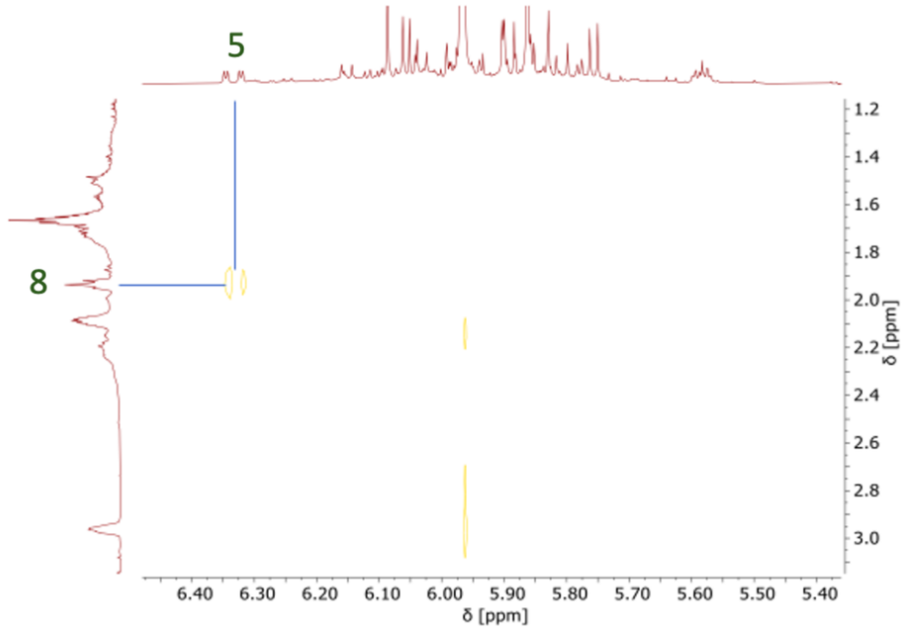

Figure S2 ^{1}H NOESY (500 MHz, C_6D_6) spectrum of the crude reaction mixture of the reaction between the zirconocene-PMe_3 adduct **1** (1 equiv.) with **Py-F$_3$** (3 equiv.) in cyclohexane at 40 °C for 42 hours, dried and dissolved in C_6D_6. The spatial correlation between protons in position 5 and 8 is highlighted.

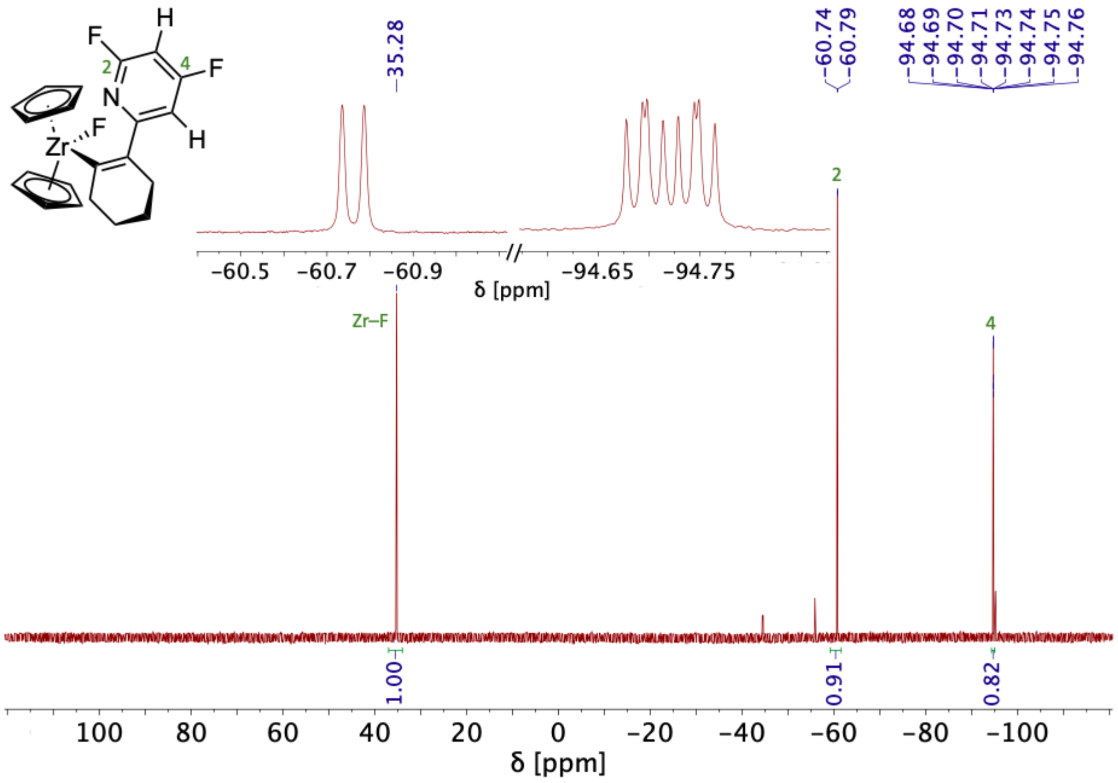

Figure S3 ^{19}F NMR (377 MHz, C_6D_6) spectrum of the crude reaction mixture of the reaction between the zirconocene-PMe_3 adduct **1** (1 equiv.) with **Py-F$_3$** (3 equiv.) in cyclohexane at 40 °C for 42 hours, dried and dissolved in C_6D_6. Magnifications of the signals of complex **3** are given.

1.7 Procedure for the synthesis of 2,4-difluoro-6-(2-iodocyclohexen-1-yl)-pyridine

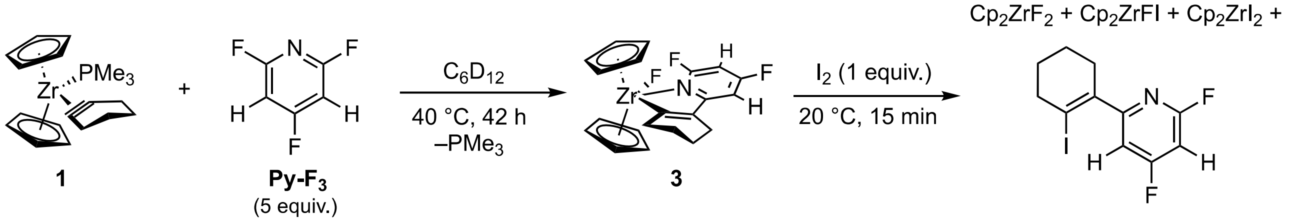

The following manipulation was carried out in an Ar-filled glovebox. Complex **1** (62.0 mg, 0.16 mmol, 1.0 equiv.) was dissolved in cyclohexane-d_{12} (0.7 mL) in an NMR tube equipped with a J. S. Young's valve. 2,4,6-trifluoropyridine (68.2 µL, 0.82 mmol, 5.0 equiv.) was added and the mixture was kept at 40 °C for 42 hours. The dark solid was filtered, the filtrate was dried under reduced pressure and dissolved in 0.6 mL of C_6D_6. I_2 (40.0 mg, 0.16 mmol, 1.0 equiv.) was added to get a mixture of 2,4-difluoro-6-(2-iodocyclohexen-1-yl)-pyridine, Cp_2ZrF_2, Cp_2ZrFI, Cp_2ZrI_2 after 30 min reaction time at 20 °C.

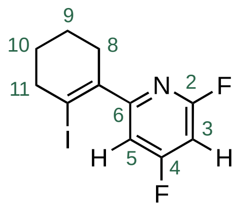

2,4-difluoro-6-(2-iodocyclohexen-1-yl)-pyridine:

^{1}H NMR (400 MHz, C_6D_6): 6.63 (ddd, $^3J_{HF}$ = 8.5 Hz, J_{HH} = 1.9 Hz, $^5J_{HF}$ = 0.7 Hz, 1H, 5-*H*), 5.90 – 5.83 (m, 1H, 3-*H*), 2.50 – 2.41 (m, 2H, 11-*H*), 2.31 – 2.20 (m, 2H, 8-*H*), 1.40 – 1.30 (m, 1H, 10-*H*), 1.24 – 1.18 (m, 2H, 9-*H*).

^{19}F NMR (377 MHz, C_6D_6): –63.3 (d, $^3J_{HF}$ = 22.6 Hz, 1F, 2-*F*), –97.9 (mc, 1F, 4-*F*).

^{13}C{^{1}H} NMR (101 MHz, C_6D_6): 174.2 (d, $^1J_{CF}$ = 290.1 Hz, 2-*C*), 165.1 (d, $^1J_{CF}$ = 192.2 Hz, 4-*C*), 142.4 (d, $^3J_{CF}$ = 3.4 Hz, 6-*C*), 110.1 (dd, J_{CF} = 18.9 Hz, J_{CF} = 5.5 Hz, 5-*C*), 100.3 (s, 7-*C*), 96.1 (dd, J_{CF} = 42.5 Hz, J_{CF} = 22.3 Hz, 3-*C*), 41.6 (s, 8-*C*), 31.6 (s, 11-*C*), 25.0 (s, 10-*C*), 21.9 (s, 9-*C*).

GC(EI)-MS: $[C_{11}H_{10}F_2IN]^{\bullet+}$ 321, $[C_{11}H_{10}F_2N]^{\bullet+}$ 194 *m/z.*

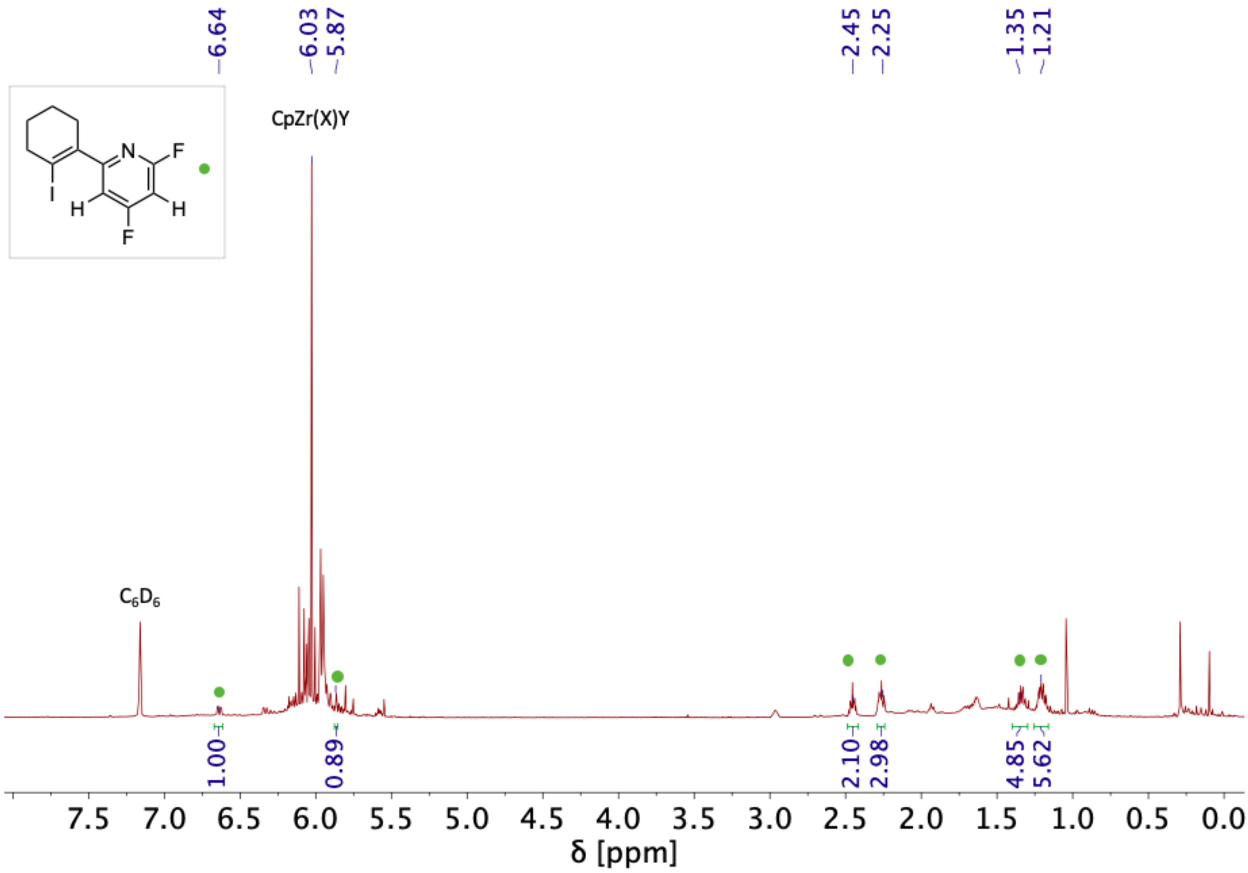

Figure S4 ^{1}H NMR (400 MHz, C_6D_6) spectrum of the iodinolysed (I_2, 1 equiv.) crude reaction mixture of the reaction between the zirconocene-PMe_3 adduct **1** (1 equiv.) with **Py-F$_3$** (3 equiv.) in cyclohexane at 40 °C for 42 hours.

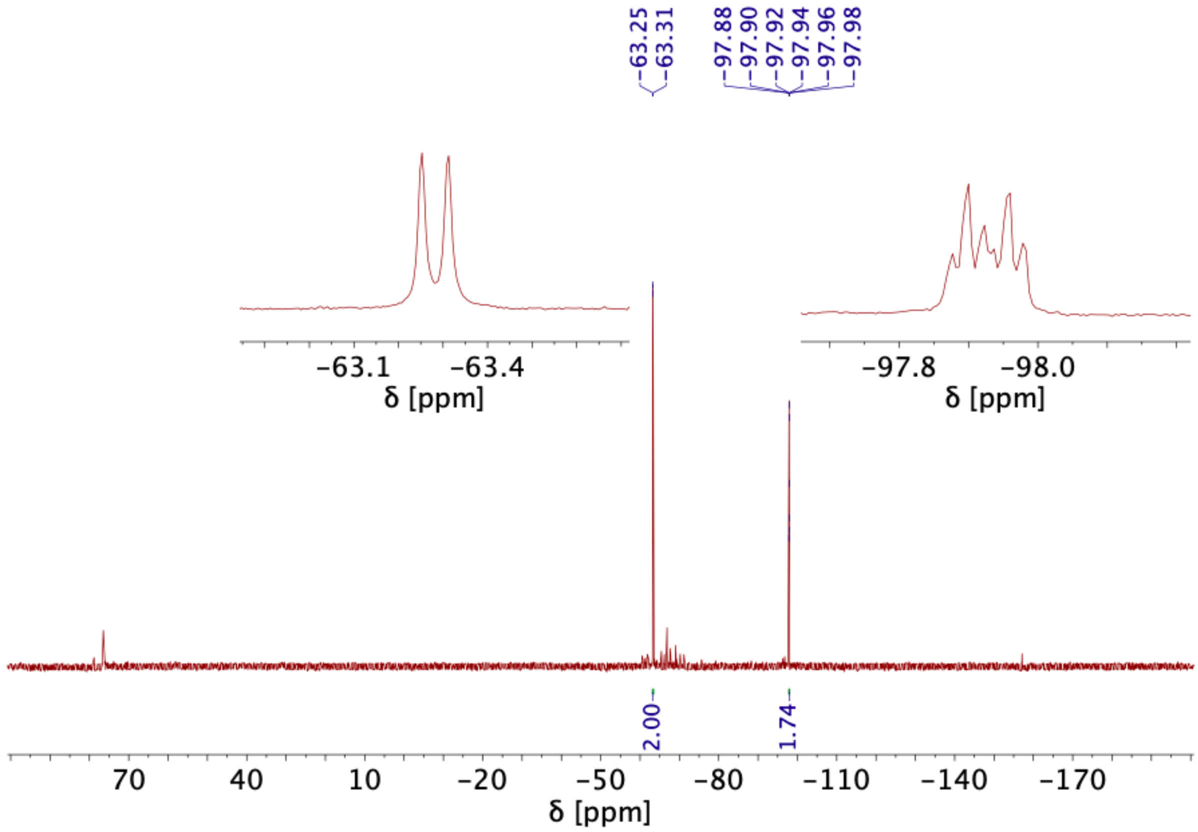

Figure S5 ^{19}F NMR (377z MHz, C_6D_6) spectrum of the iodinolysed (I_2, 1 equiv.) crude reaction mixture of the reaction between the zirconocene-PMe_3 adduct **1** (1 equiv.) with **Py-F$_3$** (3 equiv.) in cyclohexane at 40 °C for 42 hours. Magnifications of the signals of complex **3** are given.

1.8 Procedure for the solvent optimisation in the synthesis of complex 3

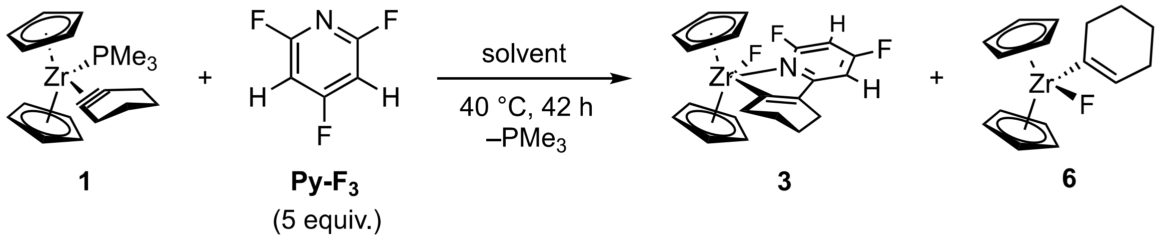

The following manipulation was carried out in an Ar-filled glovebox. Complex **1** (62.0 mg, 0.16 mmol, 1.0 equiv.) was dissolved in 0.7 mL of the tested solvent (i.e., C_6D_6, toluene-d_8, cyclohexane, chlorobenzene, Et_2O, THF-d_8) in an NMR tube equipped with a J. S. Young's valve. 2,4,6-trifluoropyridine (67.7 µL, 0.82 mmol, 5.0 equiv.) was added. The mixture was kept at 40 °C and monitored over time by ^{1}H, ^{31}P and ^{19}F NMR spectroscopy.

Solvent	**6** : **3** ratio[a]
Tetrahydrofuran	65 : 35
Chlorobenzene-*d*5	45 : 55
Toluene-*d*8	39 : 61
Benzene-*d*6	39 : 61
Diethyl ether	24 : 76
Cyclohexane	3 : 97

Table S1 Conditions: 0.04 mmol of complex **3** (1 equiv.), 0.04 mmol of **Py-F$_3$** (1 equiv.), 0.7 mL of solvent, 40 °C, 48 hours.
[a] Determined by ^{19}F NMR spectroscopy.

1.9 Procedure for the synthesis of complex 2

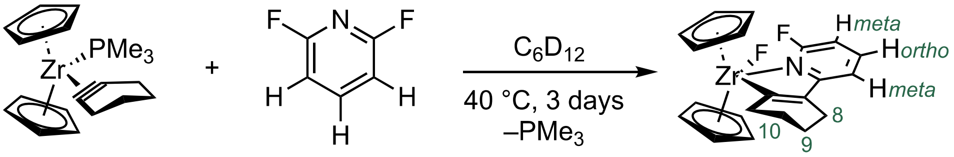

1 **Py-F₂** (3 equiv.) **2**

The following manipulation was carried out in an Ar-filled glovebox. Complex **1** (15.0 mg, 0.04 mmol, 1.0 equiv.) was dissolved in cyclohexane-d_{12} (0.7 mL) in an NMR tube equipped with a J. S. Young's valve. 2,6-difluoropyridine (10.8 µL, 0.12 mmol, 3.0 equiv.) was added and product **2** was obtained in 72% NMR yield after 3 days at 40 °C. The reaction evolution was monitored by ^{1}H, ^{31}P and ^{19}F NMR spectroscopy.

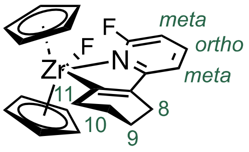

^{1}H NMR (500 MHz, C_6D_{12}): 7.74 (*pseudo* q, *J* = 7.8 Hz, 1H, *para-H*), 7.02 (*pseudo* dt, $^{3}J_{HH}$ = 7.8 Hz, *J* = 1.2 Hz, 1H, *meta-H*), 6.57 (*pseudo* dt, $^{3}J_{HH}$ = 7.9 Hz, *J* = 1.2 Hz, 1H, *meta-H*), 5.93 (s, 10H, Cp), 2.60 – 2.53 (m, 2H, 11-*H*), 2.27 (tt, $^{3}J_{HH}$ = 6.4 Hz, $^{4}J_{HH}$ = 2.2 Hz, 2H, 8-*H*), 1.76 – 1.70 (m, 2H, 9-*H*), 1.54 – 1.49 (m, 2H, 10-*H*).

^{1}H NMR (500 MHz, C_6D_6): 6.89 (*pseudo* q, *J* = 7.9 Hz, 1H, *para-H*), 6.55 (*pseudo* dt, $^{3}J_{HH}$ = 7.9 Hz, *J* = 1.2 Hz, 1H, *meta-H*), 6.02 (s, 10H, Cp), 5.89 (*pseudo* dt, $^{3}J_{HH}$ = 8.0 Hz, *J* = 1.2 Hz, 1H, *meta-H*), 3.04 – 2.99 (m, 2H, 11-*H*), 2.17 (tt, $^{3}J_{HH}$ = 6.4 Hz, $^{4}J_{HH}$ = 2.2 Hz, 2H, 8-*H*), 1,84 – 1.77 (m, 2H, 9-*H*), 1.76 – 1.70 (m, 2H, 10-*H*).

^{19}F NMR (471 MHz, C_6D_{12}): 33.7 (s, 1F, F–Zr), –64.5 (d, *J* = 7.6 Hz, 1F, *ortho-F*).

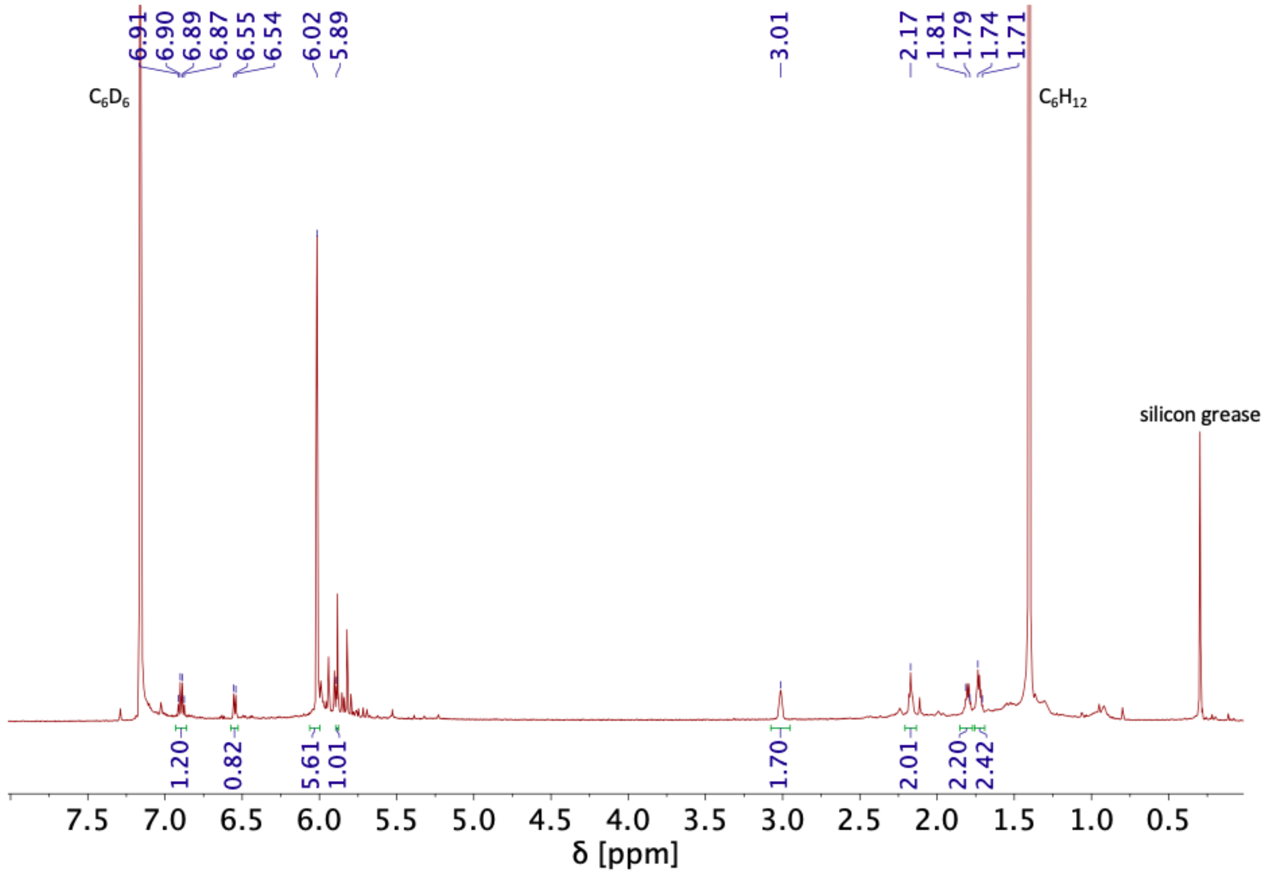

Figure S6 ^{1}H NMR (600 MHz, C_6D_6) spectrum of the crude reaction mixture of the reaction between the zirconocene-PMe_3 adduct **1** (1 equiv.) with **Py-F$_2$** (3 equiv.) in cyclohexane at 40 °C for 3 days, dried and dissolved in C_6D_6.

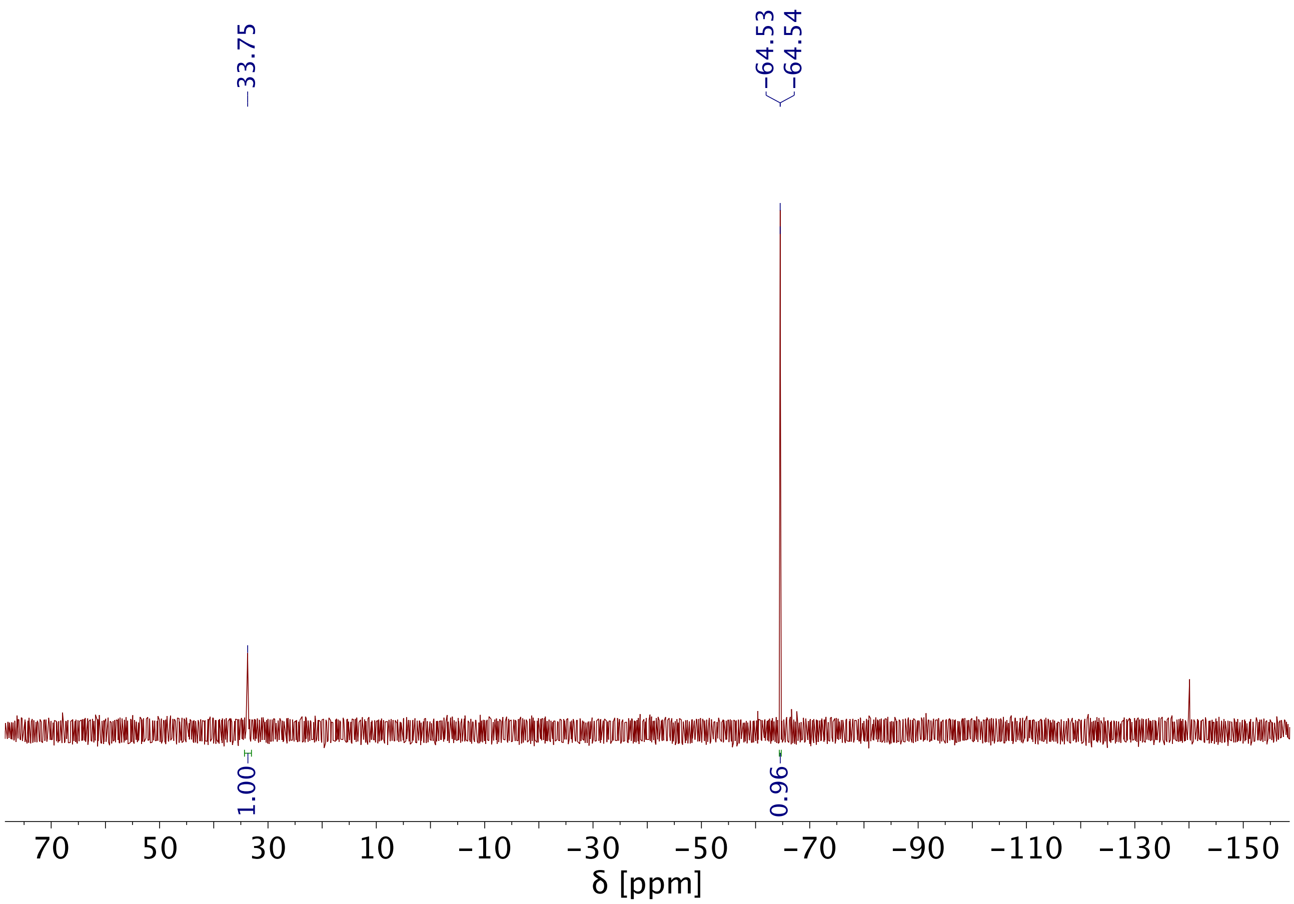

Figure S7 ^{19}F NMR (565 MHz, C_6D_6) spectrum of the crude reaction mixture of the reaction between the zirconocene-PMe_3 adduct **1** (1 equiv.) with **Py-F$_2$** (3 equiv.) in cyclohexane at 40 °C for 3 days, dried and dissolved in C_6D_6.

1.10 Procedure for the synthesis of complex 5 and its iodinolysis

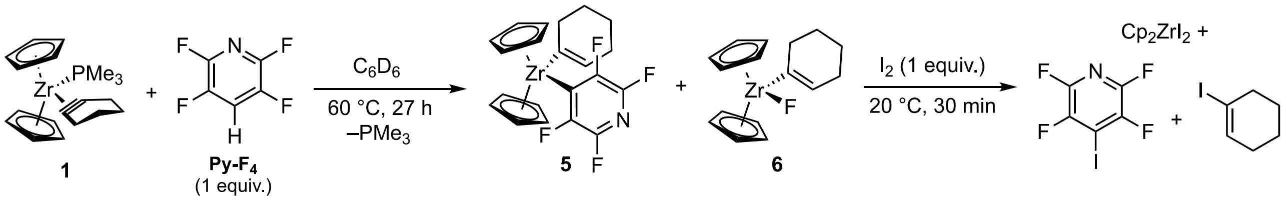

The following manipulation was carried out in an Ar-filled glovebox. Complex **1** (15.0 mg, 0.04 mmol, 1.0 equiv.) was dissolved in cyclohexane (0.7 mL) in an NMR tube equipped with a J. S. Young's valve. 2,3,5,6-tetrafluoropyridine (4.2 µL, 0.04 mmol, 1.0 equiv.) was added and the mixture was kept at 60 °C and monitored by ^{1}H, ^{31}P and ^{19}F NMR. A small amount of dark red solid was formed during the reaction. After 48 hours at 60 °C, complex **5** (45% NMR yield), **6** (6% NMR yield) and unreacted **1** (9%) were observed in the ^{1}H and ^{19}F NMR spectra. At longer times, a decrease of the concentration of **5** along with an increase of the concentration of **6** were observed (e.g., after 48 hours at 60 °C, **5** was present in 45% NMR yield, **6** in 9% NMR yield). This mixture was dried under reduced pressure, dissolved in 0.6 mL of C_6D_6 and I_2 (9.6 mg, 0.04 mmol, 1 equiv.) was added to afford a mixture of 2,4-difluoro-6-(2-iodocyclohexen-1-yl)-pyridine and Cp_2ZrI_2 after 30 min reaction time at 20 °C.

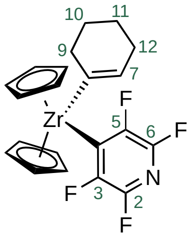

^{1}H NMR (400 MHz, C_6D_6): δ 5.40 (s, 10H, Cp), 5.09 – 5.00 (m, 1H, 7-*H*), 2.40 – 2.31 (m, 2H, 9-*H*), 1.97 – 1.87 (m, 2H, 12-*H*), 1.53 – 1.38 (m, 4H, 10-*H*, 11-*H*).

^{19}F NMR (377 MHz, C_6D_6): –97.7 (mc, 2F, *ortho*-F), –114.8 (mc, 2F, *meta*-F).

1-iodocyclohexene:

^{1}H NMR (400 MHz, C_6D_6): 6.17 – 6.13 (m, 1H, vinyl-*H*), 2.31 – 2.25 (m, 2H, CH_2), 1.68 – 1.60 (m, 2H, CH_2), 1.26 – 1.18 (m, 2H, CH_2).

^{13}C{^{1}H} NMR (101 MHz, C_6D_6): 138.1 (s, 2-*C*), 97.0 (s, 1-*C*), 39.6 (s, 3-*C*), 29.0 (s, 2-*C*), 25.3 (s, 4-*C*), 20.9 (s, 4-*C*).

The NMR analysis is in agreement with that reported in the literature.[7]

2,3,5,6-tetrafluoro-4-iodopyridine:

^{19}F NMR (376 MHz, C_6D_6): –90.2 (mc, 2F, *ortho*-F), –123.5 (mc, 2F, *meta*-F).

The NMR analysis is in agreement with that reported in the literature.[8]

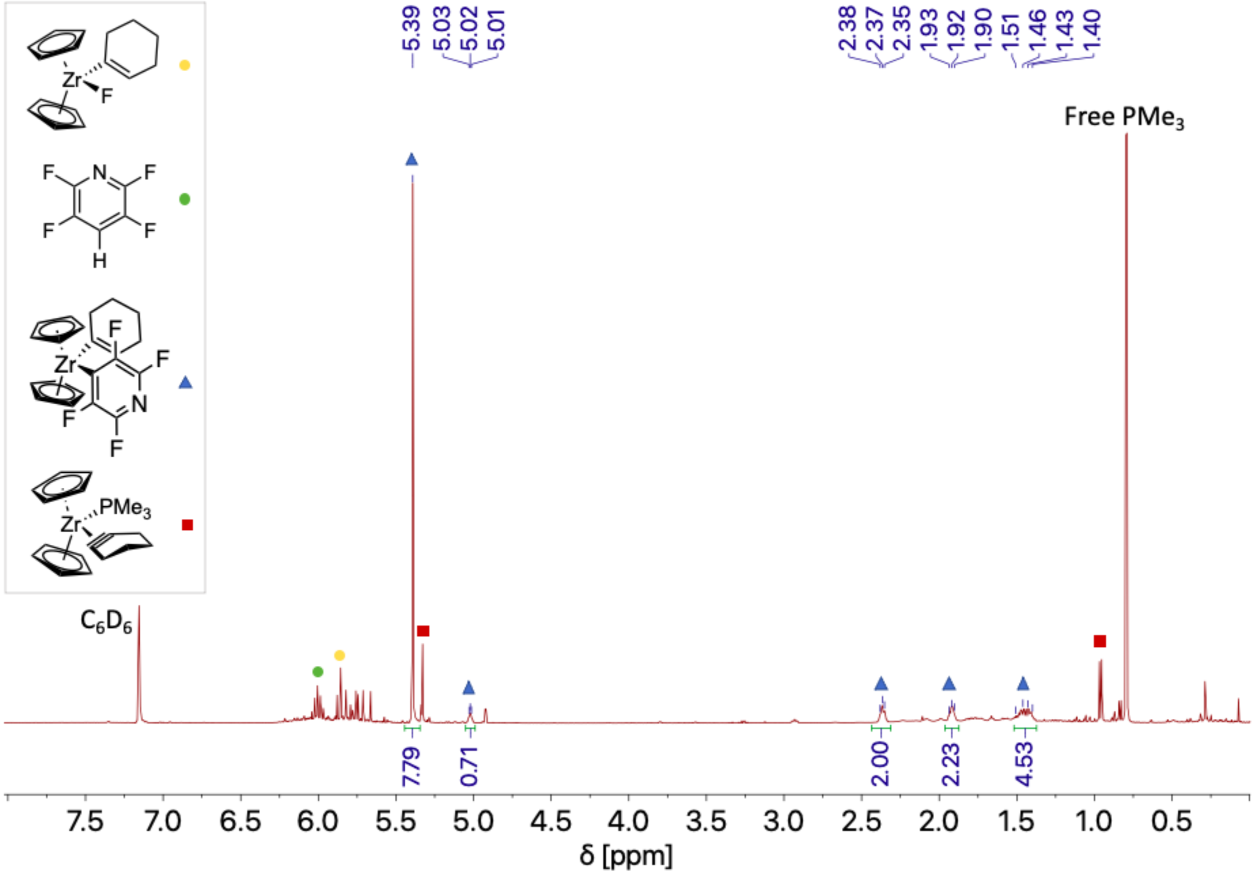

Figure S8 ^{1}H NMR (400 MHz, C_6D_6) spectrum of the crude reaction mixture of the reaction between the zirconocene-PMe_3 adduct **1** (1 equiv.) with **Py-F$_4$** (1 equiv.) in C_6D_6 at 60 °C for 27 hours.

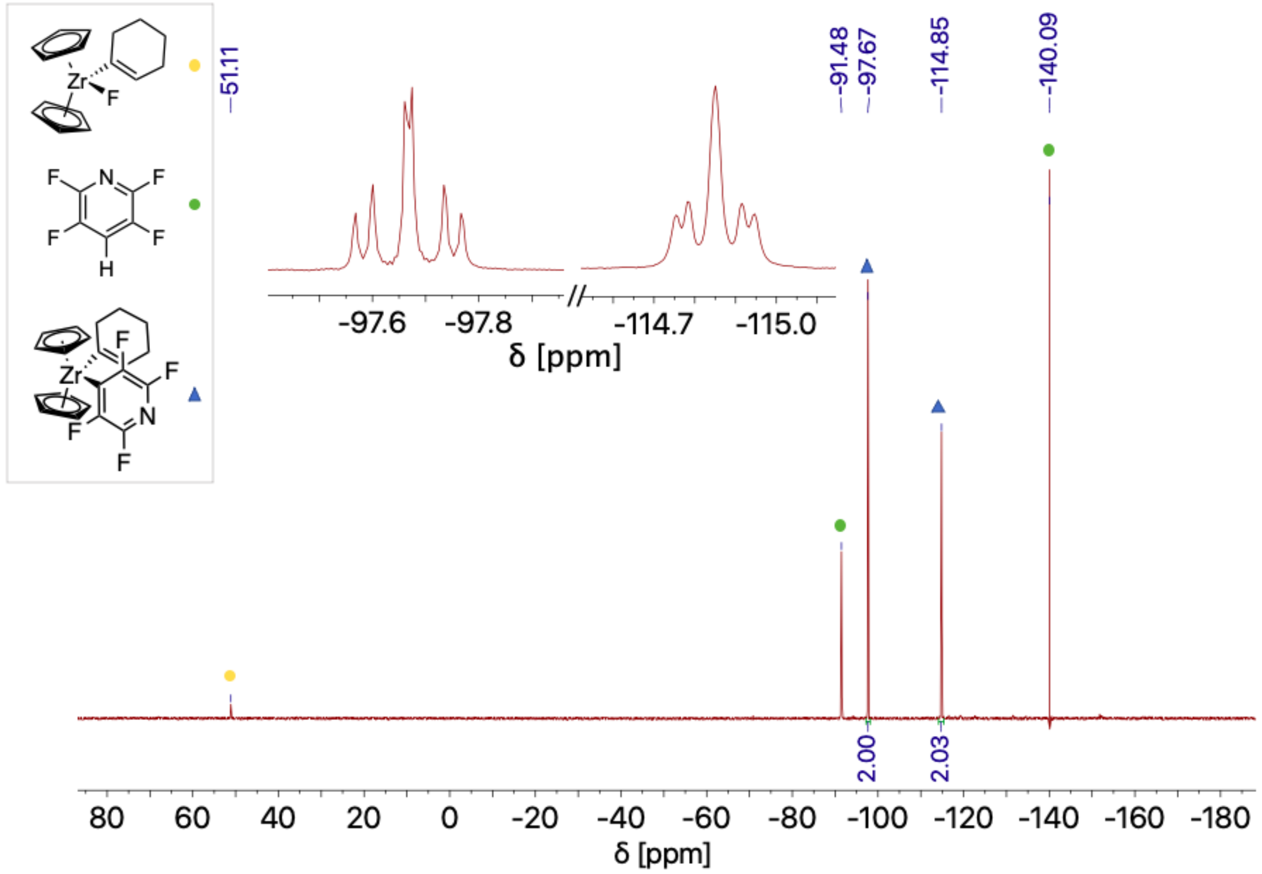

Figure S9 ^{19}F NMR (377 MHz, C_6D_6) spectrum of the crude reaction mixture of the reaction between the zirconocene-PMe_3 adduct **1** (1 equiv.) with **Py-F$_4$** (1 equiv.) in C_6D_6 at 60 °C for 27 hours.

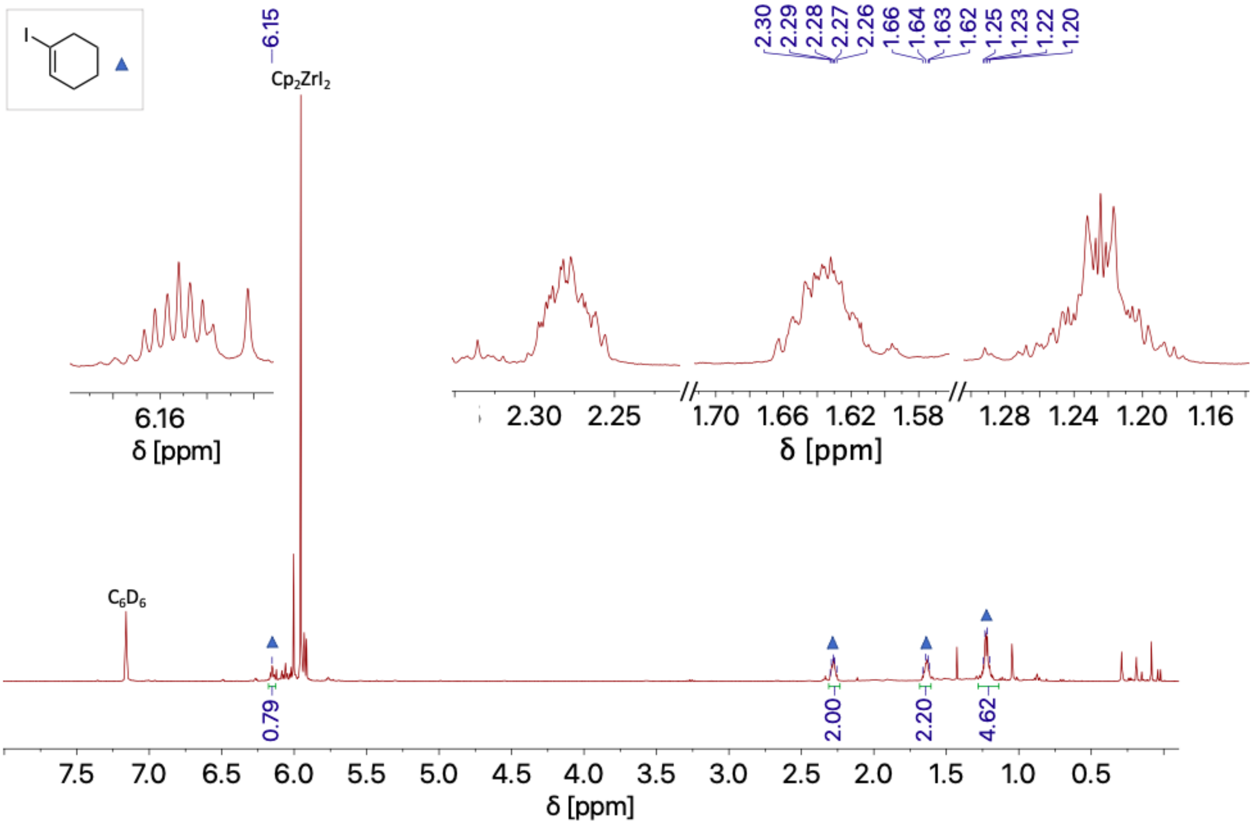

Figure S10 ^{1}H NMR (400 MHz, C_6D_6) spectrum of the crude reaction mixture of the reaction between the zirconocene-PMe_3 adduct **1** (1 equiv.) with **Py-F$_4$** (1 equiv.) in C_6D_6 at 60 °C for 27 hours, after the addition of I_2 (1 equiv., 20 °C, 30 min).

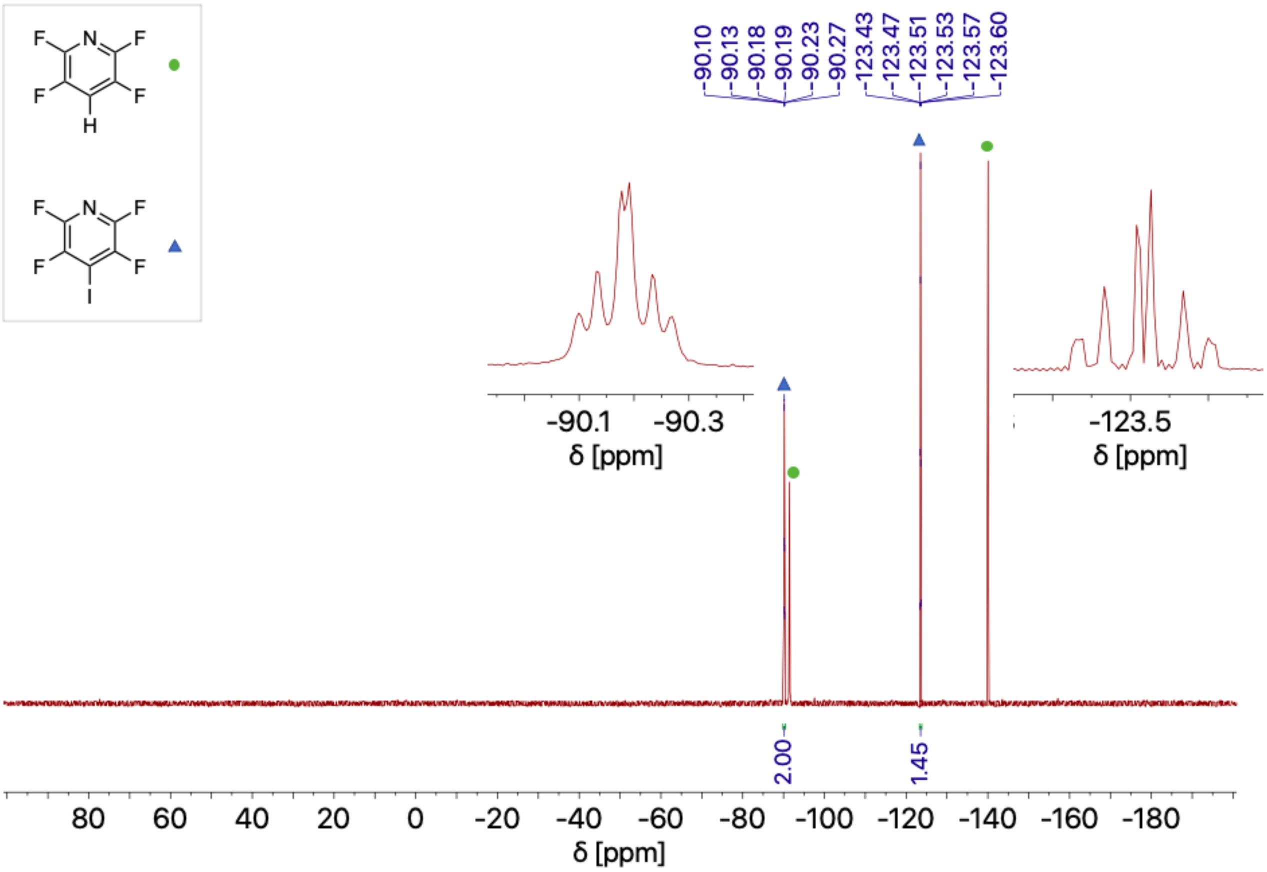

Figure S11 ^{19}F NMR (377 MHz, C_6D_6) spectrum of the crude reaction mixture of the reaction between the zirconocene-PMe_3 adduct **1** (1 equiv.) with **Py-F$_4$** (1 equiv.) in C_6D_6 at 60 °C for 27 hours, after the addition of I_2 (1 equiv., 20 °C, 30 min).

1.11 Study of the reactivity of the zirconocene-cyclohexyne transient intermediate with Py-F_5

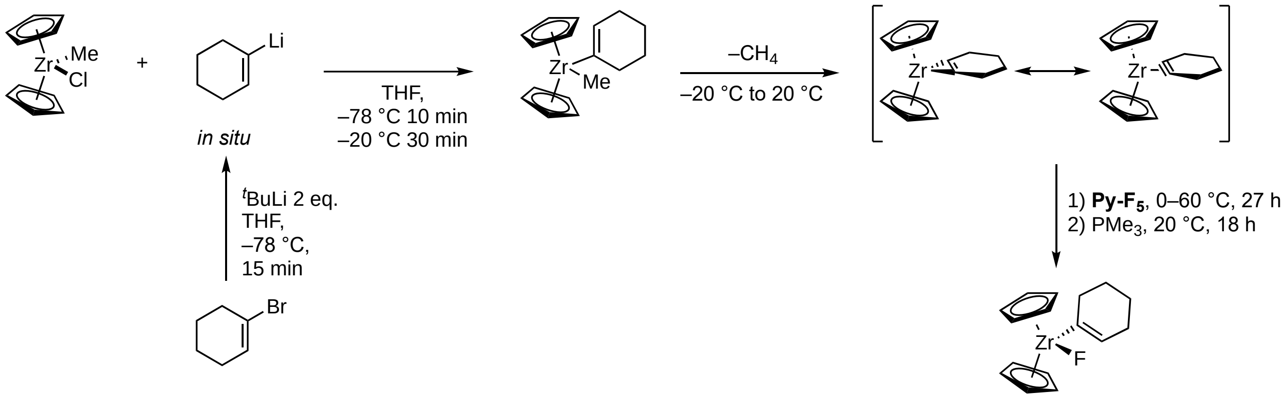

6

To a Schlenk flask under argon was added dry THF (5 mL) and cooled to –78 °C. ^{t}BuLi in pentane 1.72 M (1.6 mL, 2.7 mmol, 2.1 equiv.) was added under stirring. 1-Bromocyclohexene (0.22 g, 1.4 mmol, 1.1 equiv.) was added to the solution dropwise, *via* syringe and the yellow reaction mixture was allowed to stir at –78 °C for 15 minutes. The 1-lithiocyclohexene, so formed, was added dropwise *via* cannula to a –78 °C solution of methylzirconocene chloride (0.35 g, 1.31 mmol, 1.0 equiv.) in THF (7 mL). The reaction mixture was stirred for 10 minutes at –78 °C and was then warmed to –20 °C and stirred for an additional 10 min. **Py-F$_5$** (0.2 mL, 2.0 mmol, 1.5 equiv.) was added into the solution. The system was heated up to 60 °C for 27 hours and monitored by ^{19}F NMR spectroscopy. As no substantial change was observed in the ^{19}F NMR spectra over time, 1.6 equivalents of PMe_3 (0.81 mL, 8.0 mmol, 1.6 equiv.) were added and the system was kept at 20 °C for 18 hours. The progress of the reaction was monitored by ^{19}F and ^{31}P NMR. After this time, the formation of a dark red solid was observed. The ^{19}F NMR spectrum displayed a singlet at 51.0 ppm corresponding to complex **6** and many signals in the area of the *ortho*- and *meta*-F of the pyridine ring. The ^{31}P NMR spectrum displayed only traces of F_2PEt_3 and other difluorophosphoranes that could not be unambiguously identified.

1.12 Procedure for the synthesis of 6

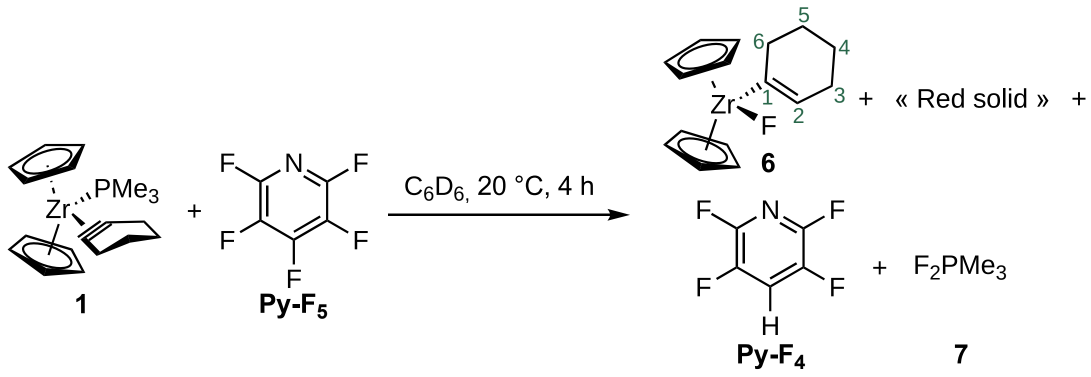

The following manipulation was carried out in an Ar-filled glovebox. Complex **6** (15.0 mg, 0.04 mmol, 1.0 equiv.) was dissolved in cyclohexane (0.7 mL) in an NMR tube equipped with a J. S. Young's valve. Pentafluoropyridine (93.3 µL of a 0.43 M in cyclohexane, 0.04 mmol, 1.0 equiv.) was added and the mixture was kept at 20 °C for 5 hours. 6.3 mg of unidentified dark red solid precipitated from the solution. ^{1}H, ^{19}F and ^{31}P NMR analyses showed the complete consumption of **1** and the formation of complex **6** (62% NMR yield), together with small amounts of **Py-F$_4$** (9% NMR yield) and **7** (1% NMR yield). Other unidentified minor species were observed by ^{19}F and ^{1}H NMR analysis.

^{1}H NMR (400 MHz, C_6D_6): 6.04 – 5.98 (m, 1H, 2-*H*), 5.86 (s, 10H, Cp), 2.14 – 2.04 (m, 4H, 3-*H*, 6-*H*), 1.70 – 1.65 (m, 4H, 4-*H*, 5-*H*).

^{19}F NMR (377 MHz, C_6D_6): 51.0 (s, Zr–*F*).

^{13}C{^{1}H} NMR (101 MHz, C_6D_6): 185 (s, 1-*C*), 126 (s, 2-*C*), 113 (s, Cp), 35.1 (s, 3-*C*), 28.3 (s, 6-*C*), 25.5 (s, 5-*C*), 23.8 (s, 4-*C*).

Elemental analyses of two different samples of the red solid: sample 1 • C 35.46%, H 2.74%, N 3.64%; sample 3 • C 34.81%, H 4.85%, N 3.50%. Low quantity of sample 2 were collected, enough for ICP-OES but not for elemental analysis

Sample	test	Zr ppm	P ppm	Mass (mg)	Vol (mL)	Zr %	P %
1	1	1.849	21.37	9.36	50	0.988	11.416
	2	1.83	21.52	9.36	50	0.978	11.496
2	3	0.903	5.008	8.91	50	0.507	2.81
	4	0.7708	4.937	8.91	50	0.433	2.77
3	5	3.219	14.95	10.8	50	1.49	6.921
	6	3.233	15	10.8	50	1.497	6.944

Table S2. ICP-OES measurements of Zr and P contents run on three different samples of the red solid.

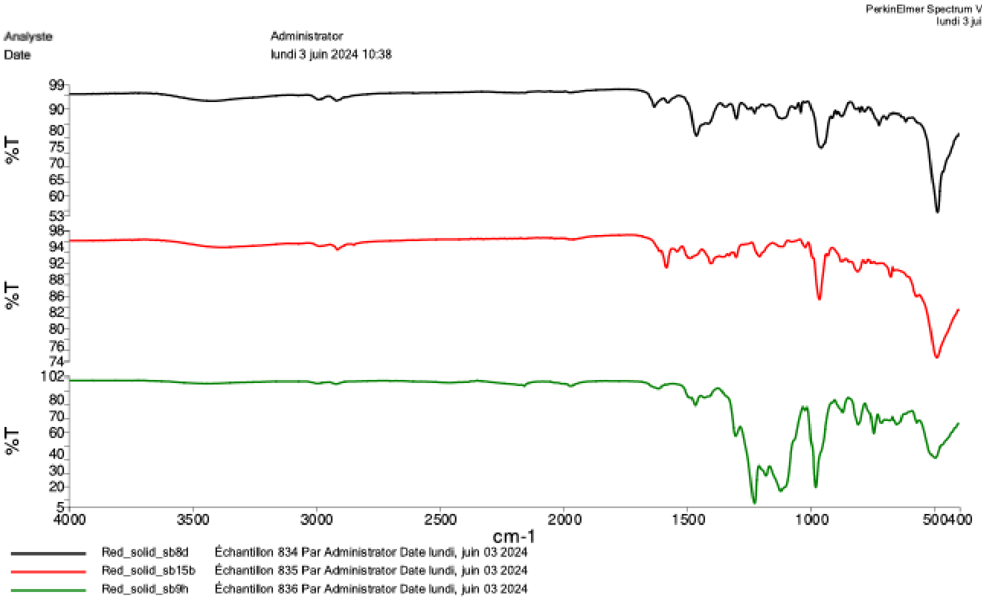

Figure S12. Stacked IR spectra of different samples of the red solid.

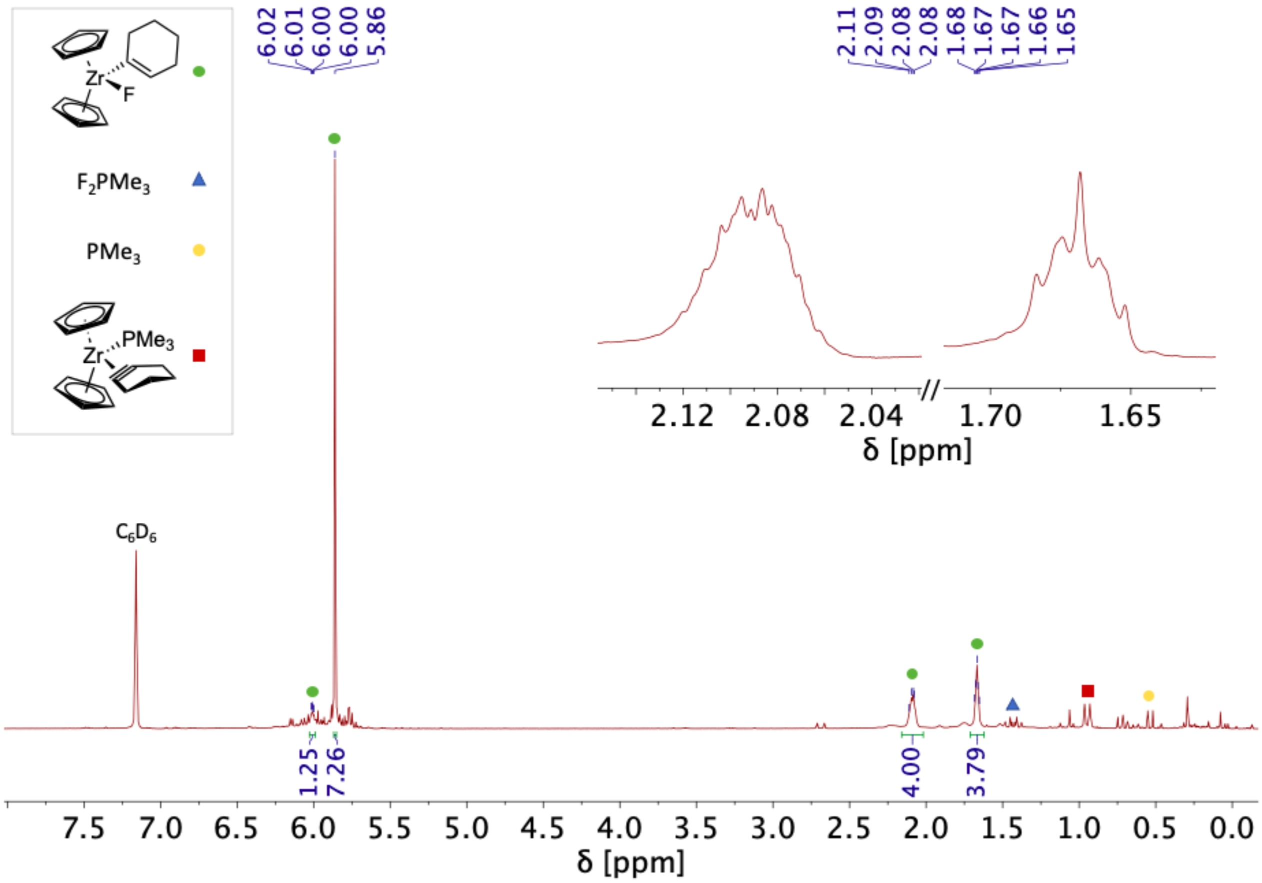

Figure S13 ^1H NMR (400 MHz, C_6D_6) spectrum of the crude reaction mixture of the reaction between the zirconocene-PMe_3 adduct **1** (1 equiv.) with **Py-F$_5$** (1 equiv.) in C_6D_6 at 20 °C after 4 hours.

1.13 Procedure for the ligand liberation of complex 6 *via* HCl addition

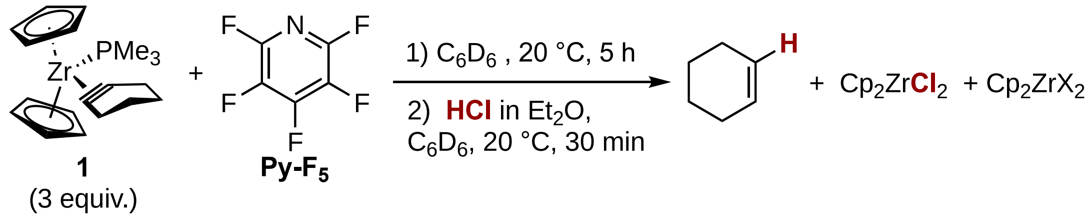

Complex **1** (15.0 mg, 0.04 mmol, 3.0 equiv.) was dissolved in cyclohexane (0.7 mL) in an NMR tube equipped with a J. S. Young's valve. Pentafluoropyridine (31.1 µL of a 0.43 M in cyclohexane, 0.01 mmol, 1.0 equiv.) was added and the mixture was kept at 20 °C for 5 hours. HCl (2M in Et_2O, 0.02 mL, 0.04 mmol, 3.0 equiv.) was added into the mixture. After 30 minutes at 20 °C, the disappearance of 1-cyclohexenylzirconocene fluoride and the formation of cyclohexene, Cp_2ZrCl_2 and other Cp_2ZrX_2 that could not be unambiguously identified were detected by ^{19}F and ^{1}H NMR spectroscopy.[9]

^{1}H NMR (400 MHz, C_6D_6): δ 5.67 (t, *J* = 1.5 Hz, 2H), 1.93 – 1.87 (m, 4H), 1.53 – 1.48 (m, 4H).

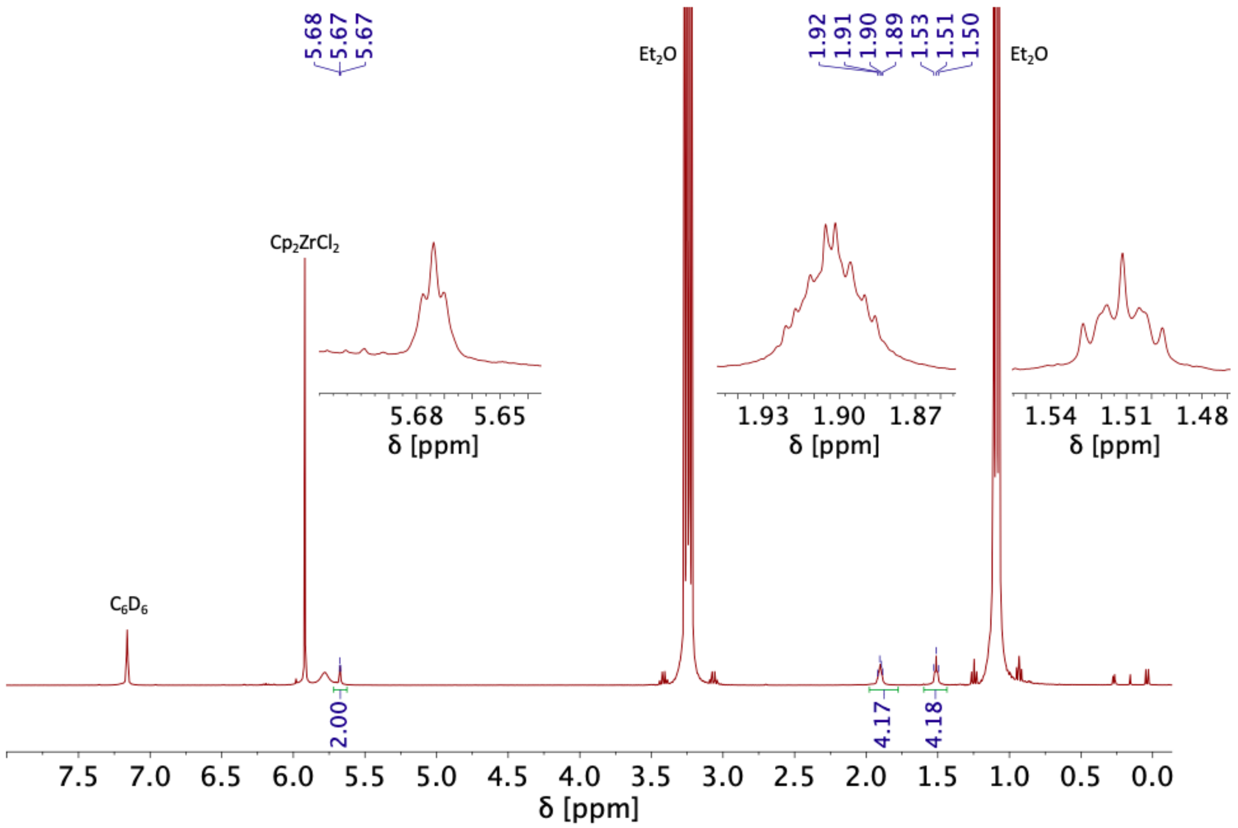

Figure S14 ^{1}H NMR (400 MHz, C_6D_6) spectrum of the crude reaction mixture obtained after 30 min at 20 °C from the addition of HCl (2M in Et_2O, 3 equiv.) to the mixture obtained in section 1.12.

1.14 Procedure for the ligand liberation of complex 6 *via* I_2 addition

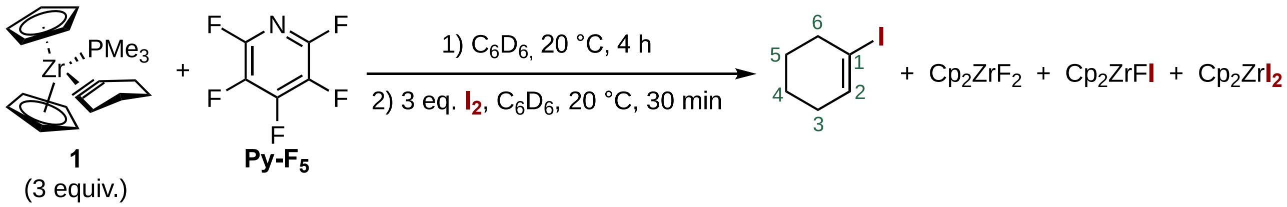

The following manipulation was carried out in an Ar-filled glovebox. Complex **1** (15.0 mg, 0.04 mmol, 3.0 equiv.) was dissolved in cyclohexane (0.7 mL) in an NMR tube equipped with a J. S. Young's valve. Pentafluoropyridine (31.1 µL of a 0.43 M in cyclohexane, 0.01 mmol, 1.0 equiv.) was added and the mixture was kept at 20 °C for 4 hours. The dark red solid formed during the reaction was filtered, the filtrate was dried under reduced pressure and dissolved in 0.6 mL of C_6D_6. I_2 (10.0 mg, 0.04 mmol, 3.0 equiv.) was added to afford a mixture of 1-iodocyclohexene, Cp_2ZrF_2, Cp_2ZrFI, Cp_2ZrI_2 after 30 min reaction time at 20 °C. The mixture was passed through a celite column to remove the Zr complexes and analysed by ^{1}H and ^{13}C NMR spectroscopy.

^{1}H NMR (400 MHz, C_6D_6): 6.15 (tt, $^{3}J_{HH}$ = 4.0 Hz, $^{4}J_{HH}$ = 1.8 Hz, 1H, 2-*H*), 2.33 – 2.23 (m, 2H, 3-*H*), 1.68 – 1.60 (m, 2H, 3-*H*), 1.25 – 1.18 (m, 4H, 4-*H*).

$^{13}C\{^{1}H\}$ NMR (101 MHz, C_6D_6): 138.1 (s, 2-*C*), 97.0 (s, 1-*C*), 39.6 (s, 3-*C*), 29.0 (s, 2-*C*), 25.3 (s, 4-*C*), 20.9 (s, 4-*C*).

The NMR analysis is in agreement with that reported in the literature.[7]

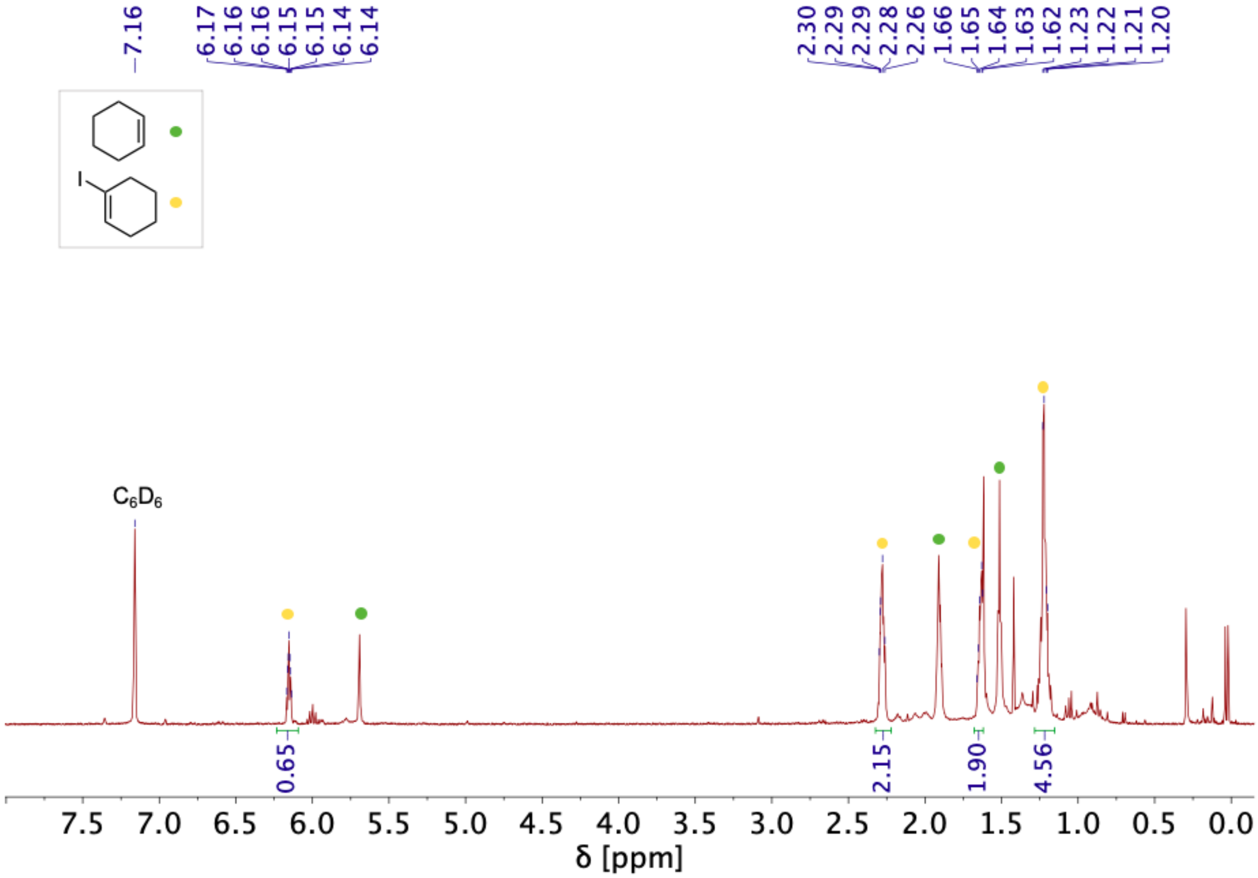

Figure S15 ^{1}H NMR (400 MHz, C_6D_6) spectrum of the reaction mixture obtained after 30 min at 20 °C from the addition of I_2 (3 equiv.) to the mixture obtained in section 1.12 and subsequently passed through a celite column.

1.15 Study on the solvent optimisation for the synthesis of complex 6

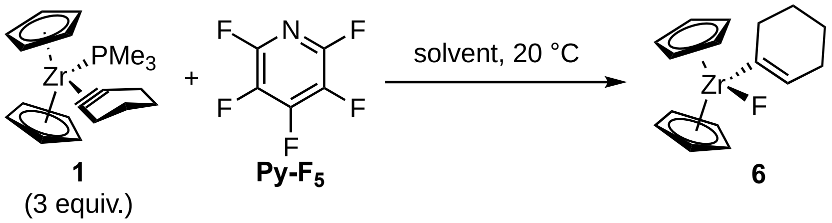

The following manipulation was carried out in an Ar-filled glovebox. Complex **1** (15.0 mg, 0.04 mmol, 3.0 equiv.) was dissolved in 0.7 mL of the tested solvent (i.e., C_6D_6, toluene-d_8, cyclohexane, THF-d_8) in an NMR tube equipped with a J. S. Young's valve. Pentafluoropyridine (31.1 µL of a 0.43 M in cyclohexane, 0.01 mmol, 1.0 equiv.) was added and the mixture was monitored over time by ^{1}H, ^{31}P and ^{19}F NMR spectroscopy. The reaction in cyclohexane proved to be slightly more selective towards **6** than the other solvent tested.

1.16 Study on the temperature optimisation for the synthesis of complex 6

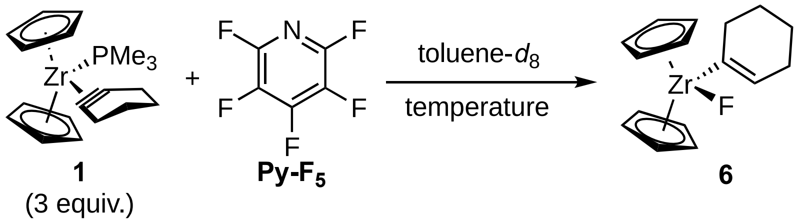

The following manipulation was carried out in an Ar-filled glovebox. Complex **1** (15.0 mg, 0.04 mmol, 3.0 equiv.) was dissolved in 0.7 mL of toluene-d_8 in an NMR tube equipped with a J. S. Young's valve. Pentafluoropyridine (31.1 µL of a 0.43 M in cyclohexane, 0.01 mmol, 1.0 equiv.) was added and the mixture was kept at variable temperatures (–40 °C, 0 °C) and monitored over time by ^{1}H, ^{31}P and ^{19}F NMR spectroscopy. No significant differences in the reaction outcome were observed.

1.17 Study on the concentration optimisation for the synthesis of complex 6

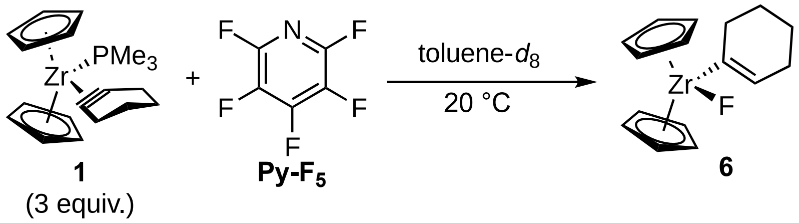

1 (3 equiv.) **Py-F5** **6**

The following manipulation was carried out in an Ar-filled glovebox. Complex **1** (0.1 M, 0.3 M) was dissolved in 0.7 mL of toluene-d_8 in an NMR tube equipped with a J. S. Young's valve. Pentafluoropyridine (0.03 M, 0.1 M) was added and the mixture was monitored over time by ^{1}H, ^{31}P and ^{19}F NMR spectroscopy. No significant differences in the reaction outcome were observed.

1.18 Study on the reagent ratio optimisation for the synthesis of complex 6

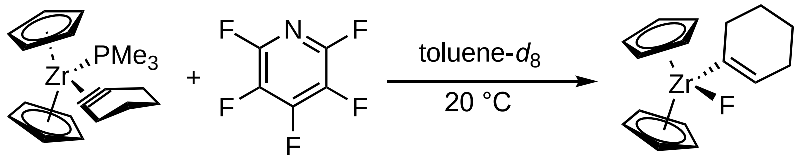

1 (X equiv.) **Py-F5** **6**

The following manipulation was carried out in an Ar-filled glovebox. Different ratios of complex **6** and pentafluoropyridine (1:1, 3:1, 1:3, 5:1) were dissolved in 0.7 mL of toluene-d_8 in an NMR tube equipped with a J. S. Young's valve. The mixture was monitored over time by ^{1}H, ^{31}P and ^{19}F NMR spectroscopy. No significant differences in the reaction outcome were observed using different reagent ratios. 3 equivalents of complex **1** were needed to consume all **Py-F$_5$**.

1.19 Procedure for the reactivity test of 1 with difluorophosphoranes 8 and F_2PMe_3

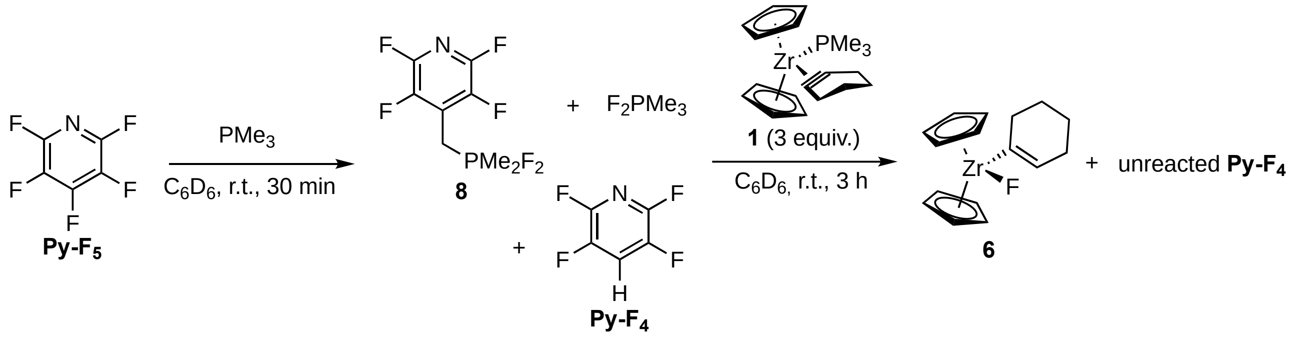

In an Ar-filled glovebox, PMe_3 (4.0 µL, 0.04 mmol, 1.0 equiv.) was added using a Hamilton® micro-syringe to C_6D_6 (0.6 mL) in an NMR tube equipped with a J. S. Young's valve. Pentafluoropyridine (4.2 µL, 0.04 mmol, 1.0 equiv.) was added using a Hamilton® micro-syringe into the solution which became instantly orange. After 30 minutes at 20 °C, complex **1** (45.2 mg, 0.12 mmol, 3.0 equiv.) was added. 3.0 equiv. of **1** were needed to completely consume **8** and F_2PMe_3 by ^{19}F NMR spectroscopy. The formation of a dark red solid was observed. **6** and unreacted **Py-F4** were identified by ^{1}H and ^{19}F NMR analysis.

Trimethyldifluorophosphorane:

^{1}H NMR (400 MHz, C_6D_6): 1.42 (dt, $^{2}J_{HP}$ = 17.3 Hz, $^{3}J_{HF}$ = 12.3 Hz, 2H, CH_3).

^{19}F NMR (377 MHz, C_6D_6): –5.5 (dm, $^{1}J_{PF}$ = 544.1 Hz, 2F, F_2P)**.**

^{31}P NMR (162 MHz, C_6D_6): –16.2 (tm, $^{1}J_{PF}$ = 544.0 Hz, 1P).

^{13}C{^{1}H} NMR (101 MHz, C_6D_6): 18.7 (dt, $^{1}J_{PC}$ = 127.3 Hz, $^{2}J_{FC}$ = 29.4 Hz, 2C, CH$_3$).

The NMR analysis is in agreement with that reported in the literature.[10]

2,3,5,6-tetrafluoropyridine (Py-F4):

^{1}H NMR (600 MHz, C_6D_6): δ 6.51 (tt, $^{3}J_{HF}$ = 7.0 Hz, $^{4}J_{HF}$ = 7.7 Hz, CH)**.**

^{19}F NMR (564 MHz, C_6D_6): δ –92.0 (mc, 2F, *ortho*-F), –140.3 (mc, 2F, *meta*-F).

The NMR analysis is in agreement with that reported in the literature.[11]

4-methylenetetrafluoropyridine-substituted phosphorane (8):

^{1}H NMR (400 MHz, C_6D_6): δ 1.34 (dt, $^{2}J_{HP}$ = 17.3 Hz, $^{3}J_{HF}$ = 12.5 Hz, 6H, C*H₃*), 3.09 (dtt, $^{2}J_{HP}$ = 23.5 Hz, $^{3}J_{HF}$ = 5.0 Hz, $^{4}J_{HF}$ = 1.8 Hz, 2H, CH_2)**.**

^{19}F NMR (377 MHz, C_6D_6): δ –9.7 (dm, $^{1}J_{PF}$ = 595.1 Hz, 2F, *F*₂P), –92.6 (mc, 2F, *ortho-F*), –144.5 (mc, 2F, *meta-F)*.

^{31}P NMR (162 MHz, C_6D_6): δ –20.5 (tm, J_{PF} = 595.0 Hz, *P*F_2).

^{3}C{^{1}H} NMR (101 MHz, C_6D_6): δ 142.9 – 141.5 (m, 2C, *C*F), 140.1 – 138.7 (m, 2C, *C*F), 129.5 – 128.4 (m, 1C, *C*(sp^{2})–CH_2), 27.9 (dt, $^{1}J_{CP}$ = 123.2 Hz, $^{2}J_{CF}$ = 32.9 Hz, 1C, *C*H_2), 18.7 (dt, $^{1}J_{CP}$ = 127.2 Hz, $^{2}J_{CF}$ = 28.9 Hz, 2C, *C*H_3).

GC(EI)-MS: $[C_8H_8F_6NP]^{\bullet+}$ 263.02817, $[C_8H_8F_6NP]^{\bullet+}$ 248.00528, $[C_6H_2F_4NP]^{\bullet+}$ 164.01269, $[C_2H_6F_2P]^{\bullet+}$ 99.01827 *m/z*.

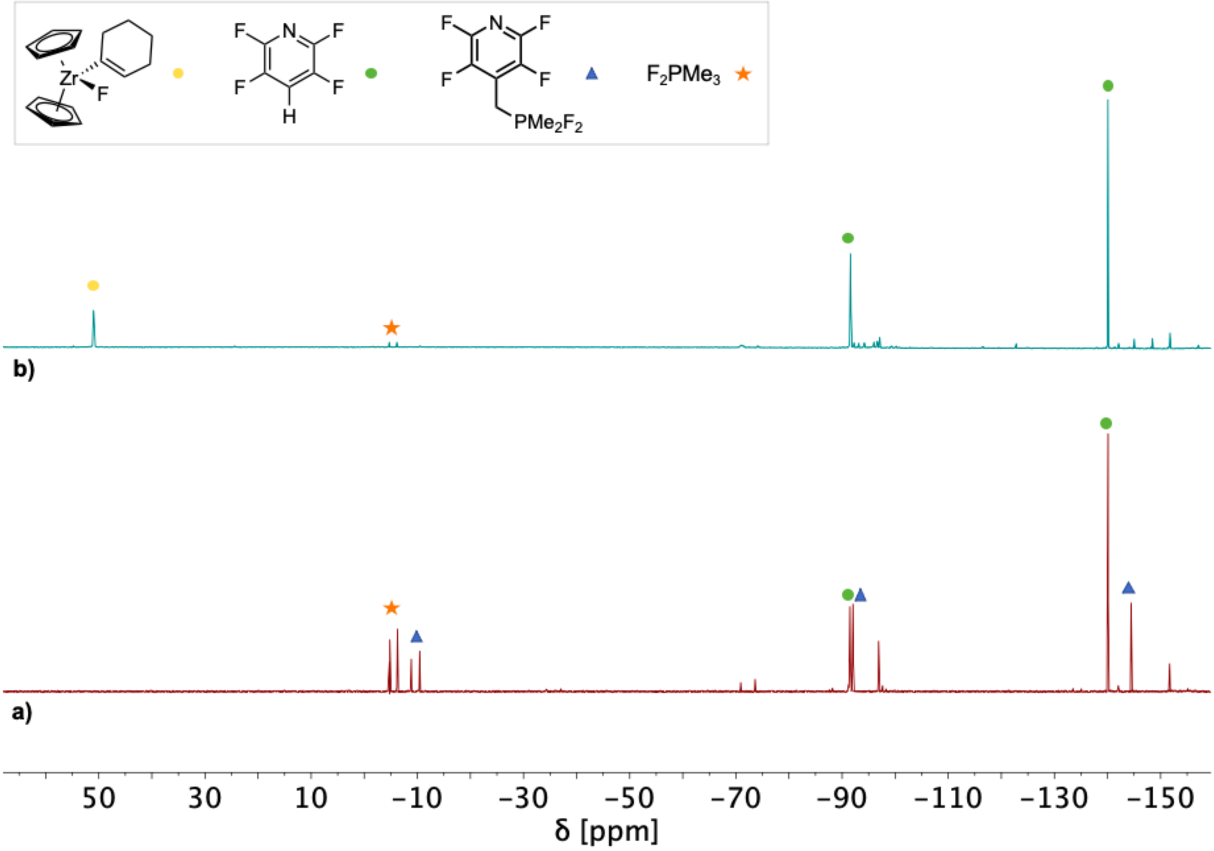

Figure S15 ^{19}F NMR (377 MHz, C_6D_6) spectra of the crude reaction mixture between PMe_3 and pentafluoropyridine (1:1, in C_6D_6) (a) and after 3 hours from the addition of complex **1** (b).

2 X-ray crystal structure

2.1 Molecular structure of 3

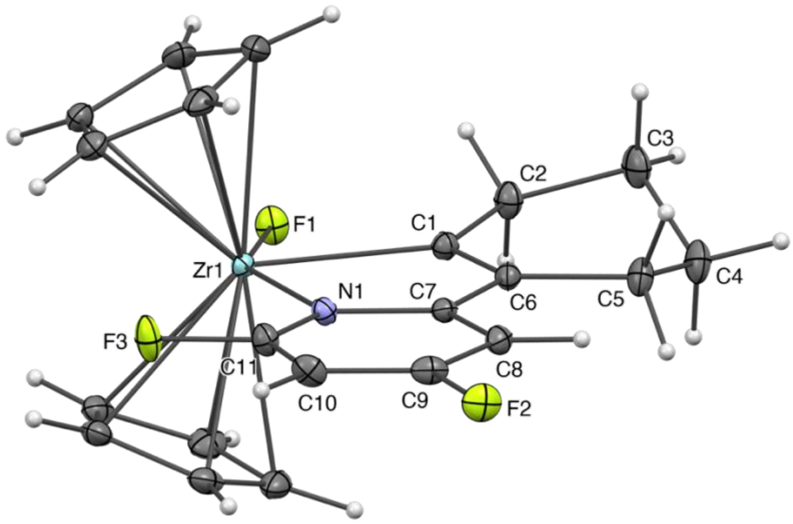

X-ray molecular structure of complex 3

**Crystal data**	
Chemical formula	$C_{21}H_{20}F_3NZr$
M_r	434.60
Crystal system, space group	Monoclinic, $P2_1/n$
Temperature (K)	110
a, *b*, *c* (Å)	8.1766 (1), 15.5546 (2), 14.6671 (2)
β (°)	99.429 (1)
V (Å^3)	1840.21 (4)
Z	4
Radiation type	Cu Kα
μ (mm^{-1})	5.19
Crystal size (mm)	0.25 × 0.1 × 0.09
**Data collection**	
Diffractometer	SuperNova
Absorption correction	Gaussian
T_{min}, T_{max}	0.760, 1.000
No. of measured, independent and observed [I > 2σ(I)] reflections	12546, 3722, 3429
R_{int}	0.038
(sin θ/λ)$_{max}$ (Å^{-1})	0.632
**Refinement**	
$R[F^2 > 2\sigma(F^2)]$, $wR(F^2)$, S	0.025, 0.062, 1.10
No. of reflection	3722
No. of parameters	235
H-atom treatment	All H-atom parameters refined
$\Delta\rho_{max}$, $\Delta\rho_{min}$ (e Å^{-3})	0.32, −0.47

2.2 Structural data comparison

Compound	Reference	Zr–C	Zr–N	Cα-Cβ	Cβ-Cδ	Cδ-N	N/Cα-Zr-X	N-Zr-Cα	Zr-Cα-Cβ	Cα-Cβ-Cδ	Cβ-Cδ-N	Cδ-N-Zr
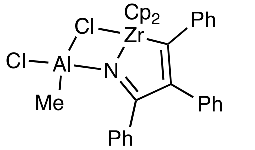	Binger *et al.* *Organometallics* **1995**, *14*, 2969–2976. https://doi.org/10.1021/om00006a047.	2.363(3)	2.228(2)	1.359(4)	1.479(4)	1.285(3)	140.7(1)	71.9(1)	113.6(2)	116.1(2)	118.9(2)	119.5(2)
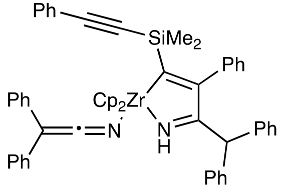	Zhao *et al.* *Organometallics* **2012**, *31*, 8370–8374. https://doi.org/10.1021/om300949a.	2.403(2)	2.2629(18)	1.360(3)	1.462(4)	1.288(3)	140.52(7)	69.04(7)	114.6(2)	117.0(2)	116.0(2)	123.1(2)
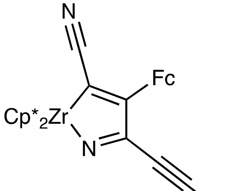 Fc = ferrocenyl	Becker *et al. Chem. Eur. J.* **2014**, *20*, 3061–3068. https://doi.org/10.1002/chem.201304478.	2.4445(15)	2.1256(12)	1.370(2)	1.506(2)	1.273(2)	142.76(5)	69.90(5)	114.02(10)	111.30(13)	118.91(13)	125.08(10)
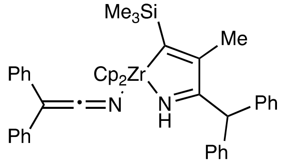	Zhao *et al.* *Organometallics* **2011**, *30*, 3464–3467. https://doi.org/10.1021/om200404p.	2.407(4)	2.265(4)	1.361(8)	1.473(6)	1.289(6)	139.8(1)	68.4(1)	115.5(3)	116.2(4)	115.5(4)	123.4(3)
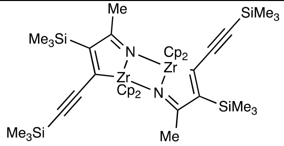	Burlakov *et al. Eur. J. Inorg. Chem.* **2014**, 5304–5310. https://doi.org/10.1002/ejic.201402618.	2.3640(15)	2.2630(12)<br>2.3384(12)	1.361(2)	1.487(2)	1.289(2)	140.49(5)	70.28(5)	117.6(1)	112.1(1)	120.8(1)	119.1(1)

Table S3 Structural data of 2-aza-zirconacyclopentadienes.

		Zr–C	Zr–N	Cα-Cβ	Cβ-Cδ	Cδ-N	Cα-Zr-X	N-Zr-Cα	Zr-Cα-Cβ	Cα-Cβ-Cδ	Cβ-Cδ-N	Cδ-N-Zr
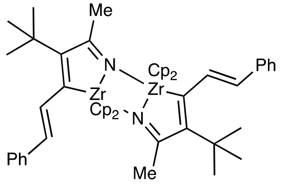	Benderet *al. Dalton Trans.* **2013**, *42*, 14673–14676. https://doi.org/10.1039/C3DT51497H.	2.402(7)	2.247(5)	1.347(9)	1.498(9)	1.286(8)	141.7(2)	71.0(2)	114.3(5)	112.2(5)	120.4(4)	118.6(4)
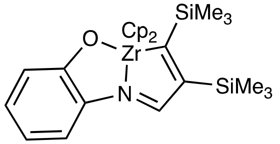	Arndtet *al. Chem. Ber.* **1996**, *129*, 207–211. https://doi.org/10.1002/cber.19961290214.	2.426(3)	2.301(3)	1.373(5)	1.458(4)	1.287(4)	139.0(1)	69.2(10)	115.7(2)	114.3(3)	120.9(3)	119.8(2)
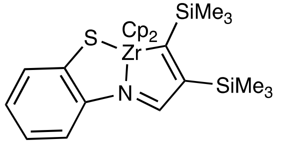	Arndt *et al. Chem. Ber.* **1996**, *129*, 207–211. https://doi.org/10.1002/cber.19961290214.	2.405(11)	2.330(8)	1.380(15)	1.477(15)	1.293(13)	134.6(1)	69.7(17)	114.8(7)	113.9(4)	121.6(5)	116.8(4)
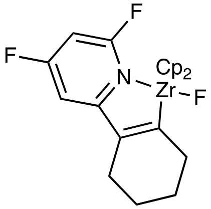	This work	2.321(2)	2.479(2)	1.354(3)	1.456(3)	1.372(3)	148.46(6)	68.34(7)	121.15(15)	117.92(18)	116.37(18)	115.83(13)

Table S3 (continued).

3 DFT calculations

3.1 General Methods

DFT calculations were performed using the TURBOMOLE V7.8.1 package using the resolution of identity (RI) approximation.[12-19] Initial optimisations were performed at the (RI-)BP86/SV(P) level with an m5 grid, followed by frequency calculations at the same level. All minima were confirmed as such by the absence of imaginary frequencies, transitions states had a single imaginary frequency. Single-point energies were then performed on the (RI-)BP86/SV(P) optimised geometries using the hybrid PBE0 functional and the flexible def2-TZVPP basis set. Energies, xyz coordinates and the first 50 lines of the vibrational spectra are presented. Solvation effects were modelled using COMSO[20] using the dielectronic constant of 2.27 for benzene, 2.02 for cyclohexane and 8.73 for CH_2Cl_2. Wnergies were corrected for dispersion using Grimme's D3-method[21] with Becke-Johnson dampening.[22]

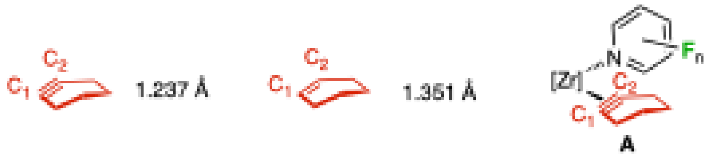

Py ligand	Zr….N / Å	Zr….C_1 / Å	Zr….C_2 / Å	C_1….C_2 / Å
**None**	N/A	2.163	2.163	1.356
**H**	2.483	2.190	2.235	1.377
**F1**-1	2.529	2.192	2.228	1.334
**F1**-2	2.541	2.186	2.243	1.338
**F2**	2.657	2.184	2.225	1.336
**F3**	2.667	2.182	2.224	1.337
**F4**	2.683	2.183	2.226	1.336
**F5**	2.704	2.181	2.223	1.337

Table S4. Comparison of the calculated bond metrics in **A**, cyclohexyne and the 16-electron complex [Cp_2Zr(*c*-$C_2(CH_2)_4$)]. [Zr] = Cp_2Zr.

3.2 Collated Energies and xyz coordinates

1

```
SCF Energy (au) BP86/SV(P)                -1128.167390448
SCF Energy (au) PBE0/def2-TZVPP           -1127.990376521
SCF Energy (au) PBE0/def2-TZVPP           -1127.9998918961 (CH2Cl2 Correction)
SCF Energy (au) PBE0/def2-TZVPP           -1127.9951772827 (C6H6 Correction)
SCF Energy (au) PBE0/def2-TZVPP           -1127.9945725827 (C6H12 Correction)
Zero Point Energy (au)                    0.3939095
Chemical Potential (kJ mol-1)             895.22
Dispersion Correction (au) PBE0/def2-TZVPP -0.06691185
```

xyz coordinates
48

Zr	-0.6594056	-0.7788597	0.1692119
P	-0.4515437	1.6396458	1.3827544
C	1.0795613	0.2229149	-0.8926745
C	-1.1903908	-2.2488964	2.2646393
H	-2.2127604	-2.2726603	2.6649090
C	2.1467944	1.1649024	-1.4026093
H	2.6625284	1.7257289	-0.5851147
H	1.6754134	1.9562295	-2.0393372
C	-0.6563452	-3.1130453	1.2607690
H	-1.1920730	-3.9328837	0.7632152
C	0.8826929	-0.9967777	-1.3898626
C	0.7157226	-2.7673140	1.0555692
H	1.4118597	-3.2675796	0.3711803
C	-2.9225029	0.4108278	-0.3826910
H	-3.2311792	1.3384358	0.1187425
C	-0.1538515	-1.3577768	2.6668224
H	-0.2333886	-0.6052977	3.4634778
C	1.6635838	-1.6443058	-2.4996357
H	0.9995634	-2.1708229	-3.2267342
H	2.3152480	-2.4482200	-2.0745343
C	-2.7376923	-1.8199128	-0.9308742
H	-2.8506921	-2.9124796	-0.9158059
C	3.1960557	0.3919073	-2.2427374
H	3.8536575	1.1054271	-2.7911853
H	3.8581796	-0.1830240	-1.5521776
C	1.0256147	-1.6762968	1.9191500
H	2.0006858	-1.1782655	1.9984790
C	-3.2638874	-0.9091959	0.0347122
H	-3.8415746	-1.1733296	0.9305363
C	-2.1928483	0.3180787	-1.6098789
H	-1.7901612	1.1573098	-2.1917808
C	2.5328419	-0.5883510	-3.2307669
H	3.3075286	-1.0883611	-3.8565154
H	1.8864520	-0.0085326	-3.9319705
C	-2.0821459	-1.0620773	-1.9514546
H	-1.6009022	-1.4686284	-2.8497349
C	-1.6345934	2.1564370	2.7371478
C	-0.5712064	3.1101877	0.2561847
C	1.1744544	1.9582445	2.2204848
H	1.3140941	1.2490761	3.0632938
H	1.2250652	2.9981442	2.6132703
H	1.9934627	1.7963480	1.4901474
H	0.2042837	3.0231544	-0.5318400
H	-0.4272227	4.0589641	0.8196006
H	-1.5661035	3.1318602	-0.2365713
H	-1.3902223	3.1660789	3.1370857
H	-1.5987241	1.4228327	3.5711256
H	-2.6739270	2.1701588	2.3439779

vibrational spectrum

# mode	symmetry	wave number cm**(-1)	IR intensity km/mol	selection rules IR	RAMAN
1		-0.00	0.00000	-	-
2		-0.00	0.00000	-	-
3		-0.00	0.00000	-	-
4		0.00	0.00000	-	-
5		0.00	0.00000	-	-
6		0.00	0.00000	-	-
7	a	32.16	0.00186	YES	YES
8	a	51.49	0.06175	YES	YES
9	a	54.66	0.01211	YES	YES
10	a	60.85	0.18411	YES	YES
11	a	76.64	1.24496	YES	YES
12	a	87.11	0.34542	YES	YES
13	a	95.89	0.31977	YES	YES
14	a	112.67	0.09166	YES	YES
15	a	127.05	0.49329	YES	YES
16	a	143.02	0.16479	YES	YES
17	a	155.93	0.56909	YES	YES
18	a	157.00	1.43085	YES	YES
19	a	162.23	0.80148	YES	YES
20	a	167.34	1.02352	YES	YES
21	a	191.62	0.28581	YES	YES
22	a	196.46	0.76940	YES	YES
23	a	219.67	0.11922	YES	YES
24	a	226.40	3.20054	YES	YES
25	a	240.77	0.97688	YES	YES
26	a	248.68	5.01112	YES	YES
27	a	257.51	0.17636	YES	YES
28	a	259.23	0.36431	YES	YES
29	a	262.49	0.79313	YES	YES
30	a	264.63	0.72482	YES	YES
31	a	279.01	1.12255	YES	YES
32	a	293.51	6.56410	YES	YES
33	a	308.32	8.99486	YES	YES
34	a	314.18	19.28485	YES	YES
35	a	335.12	1.51767	YES	YES
36	a	410.52	1.23255	YES	YES
37	a	460.06	0.35257	YES	YES
38	a	523.57	0.71442	YES	YES
39	a	593.95	0.89015	YES	YES
40	a	595.88	0.25218	YES	YES
41	a	597.93	0.58287	YES	YES
42	a	602.36	0.29627	YES	YES
43	a	633.51	12.38616	YES	YES
44	a	681.26	4.89965	YES	YES
45	a	685.14	8.08745	YES	YES
46	a	699.85	10.14127	YES	YES
47	a	759.55	1.92634	YES	YES
48	a	766.73	91.86585	YES	YES
49	a	767.65	119.01580	YES	YES
50	a	773.74	5.98914	YES	YES

PMe_3

SCF Energy (au) BP86/SV(P)	-460.9233674584	
SCF Energy (au) PBE0/def2-TZVPP	-460.8709757608	
SCF Energy (au) PBE0/def2-TZVPP	-460.8752962778	(CH_2Cl_2 Correction)
SCF Energy (au) PBE0/def2-TZVPP	-460.8731205431	(C_6H_6 Correction)
SCF Energy (au) PBE0/def2-TZVPP	-460.8728469920	(C_6H_{12} Correction)
Zero Point Energy (au)	0.1088853	
Chemical Potential (kJ mol^{-1})	208.57	
Dispersion Correction (au) PBE0/def2-TZVPP	-0.00873543	

xyz coordinates
13

P	-0.1295231	-0.8491666	-0.3814261
C	-1.2953682	-0.2934559	0.9740931
C	-0.2275767	0.6533155	-1.4943974
C	1.5030996	-0.4900856	0.4620205
H	1.6618544	-1.2191516	1.2870966
H	1.5575815	0.5415028	0.8807053
H	2.3333018	-0.6242114	-0.2661696
H	0.5329786	0.5661087	-2.3015266
H	-0.0607135	1.6102618	-0.9476877
H	-1.2274865	0.6895742	-1.9805556
H	-1.0579758	0.7248975	1.3601597
H	-1.2515194	-1.0149393	1.8196597
H	-2.3386525	-0.2946501	0.5880280

vibrational spectrum

# mode	symmetry	wave number	IR intensity	selection rules	
#		cm**(-1)	km/mol	IR	RAMAN
1		-0.00	0.00000	-	-
2		0.00	0.00000	-	-
3		0.00	0.00000	-	-
4		0.00	0.00000	-	-
5		0.00	0.00000	-	-
6		0.00	0.00000	-	-
7	a	178.80	0.00001	YES	YES
8	a	201.72	0.05688	YES	YES
9	a	201.93	0.05649	YES	YES
10	a	242.08	0.12910	YES	YES
11	a	242.28	0.13000	YES	YES
12	a	284.56	0.19815	YES	YES
13	a	625.80	0.02293	YES	YES
14	a	679.37	12.43552	YES	YES
15	a	679.44	12.44542	YES	YES
16	a	768.76	0.00003	YES	YES
17	a	818.43	1.29603	YES	YES
18	a	818.72	1.29925	YES	YES
19	a	932.10	24.43917	YES	YES
20	a	932.22	24.42923	YES	YES
21	a	950.39	31.16371	YES	YES
22	a	1261.44	3.75348	YES	YES
23	a	1261.56	3.74639	YES	YES
24	a	1287.32	4.63875	YES	YES
25	a	1397.19	0.00020	YES	YES
26	a	1403.11	7.49281	YES	YES
27	a	1403.19	7.49826	YES	YES
28	a	1413.00	10.82005	YES	YES
29	a	1413.04	10.80722	YES	YES
30	a	1421.72	13.59568	YES	YES
31	a	2927.17	28.15530	YES	YES
32	a	2929.03	18.01089	YES	YES
33	a	2929.10	18.04425	YES	YES

34	a	3022.85	32.75955	YES	YES
35	a	3023.95	1.75715	YES	YES
36	a	3024.05	1.66023	YES	YES
37	a	3040.17	9.37954	YES	YES
38	a	3040.24	8.30494	YES	YES
39	a	3040.39	1.43075	YES	YES

Py-H

SCF Energy (au) BP86/SV(P)	-248.1066180477
SCF Energy (au) PBE0/def2-TZVPP	-248.0746583058
SCF Energy (au) PBE0/def2-TZVPP	-248.0817490489 (CH_2Cl_2 Correction)
SCF Energy (au) PBE0/def2-TZVPP	-248.0781857274 (C6H6 Correction)
SCF Energy (au) PBE0/def2-TZVPP	-248.0777356766 (C6H12 Correction)
Zero Point Energy (au)	0.0861996
Chemical Potential (kJ mol^{-1})	155.79
Dispersion Correction (au) PBE0/def2-TZVPP	-0.00841143

xyz coordinates
11

H	0.0000000	0.0000000	2.3069622
H	2.0810168	0.0000000	-1.5155506
N	0.0000000	0.0000000	-1.6187902
C	0.0000000	0.0000000	1.2038472
C	-1.2067847	0.0000000	0.4857513
H	-2.1800994	0.0000000	1.0037004
C	1.1480564	0.0000000	-0.9199064
C	1.2067847	0.0000000	0.4857513
H	2.1800994	0.0000000	1.0037004
C	-1.1480564	0.0000000	-0.9199064
H	-2.0810168	0.0000000	-1.5155506

vibrational spectrum

# mode	symmetry	wave number	IR intensity	selection rules	
#		cm**(-1)	km/mol	IR	RAMAN
1		-0.00	0.00000	-	-
2		-0.00	0.00000	-	-
3		-0.00	0.00000	-	-
4		0.00	0.00000	-	-
5		0.00	0.00000	-	-
6		0.00	0.00000	-	-
7	a2	361.88	0.00000	NO	YES
8	b2	407.45	2.95433	YES	YES
9	a1	590.52	4.10983	YES	YES
10	b1	649.73	0.36894	YES	YES
11	b2	695.46	51.02019	YES	YES
12	b2	743.76	4.40544	YES	YES
13	a2	870.30	0.00000	NO	YES
14	b2	923.19	0.01559	YES	YES
15	a2	964.45	0.00000	NO	YES
16	a1	981.06	8.96027	YES	YES
17	b2	984.79	0.00078	YES	YES
18	a1	1021.17	3.12093	YES	YES
19	b1	1049.26	0.00011	YES	YES
20	a1	1062.16	4.76690	YES	YES
21	b1	1129.74	1.35033	YES	YES
22	a1	1206.08	3.22796	YES	YES
23	b1	1324.56	0.83578	YES	YES
24	b1	1338.32	0.09058	YES	YES
25	b1	1436.45	23.90803	YES	YES
26	a1	1471.11	2.85488	YES	YES
27	a1	1592.10	20.15549	YES	YES
28	b1	1593.46	10.48642	YES	YES
29	b1	3059.88	37.24818	YES	YES
30	a1	3063.13	9.44161	YES	YES
31	a1	3093.75	3.78886	YES	YES
32	b1	3107.83	24.98731	YES	YES
33	a1	3115.63	7.55444	YES	YES

Py-F$_1$

SCF Energy (au) BP86/SV(P) -347.2852714218
SCF Energy (au) PBE0/def2-TZVPP -347.2766268916
SCF Energy (au) PBE0/def2-TZVPP -347.2837403032 (CH_2Cl_2 Correction)
SCF Energy (au) PBE0/def2-TZVPP -347.2801537587 (C_6H_6 Correction)
SCF Energy (au) PBE0/def2-TZVPP -347.2797027048 (C_6H_{12} Correction)
Zero Point Energy (au) 0.0785671
Chemical Potential (kJ mol^{-1}) 130.46
Dispersion Correction (au) PBE0/def2-TZVPP -0.00858195

xyz coordinates
11

H	1.6015831	1.6671717	0.0000000
F	-2.7412643	0.3847904	0.0000000
N	-1.0933177	-1.1679051	0.0000000
C	0.8446757	0.8653438	0.0000000
C	1.2301077	-0.4884862	0.0000000
H	2.2916668	-0.7818855	0.0000000
C	-1.4252943	0.1107581	0.0000000
C	-0.5190429	1.1872027	0.0000000
H	-0.8838022	2.2257080	0.0000000
C	0.2199891	-1.4632811	0.0000000
H	0.4746990	-2.5394167	0.0000000

vibrational spectrum

# mode	symmetry	wave number	IR intensity	selection rules	
#		cm**(-1)	km/mol	IR	RAMAN
1		-0.00	0.00000	-	-
2		-0.00	0.00000	-	-
3		-0.00	0.00000	-	-
4		-0.00	0.00000	-	-
5		-0.00	0.00000	-	-
6		0.00	0.00000	-	-
7	a"	209.03	0.01200	YES	YES
8	a"	413.41	2.90297	YES	YES
9	a'	426.27	0.28405	YES	YES
10	a"	511.44	2.73962	YES	YES
11	a'	545.07	3.62260	YES	YES
12	a'	610.18	2.82553	YES	YES
13	a"	724.81	4.23074	YES	YES
14	a"	768.69	52.18757	YES	YES
15	a'	834.64	23.28594	YES	YES
16	a"	859.36	0.75921	YES	YES
17	a"	942.00	0.32823	YES	YES
18	a"	971.77	0.00316	YES	YES
19	a'	978.51	8.02664	YES	YES
20	a'	1037.20	5.29255	YES	YES
21	a'	1082.34	2.34048	YES	YES
22	a'	1124.41	3.33035	YES	YES
23	a'	1260.74	79.06099	YES	YES
24	a'	1290.47	16.66498	YES	YES
25	a'	1352.59	1.54731	YES	YES
26	a'	1430.02	53.93658	YES	YES
27	a'	1472.95	100.34199	YES	YES
28	a'	1601.36	47.24652	YES	YES
29	a'	1602.36	95.15052	YES	YES
30	a'	3075.35	17.53401	YES	YES
31	a'	3105.25	5.02985	YES	YES
32	a'	3124.49	10.16457	YES	YES
33	a'	3132.26	1.46046	YES	YES

Py-F_2

SCF Energy (au) BP86/SV(P)	-446.4628101159
SCF Energy (au) PBE0/def2-TZVPP	-446.4771630783
SCF Energy (au) PBE0/def2-TZVPP	-446.4838649318 (CH_2Cl_2 Correction)
SCF Energy (au) PBE0/def2-TZVPP	-446.4804778812 (C_6H_6 Correction)
SCF Energy (au) PBE0/def2-TZVPP	-446.4800533256 (C_6H_{12} Correction)
Zero Point Energy (au)	0.0708950
Chemical Potential (kJ mol^{-1})	106.90
Dispersion Correction (au) PBE0/def2-TZVPP	-0.00875519

xyz coordinates
11

H	1.5982045	1.6769295	0.0000000
F	-2.7466546	0.3948000	0.0000000
N	-1.0912165	-1.1449385	0.0000000
C	0.8378088	0.8790529	0.0000000
C	1.2326367	-0.4685096	0.0000000
H	2.2873489	-0.7795440	0.0000000
C	-1.4321783	0.1348224	0.0000000
C	-0.5271592	1.2087138	0.0000000
H	-0.8884399	2.2472946	0.0000000
C	0.2034383	-1.4240704	0.0000000
F	0.5262114	-2.7245507	0.0000000

vibrational spectrum

# mode	symmetry	wave number	IR intensity	selection rules	
#		cm**(-1)	km/mol	IR	RAMAN
1		-0.00	0.00000	-	-
2		-0.00	0.00000	-	-
3		-0.00	0.00000	-	-
4		0.00	0.00000	-	-
5		0.00	0.00000	-	-
6		0.00	0.00000	-	-
7	a"	199.78	0.00000	YES	YES
8	a"	237.96	0.33349	YES	YES
9	a'	345.96	0.91950	YES	YES
10	a"	453.51	0.14163	YES	YES
11	a'	492.42	2.15477	YES	YES
12	a'	529.67	0.26889	YES	YES
13	a'	561.02	3.62844	YES	YES
14	a"	658.93	0.00000	YES	YES
15	a"	707.88	2.80961	YES	YES
16	a'	731.19	11.63430	YES	YES
17	a"	775.70	64.24960	YES	YES
18	a"	853.70	0.00000	YES	YES
19	a"	959.75	0.00110	YES	YES
20	a'	978.99	5.98344	YES	YES
21	a'	999.44	71.47849	YES	YES
22	a'	1056.10	4.72416	YES	YES
23	a'	1122.97	1.92542	YES	YES
24	a'	1227.85	77.20296	YES	YES
25	a'	1314.07	84.45845	YES	YES
26	a'	1373.50	1.67788	YES	YES
27	a'	1453.87	242.62392	YES	YES
28	a'	1457.49	12.11769	YES	YES
29	a'	1611.77	151.42853	YES	YES
30	a'	1612.12	166.70661	YES	YES
31	a'	3114.91	3.87649	YES	YES
32	a'	3143.04	1.50392	YES	YES
33	a'	3145.68	0.00015	YES	YES

Py-F_3

SCF Energy (au) BP86/SV(P)	-545.6316602660	
SCF Energy (au) PBE0/def2-TZVPP	-545.6690681160	
SCF Energy (au) PBE0/def2-TZVPP	-545.6749196402	(CH_2Cl_2 Correction)
SCF Energy (au) PBE0/def2-TZVPP	-545.6720184625	(C_6H_6 Correction)
SCF Energy (au) PBE0/def2-TZVPP	-545.6716465148	(C_6H_{12} Correction)
Zero Point Energy (au)	0.0630242	
Chemical Potential (kJ mol^{-1})	82.85	
Dispersion Correction (au) PBE0/def2-TZVPP	-0.00893316	

xyz coordinates
11

F	1.7319821	1.8179280	0.0000000
F	-2.7581120	0.3813530	0.0000000
N	-1.1079082	-1.1629839	0.0000000
C	0.8097940	0.8500300	0.0000000
C	1.2209815	-0.4904347	0.0000000
H	2.2819489	-0.7750660	0.0000000
C	-1.4469321	0.1178617	0.0000000
C	-0.5489766	1.1957866	0.0000000
H	-0.8851221	2.2415716	0.0000000
C	0.1878784	-1.4395630	0.0000000
F	0.5144662	-2.7364835	0.0000000

vibrational spectrum

# mode	symmetry	wave number	IR intensity	selection rules	
#		cm**(-1)	km/mol	IR	RAMAN
1		-0.00	0.00000	-	-
2		-0.00	0.00000	-	-
3		0.00	0.00000	-	-
4		0.00	0.00000	-	-
5		0.00	0.00000	-	-
6		0.00	0.00000	-	-
7	a"	203.42	0.45652	YES	YES
8	a"	203.47	1.01142	YES	YES
9	a"	255.66	1.96906	YES	YES
10	a'	331.10	1.55840	YES	YES
11	a'	343.32	0.57144	YES	YES
12	a'	491.86	1.24391	YES	YES
13	a'	509.76	7.48678	YES	YES
14	a'	585.65	0.00955	YES	YES
15	a'	603.44	1.15471	YES	YES
16	a"	623.07	5.21582	YES	YES
17	a"	648.62	0.00000	YES	YES
18	a"	704.96	0.63080	YES	YES
19	a"	807.91	0.00047	YES	YES
20	a"	810.22	63.84357	YES	YES
21	a'	974.70	33.19410	YES	YES
22	a'	992.19	12.91264	YES	YES
23	a'	1043.83	63.44464	YES	YES
24	a'	1131.26	130.53130	YES	YES
25	a'	1150.54	62.28289	YES	YES
26	a'	1374.92	12.34158	YES	YES
27	a'	1405.15	3.35231	YES	YES
28	a'	1438.36	189.63072	YES	YES
29	a'	1471.02	34.86740	YES	YES
30	a'	1619.66	357.35623	YES	YES
31	a'	1620.48	266.49670	YES	YES
32	a'	3159.67	0.88350	YES	YES
33	a'	3160.18	2.72383	YES	YES

Py-F_4

SCF Energy (au) BP86/SV(P)	-644.7837304238	
SCF Energy (au) PBE0/def2-TZVPP	-644.8421339934	
SCF Energy (au) PBE0/def2-TZVPP	-644.8476718043	(CH_2Cl_2 Correction)
SCF Energy (au) PBE0/def2-TZVPP	-644.8449326172	(C_6H_6 Correction)
SCF Energy (au) PBE0/def2-TZVPP	-644.8445805735	(C_6H_{12} Correction)
Zero Point Energy (au)	0.0550248	
Chemical Potential (kJ mol^{-1})	58.42	
Dispersion Correction (au) PBE0/def2-TZVPP	-0.00914508	

xyz coordinates
11

H	1.5867480	1.6648190	0.0000000
F	-2.7724589	0.3904667	0.0000000
N	-1.0945470	-1.1483529	0.0000000
C	0.8275832	0.8682382	0.0000000
C	1.2044832	-0.4805405	0.0000000
F	2.4943293	-0.8359928	0.0000000
C	-1.4650272	0.1181661	0.0000000
C	-0.5377388	1.1800528	0.0000000
F	-0.9545322	2.4514260	0.0000000
C	0.1882664	-1.4577063	0.0000000
F	0.5228941	-2.7505762	0.0000000

vibrational spectrum

# mode	symmetry	wave number	IR intensity	selection rules	
#		cm**(-1)	km/mol	IR	RAMAN
1		-0.00	0.00000	-	-
2		-0.00	0.00000	-	-
3		-0.00	0.00000	-	-
4		-0.00	0.00000	-	-
5		-0.00	0.00000	-	-
6		0.00	0.00000	-	-
7	a"	109.08	0.00000	YES	YES
8	a"	197.82	1.76578	YES	YES
9	a'	270.27	0.59169	YES	YES
10	a"	284.70	0.72571	YES	YES
11	a'	292.68	0.10008	YES	YES
12	a'	353.48	1.42726	YES	YES
13	a'	438.57	0.04675	YES	YES
14	a"	453.09	0.00001	YES	YES
15	a"	465.17	0.70606	YES	YES
16	a'	492.02	1.90054	YES	YES
17	a'	649.86	1.82903	YES	YES
18	a"	662.99	0.00022	YES	YES
19	a"	666.52	3.06116	YES	YES
20	a'	666.88	10.99359	YES	YES
21	a'	735.08	10.04295	YES	YES
22	a"	854.18	31.08116	YES	YES
23	a'	897.99	99.10693	YES	YES
24	a'	1148.14	49.62065	YES	YES
25	a'	1176.31	17.52809	YES	YES
26	a'	1215.51	175.44777	YES	YES
27	a'	1365.03	89.61741	YES	YES
28	a'	1408.79	0.31596	YES	YES
29	a'	1446.72	45.86739	YES	YES
30	a'	1504.35	639.55033	YES	YES
31	a'	1615.46	21.72106	YES	YES
32	a'	1636.18	2.59031	YES	YES
33	a'	3146.21	2.71978	YES	YES

Py-F$_5$

SCF Energy (au) BP86/SV(P)	-743.9396048525	
SCF Energy (au) PBE0/def2-TZVPP	-744.0210814730	
SCF Energy (au) PBE0/def2-TZVPP	-744.0253403874	(CH_2Cl_2 Correction)
SCF Energy (au) PBE0/def2-TZVPP	-744.0232713855	(C_6H_6 Correction)
SCF Energy (au) PBE0/def2-TZVPP	-744.0229997997	(C_6H_{12} Correction)
Zero Point Energy (au)	0.0472154	
Chemical Potential (kJ mol^{-1})	34.50	
Dispersion Correction (au) PBE0/def2-TZVPP	-0.00935967	

xyz coordinates
11

F	1.7223749	1.8074406	0.0000000
F	-2.7853753	0.3751348	0.0000000
N	-1.1099855	-1.1645672	0.0000000
C	0.8057538	0.8453169	0.0000000
C	1.1971547	-0.5074652	0.0000000
F	2.4893803	-0.8394770	0.0000000
C	-1.4793167	0.1027306	0.0000000
C	-0.5643298	1.1713734	0.0000000
F	-0.9575199	2.4463361	0.0000000
C	0.1735399	-1.4726670	0.0000000
F	0.5083235	-2.7641560	0.0000000

vibrational spectrum

# mode	symmetry	wave number cm**(-1)	IR intensity km/mol	selection rules IR	RAMAN
1		-0.00	0.00000	-	-
2		0.00	0.00000	-	-
3		0.00	0.00000	-	-
4		0.00	0.00000	-	-
5		0.00	0.00000	-	-
6		0.00	0.00000	-	-
7	a"	111.15	0.00000	YES	YES
8	a"	146.75	0.01523	YES	YES
9	a"	207.10	2.27357	YES	YES
10	a'	257.88	0.04018	YES	YES
11	a'	259.88	0.66512	YES	YES
12	a'	309.52	0.46268	YES	YES
13	a'	337.46	0.73626	YES	YES
14	a"	339.35	2.36019	YES	YES
15	a"	425.40	0.00000	YES	YES
16	a'	443.22	0.03265	YES	YES
17	a'	459.54	0.39352	YES	YES
18	a"	577.85	0.48023	YES	YES
19	a'	579.24	0.00034	YES	YES
20	a"	637.19	3.63495	YES	YES
21	a"	641.71	0.00002	YES	YES
22	a'	675.39	3.36504	YES	YES
23	a'	726.07	0.05131	YES	YES
24	a'	977.62	175.73986	YES	YES
25	a'	1076.28	210.10705	YES	YES
26	a'	1172.34	0.05155	YES	YES
27	a'	1286.19	24.38580	YES	YES
28	a'	1372.19	23.29711	YES	YES
29	a'	1431.36	21.52063	YES	YES
30	a'	1496.13	631.66017	YES	YES
31	a'	1522.53	139.08657	YES	YES
32	a'	1618.04	106.93652	YES	YES
33	a'	1637.79	4.53080	YES	YES

A-H

SCF Energy (au) BP86/SV(P) -915.3442775939
SCF Energy (au) PBE0/def2-TZVPP -915.1832815574
SCF Energy (au) PBE0/def2-TZVPP -915.1956850082 (CH_2Cl_2 Correction)
SCF Energy (au) PBE0/def2-TZVPP -915.1895365135 (C_6H_6 Correction)
SCF Energy (au) PBE0/def2-TZVPP -915.1887477033 (C_6H_{12} Correction)
Zero Point Energy (au) 0.3708127
Chemical Potential (kJ mol^{-1}) 835.05
Dispersion Correction (au) PBE0/def2-TZVPP -0.06217159

xyz coordinates
46

Zr	-0.5365648	-0.6991669	0.2113524
H	-0.5063896	4.9180766	3.2862371
H	-1.4237340	0.6707292	3.0980209
N	-0.4309775	1.4557629	1.4395755
C	-0.4877173	3.9460558	2.7661457
C	0.0820644	3.8213188	1.4910682
H	0.5240447	4.6871220	0.9733743
C	-0.9894870	1.5863648	2.6655938
C	-1.0353216	2.7991564	3.3649279
H	-1.4988614	2.8375072	4.3633079
C	1.0474155	0.2155466	-1.0731919
C	-0.5931482	-1.9192015	2.5191726
H	-1.4071767	-1.7257785	3.2321848
C	2.1353511	1.0794787	-1.6767551
H	2.7673866	1.5684839	-0.8954279
H	1.6736662	1.9253258	-2.2480333
C	0.0951944	2.5628501	0.8685605
C	-0.5965016	-2.9201307	1.5010967
H	-1.4148910	-3.6215807	1.2915357
C	0.7251098	-0.9895838	-1.5550877
C	0.6699332	-2.8867992	0.8365356
H	0.9915586	-3.5491566	0.0226656
C	-2.9296476	0.4028453	0.0859773
H	-3.1705214	1.2732733	0.7118743
C	0.6850554	-1.2759079	2.4872216
H	1.0208687	-0.4642407	3.1477385
C	1.3443392	-1.6716776	-2.7425605
H	0.5757425	-2.1441816	-3.4009562
H	1.9826112	-2.5216444	-2.3925376
C	-2.7224785	-1.7619973	-0.6717649
H	-2.7843924	-2.8570136	-0.7386100
C	3.0327508	0.2490972	-2.6306281
H	3.6817370	0.9224029	-3.2372699
H	3.7191703	-0.3840185	-2.0191205
C	1.4617904	-1.8733839	1.4588547
H	2.4793185	-1.5817784	1.1685378
C	-3.1603933	-0.9574224	0.4271764
H	-3.5915241	-1.3263651	1.3681558
C	-2.3563721	0.4484314	-1.2256770
H	-2.0749629	1.3536177	-1.7799164
C	2.2014632	-0.6646803	-3.5529990
H	2.8681458	-1.2053784	-4.2637371
H	1.5253226	-0.0302719	-4.1744196
C	-2.2460038	-0.8926840	-1.7000510
H	-1.8750223	-1.2022191	-2.6847939
H	0.5420494	2.4028163	-0.1233538

vibrational spectrum

# mode	symmetry	wave number cm**(-1)	IR intensity km/mol	selection rules IR	RAMAN
1		-0.00	0.00000	-	-
2		-0.00	0.00000	-	-
3		-0.00	0.00000	-	-
4		-0.00	0.00000	-	-
5		0.00	0.00000	-	-
6		0.00	0.00000	-	-
7	a	22.02	0.24720	YES	YES
8	a	32.86	0.01924	YES	YES
9	a	47.09	0.65091	YES	YES
10	a	51.29	0.13635	YES	YES
11	a	54.57	0.11406	YES	YES
12	a	66.23	0.27628	YES	YES
13	a	83.91	0.31956	YES	YES
14	a	111.84	0.02422	YES	YES
15	a	121.23	0.09183	YES	YES
16	a	140.72	0.28355	YES	YES
17	a	149.61	0.13619	YES	YES
18	a	152.94	0.14725	YES	YES
19	a	172.03	1.19126	YES	YES
20	a	211.12	0.49822	YES	YES
21	a	223.78	3.36626	YES	YES
22	a	233.53	1.45235	YES	YES
23	a	246.27	1.64651	YES	YES
24	a	249.74	2.61368	YES	YES
25	a	261.33	0.39858	YES	YES
26	a	276.40	4.29925	YES	YES
27	a	303.47	10.23890	YES	YES
28	a	310.51	7.27414	YES	YES
29	a	313.37	29.66264	YES	YES
30	a	385.19	0.42447	YES	YES
31	a	413.18	1.64874	YES	YES
32	a	431.29	1.21459	YES	YES
33	a	459.69	0.35157	YES	YES
34	a	523.40	1.68760	YES	YES
35	a	592.37	1.34817	YES	YES
36	a	595.15	0.29408	YES	YES
37	a	597.38	1.02632	YES	YES
38	a	599.02	0.44484	YES	YES
39	a	612.58	2.02464	YES	YES
40	a	646.10	0.09296	YES	YES
41	a	684.19	3.00397	YES	YES
42	a	693.43	39.69276	YES	YES
43	a	750.25	13.66286	YES	YES
44	a	755.43	28.86291	YES	YES
45	a	766.18	30.19758	YES	YES
46	a	772.91	158.67998	YES	YES
47	a	777.60	60.57122	YES	YES
48	a	784.90	2.20608	YES	YES
49	a	791.42	4.07810	YES	YES
50	a	804.16	2.96053	YES	YES

A-F_1-1

SCF Energy (au) BP86/SV(P) -1014.512856011
SCF Energy (au) PBE0/def2-TZVPP -1014.372698377
SCF Energy (au) PBE0/def2-TZVPP -1014.3858720393 (CH_2Cl_2 Correction)
SCF Energy (au) PBE0/def2-TZVPP -1014.3793277811 (C_6H_6 Correction)
SCF Energy (au) PBE0/def2-TZVPP -1014.3784901118 (C_6H_{12} Correction)
Zero Point Energy (au) 0.3628730
Chemical Potential (kJ mol^{-1}) 812.05
Dispersion Correction (au) PBE0/def2-TZVPP -0.06218706

xyz coordinates
46

Zr	-0.6056638	-0.7243008	0.1883750
H	-0.1920965	4.9590281	3.2392393
H	-0.7048271	0.6440439	3.2679190
N	-0.5671901	1.5089204	1.3755270
C	-0.3029665	3.9922892	2.7222091
C	-0.3221328	3.9327532	1.3243795
H	-0.2343234	4.8253003	0.6874170
C	-0.5768567	1.5946289	2.7336837
C	-0.4432537	2.7926408	3.4417060
H	-0.4548387	2.7783181	4.5421963
C	1.0755986	0.2054712	-0.9404644
C	-0.5621113	-1.8086714	2.5486151
H	-1.2616926	-1.4711648	3.3270044
C	2.1923902	1.1009472	-1.4182745
H	2.7913951	1.5149002	-0.5712408
H	1.7592068	1.9940569	-1.9321006
C	-0.4691055	2.6747045	0.7206049
C	-0.8086202	-2.8607705	1.6215176
H	-1.7331318	-3.4474983	1.5474103
C	0.7469411	-0.9496189	-1.5211575
C	0.3686953	-3.0431310	0.8277916
H	0.5030015	-3.7830496	0.0273385
C	-3.0002084	0.3321406	-0.1096081
H	-3.2705885	1.2423635	0.4438904
C	0.7881889	-1.3583707	2.3420642
H	1.3104224	-0.5684416	2.9000483
C	1.4154053	-1.5592716	-2.7215733
H	0.6781183	-1.9786575	-3.4480687
H	2.0316423	-2.4357317	-2.3985767
C	-2.7534876	-1.8826378	-0.6916411
H	-2.8050680	-2.9800560	-0.6777971
C	3.1182645	0.3310573	-2.3968015
H	3.7953685	1.0392169	-2.9285642
H	3.7771706	-0.3531469	-1.8102064
C	1.3589091	-2.1327843	1.3023436
H	2.3664475	-2.0041841	0.8871741
C	-3.2326597	-1.0020495	0.3277553
H	-3.6939177	-1.2994880	1.2798849
C	-2.3837409	0.2825515	-1.3992832
H	-2.0863840	1.1424964	-2.0111399
C	2.3163143	-0.5039902	-3.4164286
H	3.0070371	-0.9988777	-4.1376381
H	1.6717423	0.1821523	-4.0158221
C	-2.2393018	-1.0901684	-1.7624871
H	-1.8340855	-1.4688869	-2.7090064
F	-0.5340068	2.6349666	-0.6082149

vibrational spectrum

# mode	symmetry	wave number cm**(-1)	IR intensity km/mol	selection rules IR	RAMAN
1		-0.00	0.00000	-	-
2		-0.00	0.00000	-	-
3		-0.00	0.00000	-	-
4		-0.00	0.00000	-	-
5		-0.00	0.00000	-	-
6		0.00	0.00000	-	-
7	a	17.49	0.74190	YES	YES
8	a	38.76	0.67560	YES	YES
9	a	47.59	0.00399	YES	YES
10	a	51.57	0.35381	YES	YES
11	a	54.47	0.18405	YES	YES
12	a	78.93	0.08605	YES	YES
13	a	86.84	0.13076	YES	YES
14	a	117.53	0.50819	YES	YES
15	a	118.52	0.03980	YES	YES
16	a	126.30	0.27557	YES	YES
17	a	133.00	0.07014	YES	YES
18	a	140.68	0.71130	YES	YES
19	a	142.50	0.23537	YES	YES
20	a	208.30	0.94659	YES	YES
21	a	219.70	4.45778	YES	YES
22	a	225.05	1.79307	YES	YES
23	a	234.18	1.46907	YES	YES
24	a	244.84	2.60511	YES	YES
25	a	248.72	0.71138	YES	YES
26	a	265.62	1.31545	YES	YES
27	a	272.45	6.63270	YES	YES
28	a	299.02	13.03848	YES	YES
29	a	306.15	3.34734	YES	YES
30	a	310.99	27.62878	YES	YES
31	a	412.81	2.56891	YES	YES
32	a	433.89	1.42664	YES	YES
33	a	445.70	1.07992	YES	YES
34	a	459.34	0.13494	YES	YES
35	a	513.70	3.11020	YES	YES
36	a	521.79	3.02999	YES	YES
37	a	547.52	3.93592	YES	YES
38	a	591.62	0.58622	YES	YES
39	a	593.96	0.31150	YES	YES
40	a	595.32	1.48666	YES	YES
41	a	598.56	0.58866	YES	YES
42	a	619.45	0.69153	YES	YES
43	a	681.97	0.44722	YES	YES
44	a	724.02	6.33834	YES	YES
45	a	748.68	34.09556	YES	YES
46	a	759.40	52.85186	YES	YES
47	a	764.36	11.62241	YES	YES
48	a	772.72	169.61351	YES	YES
49	a	775.91	42.28608	YES	YES
50	a	782.85	15.09472	YES	YES

A-F1-2

SCF Energy (au) BP86/SV(P) -1014.518231267
SCF Energy (au) PBE0/def2-TZVPP -1014.379553892
SCF Energy (au) PBE0/def2-TZVPP -1014.3916763756 (CH_2Cl_2 Correction)
SCF Energy (au) PBE0/def2-TZVPP -1014.3856753046 (C_6H_6 Correction)
SCF Energy (au) PBE0/def2-TZVPP -1014.3849040953 (C_6H_{12} Correction)
Zero Point Energy (au) 0.3629388
Chemical Potential (kJ mol^{-1}) 811.68
Dispersion Correction (au) PBE0/def2-TZVPP -0.06228315

xyz coordinates
46

Zr	-0.5407216	-0.7158136	0.2297361
H	-0.2748499	4.9382224	3.3557500
F	-1.8967385	0.7327249	3.0522707
N	-0.4275829	1.4885207	1.4886480
C	-0.3163410	3.9712411	2.8282872
C	0.4261199	3.7520358	1.6554123
H	1.0689241	4.5363724	1.2279728
C	-1.1258104	1.7300655	2.6019128
C	-1.1177205	2.9335509	3.3204526
H	-1.7293734	3.0291168	4.2297671
C	1.0220508	0.2165762	-1.0815098
C	-0.6664386	-2.1259462	2.4388153
H	-1.5764538	-2.0853405	3.0511599
C	2.0652306	1.0949547	-1.7445175
H	2.7347577	1.5954510	-1.0014853
H	1.5589378	1.9323296	-2.2892920
C	0.3429967	2.5052803	1.0234095
C	-0.4148109	-3.0254580	1.3584638
H	-1.0887077	-3.8201712	1.0113382
C	0.7021670	-0.9959611	-1.5469624
C	0.8938378	-2.7574180	0.8468740
H	1.3954540	-3.3028443	0.0380022
C	-2.9048140	0.4362283	-0.0049009
H	-3.1301111	1.3681283	0.5317148
C	0.4840878	-1.2978041	2.5864945
H	0.6202983	-0.5121333	3.3424179
C	1.2795627	-1.6730337	-2.7578442
H	0.4912326	-2.1691169	-3.3744103
H	1.9547542	-2.5049258	-2.4343785
C	-2.7472530	-1.7946255	-0.5502135
H	-2.8413061	-2.8883294	-0.5133815
C	2.9291209	0.2804236	-2.7416856
H	3.5306195	0.9662036	-3.3822398
H	3.6597780	-0.3357863	-2.1653496
C	1.4471884	-1.6826253	1.6063466
H	2.4370573	-1.2315746	1.4584074
C	-3.1585365	-0.8816661	0.4694589
H	-3.5952416	-1.1470271	1.4412118
C	-2.3407007	0.3457452	-1.3145114
H	-2.0363139	1.1878605	-1.9498719
C	2.0714859	-0.6540792	-3.6168783
H	2.7121530	-1.1845712	-4.3585185
H	1.3508768	-0.0374045	-4.2052347
C	-2.2522539	-1.0372336	-1.6571840
H	-1.8962570	-1.4455269	-2.6111248
H	0.8996451	2.2653846	0.1071702

vibrational spectrum

# mode	symmetry	wave number cm**(-1)	IR intensity km/mol	selection rules IR	RAMAN
1		-0.00	0.00000	-	-
2		-0.00	0.00000	-	-
3		-0.00	0.00000	-	-
4		0.00	0.00000	-	-
5		0.00	0.00000	-	-
6		0.00	0.00000	-	-
7	a	20.22	0.34625	YES	YES
8	a	31.12	0.27330	YES	YES
9	a	45.98	0.11911	YES	YES
10	a	47.87	0.54819	YES	YES
11	a	58.84	0.11963	YES	YES
12	a	68.67	0.55588	YES	YES
13	a	79.67	0.82551	YES	YES
14	a	105.90	0.14224	YES	YES
15	a	120.55	1.05537	YES	YES
16	a	126.38	0.05150	YES	YES
17	a	130.18	0.11985	YES	YES
18	a	145.93	0.24065	YES	YES
19	a	150.07	0.47494	YES	YES
20	a	211.99	0.21317	YES	YES
21	a	225.17	3.47113	YES	YES
22	a	233.86	0.14020	YES	YES
23	a	236.62	1.55584	YES	YES
24	a	252.71	3.26876	YES	YES
25	a	253.72	2.13251	YES	YES
26	a	261.39	0.90753	YES	YES
27	a	277.31	3.14931	YES	YES
28	a	298.03	9.05218	YES	YES
29	a	311.40	11.21565	YES	YES
30	a	312.91	30.16867	YES	YES
31	a	416.84	2.44274	YES	YES
32	a	428.85	1.57540	YES	YES
33	a	441.69	0.23834	YES	YES
34	a	460.27	0.36396	YES	YES
35	a	511.59	2.70934	YES	YES
36	a	524.33	3.28884	YES	YES
37	a	549.19	3.78107	YES	YES
38	a	591.42	0.84871	YES	YES
39	a	595.67	0.99645	YES	YES
40	a	597.74	1.12641	YES	YES
41	a	599.84	0.08598	YES	YES
42	a	620.85	2.51589	YES	YES
43	a	684.89	2.30313	YES	YES
44	a	726.76	0.66257	YES	YES
45	a	760.22	5.14177	YES	YES
46	a	764.91	51.92422	YES	YES
47	a	769.43	11.49848	YES	YES
48	a	772.02	189.32232	YES	YES
49	a	781.58	75.17478	YES	YES
50	a	787.69	0.05822	YES	YES\

A-F_2

SCF Energy (au) BP86/SV(P)	-1113.683456585	
SCF Energy (au) PBE0/def2-TZVPP	-1113.565091922	
SCF Energy (au) PBE0/def2-TZVPP	-1113.5784284631	(CH_2Cl_2 Correction)
SCF Energy (au) PBE0/def2-TZVPP	-1113.5718235667	(C_6H_6 Correction)
SCF Energy (au) PBE0/def2-TZVPP	-1113.5709749946	(C_6H_{12} Correction)
Zero Point Energy (au)	0.3549183	
Chemical Potential (kJ mol^{-1})	787.84	
Dispersion Correction (au) PBE0/def2-TZVPP	-0.06207781	

xyz coordinates
46

Zr	-0.6123484	-0.7538945	0.2085453
H	0.0477655	5.0862051	3.1230484
F	-1.1714034	0.7479528	3.3899210
N	-0.6306080	1.6360395	1.3685073
C	-0.1528965	4.1212631	2.6310529
C	-0.0761695	4.0050100	1.2364946
H	0.1686772	4.8510080	0.5787486
C	-0.7558315	1.8087070	2.6927366
C	-0.5159933	2.9989328	3.3887628
H	-0.6318276	3.0247697	4.4815922
C	1.0642318	0.1718323	-0.9244958
C	-0.6385698	-2.0177596	2.4758939
H	-1.4469553	-1.8128993	3.1889512
C	2.2186315	1.0259828	-1.3943186
H	2.8381687	1.3973321	-0.5418463
H	1.8261782	1.9464540	-1.8916404
C	-0.3446282	2.7502276	0.6733483
C	-0.6553172	-3.0006906	1.4457673
H	-1.4781717	-3.6954245	1.2321966
C	0.6875134	-0.9558760	-1.5344652
C	0.6047990	-2.9601082	0.7642690
H	0.9140025	-3.6098357	-0.0647806
C	-3.0737231	0.1823308	0.1046385
H	-3.3795269	0.9968784	0.7761034
C	0.6423359	-1.3755036	2.4372272
H	0.9898089	-0.5826133	3.1132760
C	1.3199797	-1.5603559	-2.7559470
H	0.5583371	-1.9233539	-3.4878249
H	1.8950138	-2.4746338	-2.4628799
C	-2.7211496	-1.9191578	-0.7683632
H	-2.7112820	-3.0105386	-0.8958609
C	3.1080988	0.2372062	-2.3903201
H	3.8188144	0.9252052	-2.9044256
H	3.7325831	-0.4923691	-1.8210268
C	1.4096927	-1.9687008	1.4002606
H	2.4253677	-1.6772163	1.1040413
C	-3.2287700	-1.2053138	0.3623439
H	-3.6434143	-1.6498896	1.2775827
C	-2.4849807	0.3356976	-1.1950709
H	-2.2572001	1.2828310	-1.6991763
C	2.2668326	-0.5321235	-3.4289670
H	2.9316203	-1.0408020	-4.1647188
H	1.6541736	0.1988576	-4.0085492
C	-2.2843647	-0.9665637	-1.7384924
H	-1.8817968	-1.1975105	-2.7324182
F	-0.3456978	2.6524111	-0.6497215

vibrational spectrum

# mode	symmetry	wave number cm**(-1)	IR intensity km/mol	selection rules IR	RAMAN
1		-0.00	0.00000	-	-
2		-0.00	0.00000	-	-
3		0.00	0.00000	-	-
4		0.00	0.00000	-	-
5		0.00	0.00000	-	-
6		0.00	0.00000	-	-
7	a	24.06	0.68165	YES	YES
8	a	31.07	1.18068	YES	YES
9	a	33.77	0.28742	YES	YES
10	a	51.49	0.12369	YES	YES
11	a	54.19	0.28473	YES	YES
12	a	74.09	0.03333	YES	YES
13	a	85.93	0.13453	YES	YES
14	a	89.16	0.09062	YES	YES
15	a	106.17	0.07221	YES	YES
16	a	122.16	0.34939	YES	YES
17	a	125.81	0.23237	YES	YES
18	a	129.56	0.11935	YES	YES
19	a	143.24	0.28562	YES	YES
20	a	208.33	0.91003	YES	YES
21	a	210.41	0.25445	YES	YES
22	a	224.14	3.21874	YES	YES
23	a	230.54	1.63888	YES	YES
24	a	242.76	2.10603	YES	YES
25	a	246.62	3.15341	YES	YES
26	a	267.70	1.29725	YES	YES
27	a	269.58	1.81694	YES	YES
28	a	274.37	6.55003	YES	YES
29	a	298.14	13.65910	YES	YES
30	a	306.28	8.61432	YES	YES
31	a	315.75	24.41389	YES	YES
32	a	358.16	2.60411	YES	YES
33	a	417.93	3.08359	YES	YES
34	a	458.06	0.19849	YES	YES
35	a	460.35	0.39696	YES	YES
36	a	502.48	0.94226	YES	YES
37	a	522.71	1.47936	YES	YES
38	a	536.85	1.88930	YES	YES
39	a	555.20	5.06997	YES	YES
40	a	591.67	0.99701	YES	YES
41	a	594.69	0.37949	YES	YES
42	a	596.09	0.59062	YES	YES
43	a	598.69	1.10083	YES	YES
44	a	635.71	1.72175	YES	YES
45	a	683.52	0.74190	YES	YES
46	a	708.95	5.78185	YES	YES
47	a	735.39	4.29408	YES	YES
48	a	760.16	3.44643	YES	YES
49	a	767.05	95.35327	YES	YES
50	a	774.04	106.98491	YES	YES

A-F_3

SCF Energy (au) BP86/SV(P)	-1212.851541425	
SCF Energy (au) PBE0/def2-TZVPP	-1212.756457236	
SCF Energy (au) PBE0/def2-TZVPP	-1212.7691116531	(CH_2Cl_2 Correction)
SCF Energy (au) PBE0/def2-TZVPP	-1212.7628778923	(C_6H_6 Correction)
SCF Energy (au) PBE0/def2-TZVPP	-1212.7620722505	(C_6H_{12} Correction)
Zero Point Energy (au)	0.3470141	
Chemical Potential (kJ mol^{-1})	763.53	
Dispersion Correction (au) PBE0/def2-TZVPP	-0.06222332	

xyz coordinates
46

Zr	-0.6160831	-0.7579750	0.2008699
F	0.1404400	5.2478906	3.2439689
F	-1.2257845	0.7576418	3.3771454
N	-0.6460384	1.6411142	1.3658501
C	-0.1249050	4.0904003	2.6389686
C	-0.0372725	3.9996693	1.2449694
H	0.2301885	4.8598796	0.6167779
C	-0.7797647	1.8092879	2.6894234
C	-0.5202960	2.9844396	3.4012397
H	-0.6400897	3.0331880	4.4918349
C	1.0529112	0.1697668	-0.9395101
C	-0.6514006	-2.0207485	2.4693693
H	-1.4713033	-1.8288980	3.1729762
C	2.2025923	1.0253688	-1.4194585
H	2.8195062	1.4110950	-0.5714190
H	1.8051328	1.9372793	-1.9285562
C	-0.3310781	2.7532526	0.6798394
C	-0.6406626	-3.0030530	1.4380664
H	-1.4502245	-3.7101101	1.2141094
C	0.6845203	-0.9668816	-1.5391257
C	0.6270899	-2.9433266	0.7725164
H	0.9565859	-3.5887796	-0.0519329
C	-3.0821872	0.1643464	0.1042280
H	-3.3928219	0.9727632	0.7808398
C	0.6195084	-1.3591163	2.4461864
H	0.9478607	-0.5634359	3.1286387
C	1.3204741	-1.5792370	-2.7546650
H	0.5606008	-1.9530765	-3.4828706
H	1.8997959	-2.4879178	-2.4527760
C	-2.7177430	-1.9293494	-0.7824124
H	-2.7017237	-3.0198537	-0.9167632
C	3.0980311	0.2313605	-2.4056999
H	3.8056025	0.9180716	-2.9257036
H	3.7256789	-0.4887958	-1.8280015
C	1.4084981	-1.9389563	1.4175677
H	2.4235398	-1.6325336	1.1345303
C	-3.2300700	-1.2254777	0.3525050
H	-3.6425729	-1.6784090	1.2645936
C	-2.4939549	0.3294538	-1.1946486
H	-2.2724372	1.2812320	-1.6931354
C	2.2623889	-0.5530505	-3.4374457
H	2.9307579	-1.0657639	-4.1670496
H	1.6465580	0.1684846	-4.0254157
C	-2.2871496	-0.9680786	-1.7467786
H	-1.8828501	-1.1903839	-2.7419597
F	-0.3298489	2.6672223	-0.6416869

vibrational spectrum

# mode	symmetry	wave number cm**(-1)	IR intensity km/mol	selection rules IR	RAMAN
1		0.00	0.00000	-	-
2		0.00	0.00000	-	-
3		0.00	0.00000	-	-
4		0.00	0.00000	-	-
5		0.00	0.00000	-	-
6		0.00	0.00000	-	-
7	a	20.47	0.36846	YES	YES
8	a	25.80	0.04502	YES	YES
9	a	32.19	0.10986	YES	YES
10	a	50.41	0.11870	YES	YES
11	a	54.12	0.20025	YES	YES
12	a	72.10	0.00109	YES	YES
13	a	82.66	0.15290	YES	YES
14	a	86.71	0.14364	YES	YES
15	a	102.12	0.01860	YES	YES
16	a	115.71	0.03231	YES	YES
17	a	121.10	0.09986	YES	YES
18	a	128.79	0.15026	YES	YES
19	a	143.28	0.26167	YES	YES
20	a	205.90	1.56363	YES	YES
21	a	209.51	1.24187	YES	YES
22	a	212.31	0.30766	YES	YES
23	a	224.35	3.14439	YES	YES
24	a	231.49	1.49400	YES	YES
25	a	243.52	2.12045	YES	YES
26	a	247.06	3.13044	YES	YES
27	a	268.00	0.85383	YES	YES
28	a	273.72	8.20927	YES	YES
29	a	286.62	1.59953	YES	YES
30	a	303.04	13.40204	YES	YES
31	a	307.35	8.64951	YES	YES
32	a	316.87	24.23725	YES	YES
33	a	334.24	2.41897	YES	YES
34	a	355.75	1.66237	YES	YES
35	a	418.22	3.01863	YES	YES
36	a	459.76	0.10990	YES	YES
37	a	498.53	5.52879	YES	YES
38	a	513.95	6.03579	YES	YES
39	a	524.08	1.40361	YES	YES
40	a	589.97	0.48913	YES	YES
41	a	591.60	1.02844	YES	YES
42	a	594.73	0.33003	YES	YES
43	a	596.12	0.67968	YES	YES
44	a	598.60	1.05973	YES	YES
45	a	602.28	1.68862	YES	YES
46	a	618.42	3.60827	YES	YES
47	a	626.60	1.20818	YES	YES
48	a	684.02	0.91979	YES	YES
49	a	706.73	1.70631	YES	YES
50	a	760.30	4.42411	YES	YES

A-F_4

SCF Energy (au) BP86/SV(P)	-1312.001449269	
SCF Energy (au) PBE0/def2-TZVPP	-1311.927408121	
SCF Energy (au) PBE0/def2-TZVPP	-1311.9398959022	(CH_2Cl_2 Correction)
SCF Energy (au) PBE0/def2-TZVPP	-1311.9337074169	(C_6H_6 Correction)
SCF Energy (au) PBE0/def2-TZVPP	-1311.9329128457	(C_6H_{12} Correction)
Zero Point Energy (au)	0.3388750	
Chemical Potential (kJ mol^{-1})	738.63	
Dispersion Correction (au) PBE0/def2-TZVPP	-0.06250936	

xyz coordinates
46

Zr	-0.6230235	-0.7724790	0.2035335
H	0.0780540	5.0985715	3.0931779
F	-1.1319746	0.7512135	3.4153167
N	-0.6369331	1.6455329	1.3669879
C	-0.1262916	4.1315044	2.6103333
C	-0.0824069	3.9924450	1.2196241
F	0.2030491	5.0312855	0.4318360
C	-0.7330996	1.7959915	2.6917781
C	-0.4677933	3.0034498	3.3622256
F	-0.5678760	3.0611531	4.6925619
C	1.0506152	0.1717287	-0.9195171
C	-0.6488594	-2.0294111	2.4694242
H	-1.4542906	-1.8230009	3.1855583
C	2.2038993	1.0334631	-1.3778811
H	2.8154374	1.4040886	-0.5193405
H	1.8112668	1.9540040	-1.8745755
C	-0.3632791	2.7384120	0.6437937
C	-0.6693371	-3.0144425	1.4412993
H	-1.4939556	-3.7079110	1.2306303
C	0.6856446	-0.9577400	-1.5334777
C	0.5900724	-2.9782234	0.7577952
H	0.8965737	-3.6307994	-0.0700460
C	-3.0801347	0.1653167	0.1011040
H	-3.3875355	0.9782204	0.7740602
C	0.6346875	-1.3916418	2.4284757
H	0.9865209	-0.6012878	3.1052520
C	1.3309278	-1.5563711	-2.7509835
H	0.5764616	-1.9230754	-3.4881694
H	1.9094274	-2.4672091	-2.4544612
C	-2.7290441	-1.9347334	-0.7762245
H	-2.7201619	-3.0258491	-0.9058955
C	3.1048350	0.2519924	-2.3695437
H	3.8138633	0.9460785	-2.8773005
H	3.7305040	-0.4743514	-1.7975555
C	1.3988506	-1.9888100	1.3918274
H	2.4152684	-1.7011787	1.0943078
C	-3.2353136	-1.2230675	0.3563938
H	-3.6506409	-1.6690528	1.2705877
C	-2.4925782	0.3210170	-1.1988169
H	-2.2678906	1.2688331	-1.7030010
C	2.2755541	-0.5205559	-3.4152818
H	2.9486041	-1.0239018	-4.1469746
H	1.6623047	0.2072764	-3.9979815
C	-2.2921847	-0.9803429	-1.7446613
H	-1.8905943	-1.2092139	-2.7394688
F	-0.3772226	2.6530719	-0.6767268

vibrational spectrum

# mode	symmetry	wave number cm**(-1)	IR intensity km/mol	selection rules IR	RAMAN
1		-0.00	0.00000	-	-
2		0.00	0.00000	-	-
3		0.00	0.00000	-	-
4		0.00	0.00000	-	-
5		0.00	0.00000	-	-
6		0.00	0.00000	-	-
7	a	19.22	0.41735	YES	YES
8	a	25.59	0.13211	YES	YES
9	a	32.40	0.08521	YES	YES
10	a	47.52	0.04459	YES	YES
11	a	53.00	0.18754	YES	YES
12	a	68.31	0.03016	YES	YES
13	a	74.41	0.14128	YES	YES
14	a	83.00	0.05964	YES	YES
15	a	84.86	0.09551	YES	YES
16	a	111.89	0.22491	YES	YES
17	a	120.59	0.06870	YES	YES
18	a	128.86	0.11824	YES	YES
19	a	141.43	0.15571	YES	YES
20	a	142.89	0.14360	YES	YES
21	a	203.11	2.57167	YES	YES
22	a	209.94	1.02657	YES	YES
23	a	225.10	3.63087	YES	YES
24	a	231.72	1.75856	YES	YES
25	a	244.57	2.75926	YES	YES
26	a	247.25	2.68433	YES	YES
27	a	267.57	0.80833	YES	YES
28	a	273.67	7.06130	YES	YES
29	a	283.13	0.84527	YES	YES
30	a	292.48	4.08981	YES	YES
31	a	300.72	9.94687	YES	YES
32	a	304.12	5.49844	YES	YES
33	a	307.85	3.22018	YES	YES
34	a	316.63	23.77949	YES	YES
35	a	359.65	2.79307	YES	YES
36	a	417.53	3.44074	YES	YES
37	a	428.99	1.81217	YES	YES
38	a	439.17	0.35926	YES	YES
39	a	459.45	0.01972	YES	YES
40	a	470.96	0.94973	YES	YES
41	a	494.14	2.13266	YES	YES
42	a	521.55	2.07618	YES	YES
43	a	591.54	1.10786	YES	YES
44	a	594.42	0.28431	YES	YES
45	a	595.89	0.61422	YES	YES
46	a	598.49	1.00998	YES	YES
47	a	650.73	0.37366	YES	YES
48	a	651.22	1.02399	YES	YES
49	a	667.01	2.93429	YES	YES
50	a	682.39	13.14211	YES	YES

A-F$_5$

SCF Energy (au) BP86/SV(P)	-1411.156590632	
SCF Energy (au) PBE0/def2-TZVPP	1411.105927493	
SCF Energy (au) PBE0/def2-TZVPP	-1411.1170452353	(CH_2Cl_2 Correction)
SCF Energy (au) PBE0/def2-TZVPP	-1411.1115563500	(C_6H_6 Correction)
SCF Energy (au) PBE0/def2-TZVPP	-1411.1108487867	(C_6H_{12} Correction)
Zero Point Energy (au)	0.3310477	
Chemical Potential (kJ mol^{-1})	714.20	
Dispersion Correction (au) PBE0/def2-TZVPP	-0.06267046	

xyz coordinates
46

Zr	-0.6335921	-0.7841334	0.1936231
F	0.2152884	5.2610388	3.1989755
F	-1.1684330	0.7644894	3.4147805
N	-0.6637413	1.6520111	1.3676862
C	-0.0705726	4.1064925	2.6134349
C	-0.0426317	3.9860076	1.2126658
F	0.2625074	5.0315692	0.4474633
C	-0.7460851	1.7996911	2.6928244
C	-0.4421526	2.9868954	3.3783132
F	-0.5262279	3.0632732	4.7052128
C	1.0309534	0.1733510	-0.9267761
C	-0.6617348	-2.0262031	2.4680636
H	-1.4713815	-1.8209366	3.1798231
C	2.1757731	1.0453523	-1.3884547
H	2.7796486	1.4302763	-0.5306708
H	1.7736911	1.9571769	-1.8936895
C	-0.3670283	2.7404440	0.6476954
C	-0.6715000	-3.0178328	1.4460204
H	-1.4910692	-3.7174316	1.2357980
C	0.6845692	-0.9662163	-1.5346246
C	0.5905546	-2.9779509	0.7676317
H	0.9048689	-3.6339984	-0.0545184
C	-3.0933067	0.1416277	0.0809746
H	-3.4061571	0.9544375	0.7514761
C	0.6182618	-1.3809441	2.4285187
H	0.9635599	-0.5854413	3.1028447
C	1.3421919	-1.5647253	-2.7452488
H	0.5950330	-1.9465193	-3.4821584
H	1.9310899	-2.4659512	-2.4399995
C	-2.7289889	-1.9585411	-0.7906700
H	-2.7145954	-3.0499090	-0.9179479
C	3.0908599	0.2686728	-2.3706415
H	3.7929164	0.9684001	-2.8802214
H	3.7235206	-0.4453751	-1.7909567
C	1.3906290	-1.9796729	1.3989161
H	2.4066869	-1.6879612	1.1041720
C	-3.2436172	-1.2468173	0.3381792
H	-3.6607078	-1.6929285	1.2515156
C	-2.5008228	0.2974416	-1.2167177
H	-2.2785041	1.2451874	-1.7222163
C	2.2758870	-0.5219772	-3.4139429
H	2.9582383	-1.0220341	-4.1391713
H	1.6556631	0.1934403	-4.0045616
C	-2.2927376	-1.0041865	-1.7594007
H	-1.8859896	-1.2331675	-2.7520776
F	-0.4008152	2.6635787	-0.6719425

vibrational spectrum

# mode	symmetry	wave number cm**(-1)	IR intensity km/mol	selection rules IR	RAMAN
1		-0.00	0.00000	-	-
2		0.00	0.00000	-	-
3		0.00	0.00000	-	-
4		0.00	0.00000	-	-
5		0.00	0.00000	-	-
6		0.00	0.00000	-	-
7	a	16.81	0.21350	YES	YES
8	a	22.58	0.01345	YES	YES
9	a	32.11	0.05669	YES	YES
10	a	46.25	0.05386	YES	YES
11	a	52.49	0.13154	YES	YES
12	a	67.47	0.07377	YES	YES
13	a	70.60	0.08831	YES	YES
14	a	80.75	0.09114	YES	YES
15	a	83.28	0.12017	YES	YES
16	a	110.47	0.05704	YES	YES
17	a	111.58	0.13781	YES	YES
18	a	128.16	0.16959	YES	YES
19	a	141.66	0.20697	YES	YES
20	a	142.55	0.09674	YES	YES
21	a	151.33	0.02098	YES	YES
22	a	209.39	0.97939	YES	YES
23	a	215.80	3.05878	YES	YES
24	a	225.76	3.67353	YES	YES
25	a	232.04	1.75809	YES	YES
26	a	244.85	3.08035	YES	YES
27	a	247.67	2.42187	YES	YES
28	a	258.87	0.20244	YES	YES
29	a	267.11	0.50524	YES	YES
30	a	271.09	0.48836	YES	YES
31	a	274.89	7.29526	YES	YES
32	a	296.26	11.85052	YES	YES
33	a	306.08	9.42743	YES	YES
34	a	315.42	17.71930	YES	YES
35	a	318.24	7.38017	YES	YES
36	a	338.76	0.16793	YES	YES
37	a	360.39	3.85581	YES	YES
38	a	402.67	1.11118	YES	YES
39	a	417.80	3.19004	YES	YES
40	a	444.75	0.05806	YES	YES
41	a	459.58	0.07860	YES	YES
42	a	462.18	0.00452	YES	YES
43	a	521.95	1.86682	YES	YES
44	a	571.07	0.11530	YES	YES
45	a	582.09	2.01592	YES	YES
46	a	591.53	1.09822	YES	YES
47	a	594.44	0.20184	YES	YES
48	a	595.86	0.61208	YES	YES
49	a	598.46	0.96118	YES	YES
50	a	628.23	0.52128	YES	YES

TS_{AB}-F_1

SCF Energy (au) BP86/SV(P) -1014.495826923
SCF Energy (au) PBE0/def2-TZVPP -1014.352336376
SCF Energy (au) PBE0/def2-TZVPP -1014.3624407338 (CH_2Cl_2 Correction)
SCF Energy (au) PBE0/def2-TZVPP -1014.3574644742 (C_6H_6 Correction)
SCF Energy (au) PBE0/def2-TZVPP -1014.3568215811 (C_6H_{12} Correction)
Zero Point Energy (au) 0.3623772
Chemical Potential (kJ mol^{-1}) 816.40
Dispersion Correction (au) PBE0/def2-TZVPP -0.06509735

xyz coordinates
46

Zr	-0.6291205	-0.8680831	-0.1241251
H	-2.2286627	-0.6422506	3.0348300
N	-1.5500795	0.5570401	1.4707041
C	-0.4904298	2.2897081	3.3830079
C	-0.3228493	2.5756479	2.0370153
C	-1.7014058	0.2968860	2.7941066
C	-1.2198890	1.1386429	3.7903456
C	0.7575266	1.0543380	-0.4603042
C	-0.0508679	-3.2855990	0.5189174
H	-0.6231707	-4.1003075	0.0576467
C	1.6264996	2.2848815	-0.4059259
H	1.9716839	2.4926016	0.6346826
H	1.0319458	3.1802881	-0.7105385
C	-0.9086942	1.6869606	1.0714335
C	1.1687895	-2.7287476	0.0183550
H	1.6896472	-3.0310414	-0.9004133
C	0.8770386	0.0947145	-1.3774345
C	1.6210884	-1.7575431	0.9600282
H	2.5205267	-1.1367927	0.8612333
C	-3.1747952	-0.7986498	-0.7235085
H	-3.8725080	-0.3600720	0.0021366
C	-0.3751678	-2.6233185	1.7366978
H	-1.2377118	-2.8447550	2.3803669
C	1.8705836	0.0984460	-2.5097074
H	1.4027368	-0.2489595	-3.4615038
H	2.6759357	-0.6471411	-2.2938144
C	-1.8377144	-2.2960448	-1.8582308
H	-1.3680725	-3.2292919	-2.1976303
C	2.8580524	2.1265451	-1.3325599
H	3.3506891	3.1150300	-1.4766991
H	3.6086100	1.4705351	-0.8309322
C	0.6690480	-1.6750195	2.0070071
H	0.7267403	-1.0054646	2.8757746
C	-2.7854341	-2.1626071	-0.7896796
H	-3.1471113	-2.9707224	-0.1387278
C	-2.4761108	-0.0828888	-1.7449686
H	-2.5476550	0.9938453	-1.9404452
C	2.4849382	1.5062332	-2.6908961
H	3.3786291	1.4570451	-3.3539325
H	1.7442502	2.1652662	-3.2021141
C	-1.6655812	-1.0127832	-2.4604321
H	-1.0342244	-0.7869529	-3.3288366
F	-1.4880937	2.3264403	-0.0086082
H	-0.0813795	2.9803745	4.1402242
H	-1.4026749	0.9150886	4.8516219
H	0.1844445	3.4884772	1.6958334

vibrational spectrum

# mode	symmetry	wave number cm**(-1)	IR intensity km/mol	selection rules IR	RAMAN
1	a	-166.68	0.00000	YES	YES
2		-0.00	0.00000	-	-
3		-0.00	0.00000	-	-
4		-0.00	0.00000	-	-
5		-0.00	0.00000	-	-
6		0.00	0.00000	-	-
7		0.00	0.00000	-	-
8	a	19.70	0.05104	YES	YES
9	a	41.13	0.15473	YES	YES
10	a	48.95	0.06478	YES	YES
11	a	54.47	0.22085	YES	YES
12	a	75.07	1.55817	YES	YES
13	a	90.32	0.36172	YES	YES
14	a	113.93	0.61835	YES	YES
15	a	129.20	0.22130	YES	YES
16	a	137.10	0.44700	YES	YES
17	a	143.79	0.61449	YES	YES
18	a	161.43	1.12151	YES	YES
19	a	187.01	0.57706	YES	YES
20	a	205.37	11.07069	YES	YES
21	a	222.77	0.38606	YES	YES
22	a	225.89	1.33287	YES	YES
23	a	231.23	3.50197	YES	YES
24	a	242.35	1.63731	YES	YES
25	a	258.01	0.39481	YES	YES
26	a	281.48	4.21314	YES	YES
27	a	292.27	7.49121	YES	YES
28	a	300.64	17.49084	YES	YES
29	a	312.75	2.53037	YES	YES
30	a	334.18	13.91735	YES	YES
31	a	374.89	12.45024	YES	YES
32	a	426.65	7.11152	YES	YES
33	a	447.68	12.81031	YES	YES
34	a	461.98	0.69103	YES	YES
35	a	484.40	5.67086	YES	YES
36	a	510.84	8.44167	YES	YES
37	a	527.97	37.89608	YES	YES
38	a	592.56	0.36919	YES	YES
39	a	594.49	0.15374	YES	YES
40	a	595.48	0.76850	YES	YES
41	a	598.20	0.67354	YES	YES
42	a	614.66	13.50980	YES	YES
43	a	688.01	1.42576	YES	YES
44	a	703.46	90.96815	YES	YES
45	a	727.84	112.21949	YES	YES
46	a	770.58	12.86320	YES	YES
47	a	775.15	108.37473	YES	YES
48	a	782.36	24.60553	YES	YES
49	a	782.91	108.30024	YES	YES
50	a	786.17	90.09376	YES	YES

TS_{AB}-F_2

SCF Energy (au) BP86/SV(P)	-1113.673574062	
SCF Energy (au) PBE0/def2-TZVPP	-1113.552031308	
SCF Energy (au) PBE0/def2-TZVPP	-1113.5613205067	(CH_2Cl_2 Correction)
SCF Energy (au) PBE0/def2-TZVPP	-1113.5568359437	(C_6H_6 Correction)
SCF Energy (au) PBE0/def2-TZVPP	-1113.5562355960	(C_6H_{12} Correction)
Zero Point Energy (au)	0.3546902	
Chemical Potential (kJ mol^{-1})	794.29	
Dispersion Correction (au) PBE0/def2-TZVPP	-0.06543014	

xyz coordinates
46

Zr	-0.6331295	-0.8458026	-0.1035136
F	-2.4267403	-0.6758892	3.0704669
N	-1.5766739	0.6661279	1.4598662
C	-0.4105246	2.3229947	3.3717315
C	-0.2536505	2.6241701	2.0248635
C	-1.7178096	0.4257119	2.7679892
C	-1.1840610	1.2074213	3.7856388
C	0.7970105	1.0135191	-0.4957665
C	0.0936501	-3.2394019	0.3976999
H	-0.3150350	-4.0473553	-0.2229094
C	1.6847564	2.2306816	-0.4667867
H	2.0538975	2.4423942	0.5651283
H	1.1006618	3.1337806	-0.7687819
C	-0.8894351	1.7740327	1.0659363
C	1.3076029	-2.5247517	0.1454603
H	1.9856944	-2.6816488	-0.7034727
C	0.8788339	0.0460401	-1.4058203
C	1.5107169	-1.6192887	1.2297313
H	2.3435379	-0.9114140	1.3306957
C	-3.1897168	-0.9616793	-0.5989620
H	-3.8878316	-0.6759312	0.1988295
C	-0.4665338	-2.7549533	1.6165410
H	-1.3874821	-3.1084528	2.0981143
C	1.8422555	0.0233834	-2.5633733
H	1.3435841	-0.3177083	-3.5017736
H	2.6386004	-0.7366607	-2.3660429
C	-1.7828639	-2.1915224	-1.9496796
H	-1.2446524	-3.0330516	-2.4072967
C	2.8937549	2.0413727	-1.4180810
H	3.4028364	3.0186286	-1.5808239
H	3.6412116	1.3737188	-0.9274354
C	0.4199131	-1.7542003	2.1287393
H	0.2968903	-1.2015292	3.0694931
C	-2.6950090	-2.2664989	-0.8459084
H	-2.9500844	-3.1709484	-0.2769315
C	-2.6059479	-0.0733549	-1.5621161
H	-2.7872217	1.0057096	-1.6353167
C	2.4791216	1.4192514	-2.7640852
H	3.3571461	1.3500002	-3.4459984
H	1.7401068	2.0895391	-3.2630994
C	-1.7581854	-0.8395261	-2.4105357
H	-1.1869932	-0.4612011	-3.2673998
F	-1.4026087	2.4109611	-0.0340624
H	0.0384066	2.9825876	4.1331647
H	-1.3788502	0.9692929	4.8399816
H	0.2808511	3.5214511	1.6859018

vibrational spectrum

# mode	symmetry	wave number cm**(-1)	IR intensity km/mol	selection rules IR	RAMAN
1	a	-137.59	0.00000	YES	YES
2		0.00	0.00000	-	-
3		0.00	0.00000	-	-
4		0.00	0.00000	-	-
5		0.00	0.00000	-	-
6		0.00	0.00000	-	-
7		0.00	0.00000	-	-
8	a	23.52	0.03761	YES	YES
9	a	39.67	0.09743	YES	YES
10	a	49.54	0.02555	YES	YES
11	a	55.26	0.59768	YES	YES
12	a	75.47	1.85769	YES	YES
13	a	90.91	0.58067	YES	YES
14	a	108.74	1.23906	YES	YES
15	a	125.32	0.17408	YES	YES
16	a	130.36	0.21613	YES	YES
17	a	134.71	0.94920	YES	YES
18	a	145.45	1.06939	YES	YES
19	a	157.53	0.59023	YES	YES
20	a	204.32	13.02749	YES	YES
21	a	218.37	1.47829	YES	YES
22	a	229.00	2.01388	YES	YES
23	a	233.24	2.52429	YES	YES
24	a	239.47	1.15276	YES	YES
25	a	250.37	1.97175	YES	YES
26	a	267.74	1.10180	YES	YES
27	a	282.54	5.48882	YES	YES
28	a	292.98	3.81145	YES	YES
29	a	297.81	15.92383	YES	YES
30	a	308.98	2.32108	YES	YES
31	a	325.01	19.44497	YES	YES
32	a	353.83	15.12387	YES	YES
33	a	426.34	4.55233	YES	YES
34	a	445.78	9.47627	YES	YES
35	a	462.97	0.34718	YES	YES
36	a	497.35	11.51557	YES	YES
37	a	511.36	0.54239	YES	YES
38	a	521.54	13.52615	YES	YES
39	a	552.13	2.13030	YES	YES
40	a	591.49	54.38098	YES	YES
41	a	592.91	1.05430	YES	YES
42	a	596.02	0.31181	YES	YES
43	a	598.71	14.96162	YES	YES
44	a	600.81	47.55663	YES	YES
45	a	686.16	5.06076	YES	YES
46	a	695.97	8.50293	YES	YES
47	a	708.00	64.80646	YES	YES
48	a	741.98	109.98521	YES	YES
49	a	778.77	3.54152	YES	YES
50	a	780.63	140.18378	YES	YES

TS_{AB}-F_3

SCF Energy (au)	BP86/SV(P)	-1212.843539896
SCF Energy (au)	PBE0/def2-TZVPP	-1212.745394619
SCF Energy (au)	PBE0/def2-TZVPP	-1212.7542711724 (CH_2Cl_2 Correction)
SCF Energy (au)	PBE0/def2-TZVPP	-1212.7499442341 (C_6H_6 Correction)
SCF Energy (au)	PBE0/def2-TZVPP	-1212.7493788463 (C_6H_{12} Correction)
Zero Point Energy (au)		0.3467691
Chemical Potential (kJ mol^{-1})		770.20
Dispersion Correction (au)	PBE0/def2-TZVPP	-0.06555543

xyz coordinates
46

Zr	-0.6348696	-0.8525213	-0.1122755
F	-2.3840117	-0.6818536	3.0913424
N	-1.5824165	0.6708087	1.4651194
C	-0.4107792	2.3255419	3.3533364
C	-0.2794607	2.6530425	2.0111010
C	-1.6968937	0.4255499	2.7730974
C	-1.1554410	1.2043078	3.7899974
C	0.7888602	0.9997393	-0.5000808
C	0.0840680	-3.2489620	0.3840178
H	-0.3284439	-4.0542226	-0.2375491
C	1.6721832	2.2199919	-0.4645153
H	2.0324971	2.4342238	0.5702333
H	1.0875436	3.1212357	-0.7716148
C	-0.9160957	1.7921166	1.0714183
C	1.3001447	-2.5377028	0.1309608
H	1.9762187	-2.6951208	-0.7194809
C	0.8785134	0.0358910	-1.4138602
C	1.5093192	-1.6364027	1.2174175
H	2.3456860	-0.9327704	1.3190664
C	-3.1927665	-0.9593417	-0.6063368
H	-3.8912489	-0.6717391	0.1905642
C	-0.4718819	-2.7659429	1.6051417
H	-1.3922408	-3.1189910	2.0882963
C	1.8470316	0.0189850	-2.5669505
H	1.3531366	-0.3180354	-3.5093045
H	2.6428236	-0.7415735	-2.3693529
C	-1.7922181	-2.1939982	-1.9587287
H	-1.2578986	-3.0373761	-2.4173993
C	2.8885132	2.0353211	-1.4073468
H	3.3974320	3.0135848	-1.5640167
H	3.6333305	1.3674858	-0.9130005
C	0.4195427	-1.7695105	2.1179168
H	0.3035360	-1.2227390	3.0630709
C	-2.7042481	-2.2661722	-0.8549507
H	-2.9630614	-3.1700654	-0.2868277
C	-2.6055529	-0.0725865	-1.5690654
H	-2.7849668	1.0065827	-1.6440983
C	2.4840305	1.4163485	-2.7580338
H	3.3669953	1.3500827	-3.4337641
H	1.7481433	2.0875272	-3.2604788
C	-1.7614501	-0.8418222	-2.4183566
H	-1.1894658	-0.4655069	-3.2754990
F	-1.4214966	2.4090779	-0.0360131
F	0.1382767	3.1185577	4.2822979
H	-1.3071656	0.9756362	4.8523165
H	0.2262480	3.5733183	1.6921884

vibrational spectrum

# mode	symmetry	wave number cm**(-1)	IR intensity km/mol	selection rules IR	selection rules RAMAN
1	a	-129.14	0.00000	YES	YES
2		-0.00	0.00000	-	-
3		-0.00	0.00000	-	-
4		-0.00	0.00000	-	-
5		-0.00	0.00000	-	-
6		-0.00	0.00000	-	-
7		0.00	0.00000	-	-
8	a	22.69	0.06324	YES	YES
9	a	36.71	0.02233	YES	YES
10	a	50.69	0.02235	YES	YES
11	a	54.73	0.55914	YES	YES
12	a	61.53	0.39025	YES	YES
13	a	79.80	0.84942	YES	YES
14	a	103.96	1.23969	YES	YES
15	a	122.78	0.52885	YES	YES
16	a	125.03	0.13699	YES	YES
17	a	129.63	0.00628	YES	YES
18	a	144.67	0.88636	YES	YES
19	a	155.81	0.30257	YES	YES
20	a	204.35	13.75040	YES	YES
21	a	210.19	2.14606	YES	YES
22	a	218.90	1.44593	YES	YES
23	a	228.81	1.51845	YES	YES
24	a	233.88	3.60459	YES	YES
25	a	239.45	2.38485	YES	YES
26	a	251.96	1.67526	YES	YES
27	a	275.25	1.75605	YES	YES
28	a	284.73	6.11363	YES	YES
29	a	292.77	3.91174	YES	YES
30	a	297.72	15.79732	YES	YES
31	a	309.04	2.53167	YES	YES
32	a	322.91	18.15138	YES	YES
33	a	340.69	10.63212	YES	YES
34	a	378.67	1.64304	YES	YES
35	a	425.03	7.98154	YES	YES
36	a	462.38	0.20801	YES	YES
37	a	491.51	11.22854	YES	YES
38	a	506.22	21.27895	YES	YES
39	a	515.68	1.20214	YES	YES
40	a	563.84	9.56851	YES	YES
41	a	565.93	173.34414	YES	YES
42	a	592.79	0.57724	YES	YES
43	a	593.68	12.37875	YES	YES
44	a	595.75	8.07499	YES	YES
45	a	596.97	11.10642	YES	YES
46	a	599.05	1.56891	YES	YES
47	a	617.60	5.97295	YES	YES
48	a	682.81	27.18063	YES	YES
49	a	688.02	7.51186	YES	YES
50	a	746.72	16.05291	YES	YES

TS_{AB}-F_4

SCF Energy (au) BP86/SV(P)	-1311.992624443	
SCF Energy (au) PBE0/def2-TZVPP	-1311.913176859	
SCF Energy (au) PBE0/def2-TZVPP	-1311.9223514022	(CH_2Cl_2 Correction)
SCF Energy (au) PBE0/def2-TZVPP	-1311.9178773435	(C_6H_6 Correction)
SCF Energy (au) PBE0/def2-TZVPP	-1311.9172929213	(C_6H_{12} Correction)
Zero Point Energy (au)	0.3386967	
Chemical Potential (kJ mol^{-1})	744.83	
Dispersion Correction (au) PBE0/def2-TZVPP	-0.06592939	

xyz coordinates
46

Zr	-0.6288339	-0.8214002	-0.0773583
F	-2.5324319	-0.6469800	3.0344225
N	-1.5885304	0.7002915	1.4566625
C	-0.4231886	2.2955570	3.4134581
C	-0.2527032	2.5982814	2.0748010
F	0.4237532	3.6993019	1.7097505
C	-1.7818959	0.4276863	2.7543741
C	-1.2206840	1.1831516	3.7764782
F	-1.4535058	0.8937090	5.0687585
C	0.8520221	1.0195603	-0.5163231
C	0.1555066	-3.2022696	0.3733516
H	-0.1746552	-3.9950703	-0.3107786
C	1.7497040	2.2318369	-0.5474781
H	2.1610058	2.4667356	0.4597387
H	1.1618230	3.1324116	-0.8415427
C	-0.8558165	1.7730383	1.0597972
C	1.3525003	-2.4311546	0.2404506
H	2.0941797	-2.5229817	-0.5631483
C	0.8971062	0.0258804	-1.3946268
C	1.4376182	-1.5622407	1.3697541
H	2.2328406	-0.8297365	1.5605490
C	-3.1740742	-1.0405181	-0.5489916
H	-3.8748294	-0.8538242	0.2754530
C	-0.5053025	-2.8003947	1.5737355
H	-1.4399137	-3.2126572	1.9760820
C	1.8242947	-0.0472399	-2.5795507
H	1.2959741	-0.4239500	-3.4873635
H	2.6264502	-0.7994042	-2.3768613
C	-1.7262934	-2.0880907	-2.0076847
H	-1.1467923	-2.8622034	-2.5296415
C	2.9182526	2.0035126	-1.5415051
H	3.4210119	2.9741192	-1.7544559
H	3.6840055	1.3505648	-1.0593580
C	0.2973135	-1.7908143	2.1883538
H	0.0883003	-1.3015069	3.1485288
C	-2.6148934	-2.2938259	-0.9022203
H	-2.8116265	-3.2511183	-0.4008528
C	-2.6607860	-0.0539379	-1.4576368
H	-2.9047359	1.0154063	-1.4512081
C	2.4535255	1.3400959	-2.8501151
H	3.3048597	1.2461169	-3.5621407
H	1.6962288	1.9943659	-3.3427858
C	-1.7890338	-0.7072127	-2.3706179
H	-1.2509136	-0.2342722	-3.2016328
F	-1.3307237	2.4596857	-0.0289636
H	0.0138873	2.9414948	4.1903424

vibrational spectrum

# mode	symmetry	wave number cm**(-1)	IR intensity km/mol	selection rules IR	RAMAN
1	a	-105.80	0.00000	YES	YES
2		-0.00	0.00000	-	-
3		-0.00	0.00000	-	-
4		0.00	0.00000	-	-
5		0.00	0.00000	-	-
6		0.00	0.00000	-	-
7		0.00	0.00000	-	-
8	a	20.23	0.01853	YES	YES
9	a	26.56	0.01031	YES	YES
10	a	44.87	0.38872	YES	YES
11	a	51.01	0.59831	YES	YES
12	a	61.35	1.17018	YES	YES
13	a	81.45	0.62237	YES	YES
14	a	95.30	0.47231	YES	YES
15	a	114.98	0.77756	YES	YES
16	a	123.08	1.13732	YES	YES
17	a	129.60	0.53679	YES	YES
18	a	144.77	1.55230	YES	YES
19	a	154.01	0.17028	YES	YES
20	a	162.26	7.08097	YES	YES
21	a	204.28	14.54630	YES	YES
22	a	218.44	1.10001	YES	YES
23	a	221.61	9.02350	YES	YES
24	a	230.08	0.19995	YES	YES
25	a	240.32	1.86944	YES	YES
26	a	250.67	1.58374	YES	YES
27	a	263.78	0.96454	YES	YES
28	a	270.00	3.32536	YES	YES
29	a	278.88	2.99173	YES	YES
30	a	286.41	0.25941	YES	YES
31	a	293.75	5.28677	YES	YES
32	a	299.86	14.17004	YES	YES
33	a	306.98	2.83978	YES	YES
34	a	322.59	17.27529	YES	YES
35	a	352.56	25.65182	YES	YES
36	a	412.99	11.16734	YES	YES
37	a	416.39	4.66677	YES	YES
38	a	441.23	11.04835	YES	YES
39	a	461.82	1.18637	YES	YES
40	a	466.94	4.12465	YES	YES
41	a	488.18	24.97332	YES	YES
42	a	510.36	10.03111	YES	YES
43	a	591.77	0.77857	YES	YES
44	a	594.77	2.37939	YES	YES
45	a	595.47	0.48663	YES	YES
46	a	598.44	0.57935	YES	YES
47	a	614.25	40.85968	YES	YES
48	a	632.65	86.90961	YES	YES
49	a	648.89	14.21188	YES	YES
50	a	681.76	22.60950	YES	YES

TS_{AB}-F_5

SCF Energy (au) BP86/SV(P)	-1411.149516407	
SCF Energy (au) PBE0/def2-TZVPP	-1411.093659723	
SCF Energy (au) PBE0/def2-TZVPP	-1411.1017374816	(CH_2Cl_2 Correction)
SCF Energy (au) PBE0/def2-TZVPP	-1411.0978185371	(C_6H_6 Correction)
SCF Energy (au) PBE0/def2-TZVPP	-1411.0973037155	(C_6H_{12} Correction)
Zero Point Energy (au)	0.3308871	
Chemical Potential (kJ mol^{-1})	721.10	
Dispersion Correction (au) PBE0/def2-TZVPP	-0.06611996	

xyz coordinates
46

Zr	-0.6320770	-0.8307687	-0.0915999
F	-2.4808449	-0.6691503	3.0575497
N	-1.5968684	0.6938909	1.4601692
C	-0.4212885	2.3010359	3.3905128
C	-0.2839062	2.6267052	2.0455150
F	0.3548905	3.7496743	1.6922348
C	-1.7580333	0.4173167	2.7590104
C	-1.1947652	1.1721658	3.7803177
F	-1.3832551	0.8898668	5.0762953
C	0.8310437	1.0114818	-0.5203242
C	0.1368785	-3.2151782	0.3660160
H	-0.2118252	-4.0105127	-0.3056033
C	1.7268401	2.2257502	-0.5313170
H	2.1215393	2.4564784	0.4840446
H	1.1419523	3.1268478	-0.8304683
C	-0.8909380	1.7830834	1.0578882
C	1.3388386	-2.4560417	0.2070265
H	2.0663168	-2.5611257	-0.6078543
C	0.8905839	0.0247122	-1.4065574
C	1.4528195	-1.5823251	1.3298441
H	2.2589907	-0.8574889	1.5027653
C	-3.1818675	-1.0122580	-0.5696776
H	-3.8843962	-0.7920509	0.2450228
C	-0.5000430	-2.7982020	1.5733043
H	-1.4307585	-3.2000786	1.9945549
C	1.8328759	-0.0368280	-2.5798466
H	1.3166926	-0.4046124	-3.4982860
H	2.6321022	-0.7912660	-2.3739607
C	-1.7482744	-2.1234008	-1.9940696
H	-1.1837829	-2.9215486	-2.4957787
C	2.9111289	2.0062087	-1.5082228
H	3.4169307	2.9783972	-1.7060330
H	3.6695233	1.3496399	-1.0194155
C	0.3236333	-1.7924316	2.1676408
H	0.1381613	-1.2972772	3.1297419
C	-2.6463098	-2.2847766	-0.8885715
H	-2.8647720	-3.2253893	-0.3650160
C	-2.6426049	-0.0578082	-1.4971366
H	-2.8671245	1.0154767	-1.5199972
C	2.4659925	1.3530075	-2.8289693
H	3.3279114	1.2649391	-3.5288433
H	1.7162182	2.0113507	-3.3276806
C	-1.7795611	-0.7507483	-2.3898039
H	-1.2299018	-0.3099325	-3.2307900
F	-1.3721421	2.4475339	-0.0362407
F	0.1134765	3.0756369	4.3326096

vibrational spectrum

# mode	symmetry	wave number cm**(-1)	IR intensity km/mol	selection rules IR	RAMAN
1	a	-101.53	0.00000	YES	YES
2		-0.00	0.00000	-	-
3		0.00	0.00000	-	-
4		0.00	0.00000	-	-
5		0.00	0.00000	-	-
6		0.00	0.00000	-	-
7		0.00	0.00000	-	-
8	a	21.02	0.06988	YES	YES
9	a	26.55	0.01414	YES	YES
10	a	44.17	0.52708	YES	YES
11	a	51.23	0.72574	YES	YES
12	a	54.79	0.27448	YES	YES
13	a	79.79	0.98405	YES	YES
14	a	91.30	0.02798	YES	YES
15	a	109.05	1.71590	YES	YES
16	a	113.74	0.41951	YES	YES
17	a	128.78	0.50648	YES	YES
18	a	144.04	1.74145	YES	YES
19	a	150.25	1.44517	YES	YES
20	a	154.42	1.74030	YES	YES
21	a	167.71	4.03376	YES	YES
22	a	205.02	18.48378	YES	YES
23	a	218.76	1.79746	YES	YES
24	a	227.75	5.32370	YES	YES
25	a	230.63	0.89914	YES	YES
26	a	241.24	1.91749	YES	YES
27	a	251.14	1.21925	YES	YES
28	a	257.47	0.18974	YES	YES
29	a	261.83	0.84847	YES	YES
30	a	279.48	4.34956	YES	YES
31	a	289.01	5.06817	YES	YES
32	a	292.67	2.27033	YES	YES
33	a	296.74	11.28436	YES	YES
34	a	307.33	0.11864	YES	YES
35	a	316.33	23.13386	YES	YES
36	a	322.97	2.19723	YES	YES
37	a	351.43	18.15799	YES	YES
38	a	411.07	1.78558	YES	YES
39	a	417.77	10.91793	YES	YES
40	a	443.40	4.14634	YES	YES
41	a	461.98	0.56017	YES	YES
42	a	464.01	0.09137	YES	YES
43	a	510.70	13.82530	YES	YES
44	a	552.29	70.67992	YES	YES
45	a	561.40	78.63164	YES	YES
46	a	591.28	1.11462	YES	YES
47	a	593.48	1.69775	YES	YES
48	a	595.45	0.10485	YES	YES
49	a	597.65	0.82507	YES	YES
50	a	603.31	18.77759	YES	YES

B-F_1

SCF Energy (au) BP86/SV(P)	-1014.537487476	
SCF Energy (au) PBE0/def2-TZVPP	-1014.400645628	
SCF Energy (au) PBE0/def2-TZVPP	-1014.4164403536	(CH_2Cl_2 Correction)
SCF Energy (au) PBE0/def2-TZVPP	-1014.4085402076	(C_6H_6 Correction)
SCF Energy (au) PBE0/def2-TZVPP	-1014.4075382066	(C_6H_{12} Correction)
Zero Point Energy (au)	0.3631827	
Chemical Potential (kJ mol^{-1})	815.96	
Dispersion Correction (au) PBE0/def2-TZVPP	-0.06422355	

xyz coordinates
46

Zr	-0.6489998	-1.1837001	0.0160034
H	-0.4462969	3.2232235	4.0838482
H	-2.2730520	-0.4492931	2.6834086
N	-0.9657750	0.5083110	1.3427142
C	-0.6549568	2.5044106	3.2721175
C	0.0052107	2.6194525	2.0767788
H	0.7371928	3.4254038	1.9212634
C	-1.6667640	0.4630103	2.5207018
C	-1.5848138	1.4341132	3.4955836
H	-2.1554010	1.3421564	4.4314661
C	0.7316499	1.5060707	-0.1424821
C	0.0281987	-3.6494167	0.3666412
H	-0.4719982	-4.4336788	-0.2172369
C	1.5376908	2.7281211	-0.5640603
H	1.9332382	3.2617559	0.3291129
H	0.8345356	3.4480269	-1.0476151
C	-0.3430012	1.7390603	0.9252289
C	1.2280618	-2.9723969	0.0041758
H	1.8099198	-3.1352353	-0.9144584
C	0.8502084	0.2927417	-0.7630328
C	1.5529346	-2.0517467	1.0501585
H	2.4228826	-1.3836696	1.0767784
C	-3.1197811	-1.1850828	-0.6872273
H	-3.8793461	-1.1238370	0.1064159
C	-0.4048159	-3.1347250	1.6288930
H	-1.2918143	-3.4654146	2.1894707
C	1.8495320	0.0729900	-1.8874321
H	1.4311743	-0.6253483	-2.6510361
H	2.7454638	-0.4595962	-1.4792638
C	-1.5974248	-2.0147009	-2.2138234
H	-1.0014270	-2.7076875	-2.8250748
C	2.7095635	2.4038946	-1.5059091
H	3.0821020	3.3402265	-1.9786691
H	3.5593918	1.9908692	-0.9125463
C	0.5489409	-2.1555415	2.0555805
H	0.5079790	-1.5736897	2.9863393
C	-2.5563840	-2.3848667	-1.2196500
H	-2.8282792	-3.4092741	-0.9302265
C	-2.5172132	-0.0748780	-1.3509755
H	-2.6805604	0.9859393	-1.1213412
C	2.3042405	1.3765372	-2.5683981
H	3.1458203	1.1747265	-3.2696623
H	1.4666778	1.7869948	-3.1806187
C	-1.5796608	-0.5863626	-2.2969067
H	-0.9674006	0.0165901	-2.9792820
F	-1.3874434	2.5155157	0.1442477

vibrational spectrum

# mode	symmetry	wave number cm**(-1)	IR intensity km/mol	selection rules IR	RAMAN
1		-0.00	0.00000	-	-
2		-0.00	0.00000	-	-
3		-0.00	0.00000	-	-
4		0.00	0.00000	-	-
5		0.00	0.00000	-	-
6		0.00	0.00000	-	-
7	a	21.30	0.01194	YES	YES
8	a	30.98	0.02796	YES	YES
9	a	38.95	0.15733	YES	YES
10	a	54.34	0.33916	YES	YES
11	a	67.67	0.04404	YES	YES
12	a	80.78	0.30158	YES	YES
13	a	85.49	0.17878	YES	YES
14	a	110.69	0.30351	YES	YES
15	a	133.16	0.93381	YES	YES
16	a	142.64	2.10621	YES	YES
17	a	174.66	5.30391	YES	YES
18	a	181.60	5.81067	YES	YES
19	a	195.77	9.52206	YES	YES
20	a	214.88	2.67556	YES	YES
21	a	231.93	1.68389	YES	YES
22	a	243.13	11.30672	YES	YES
23	a	248.68	0.12907	YES	YES
24	a	261.66	0.47424	YES	YES
25	a	266.62	4.78979	YES	YES
26	a	285.22	0.58672	YES	YES
27	a	298.83	10.46536	YES	YES
28	a	328.39	15.45539	YES	YES
29	a	338.60	12.88475	YES	YES
30	a	367.94	14.96921	YES	YES
31	a	402.31	3.21509	YES	YES
32	a	428.10	19.05571	YES	YES
33	a	477.57	4.22659	YES	YES
34	a	482.84	3.39663	YES	YES
35	a	501.05	6.19387	YES	YES
36	a	540.23	42.63438	YES	YES
37	a	570.32	4.60364	YES	YES
38	a	589.13	0.32935	YES	YES
39	a	594.25	0.44894	YES	YES
40	a	597.72	0.21842	YES	YES
41	a	598.44	0.00538	YES	YES
42	a	644.68	13.40843	YES	YES
43	a	656.43	24.71647	YES	YES
44	a	700.49	1.57598	YES	YES
45	a	709.29	118.21191	YES	YES
46	a	740.49	6.35125	YES	YES
47	a	784.48	162.31793	YES	YES
48	a	786.35	79.81233	YES	YES
49	a	790.55	23.62093	YES	YES
50	a	792.49	54.11270	YES	YES

B-F_2

SCF Energy (au) BP86/SV(P)	-1113.720841777	
SCF Energy (au) PBE0/def2-TZVPP	-1113.605921010	
SCF Energy (au) PBE0/def2-TZVPP	-1113.6202357326	(CH_2Cl_2 Correction)
SCF Energy (au) PBE0/def2-TZVPP	-1113.6131472310	(C_6H_6 Correction)
SCF Energy (au) PBE0/def2-TZVPP	-1113.6122379287	(C_6H_{12} Correction)
Zero Point Energy (au)	0.3558759	
Chemical Potential (kJ mol^{-1})	796.54	
Dispersion Correction (au) PBE0/def2-TZVPP	-0.06489287	

xyz coordinates
46

Zr	-0.6968773	-1.1857787	0.0597286
H	-0.4304952	3.2332848	4.0988769
F	-2.3376974	-0.6347835	2.5964583
N	-0.9503687	0.5858987	1.3162488
C	-0.6292079	2.5253218	3.2757932
C	0.0184202	2.6791269	2.0772159
H	0.7292897	3.5051014	1.9297700
C	-1.6185390	0.5333516	2.4809427
C	-1.5517433	1.4424160	3.5041940
H	-2.1219724	1.3152152	4.4336166
C	0.7367217	1.5440177	-0.1499798
C	0.0761027	-3.6319838	0.3271048
H	-0.3831530	-4.4137922	-0.2927757
C	1.5754754	2.7331500	-0.5932895
H	1.9841955	3.2712344	0.2920692
H	0.8959101	3.4652703	-1.0925909
C	-0.3308300	1.8192662	0.9064099
C	1.2500376	-2.8906047	0.0047532
H	1.8521069	-2.9983045	-0.9085146
C	0.8105477	0.3098167	-0.7332758
C	1.5235428	-1.9978567	1.0886644
H	2.3637341	-1.2949158	1.1510129
C	-3.1503411	-1.2489858	-0.6858336
H	-3.9177921	-1.2467083	0.1018492
C	-0.3968422	-3.1809263	1.5967381
H	-1.2824094	-3.5568427	2.1290424
C	1.8034786	0.0373592	-1.8535798
H	1.3672427	-0.6668592	-2.6017138
H	2.6864171	-0.5080623	-1.4345240
C	-1.5667639	-1.9634270	-2.2085379
H	-0.9358199	-2.6105481	-2.8346877
C	2.7351231	2.3521323	-1.5289300
H	3.1352453	3.2642367	-2.0257394
H	3.5733624	1.9268035	-0.9278965
C	0.5078643	-2.1754643	2.0714578
H	0.4327153	-1.6357295	3.0252917
C	-2.5307147	-2.4054614	-1.2475873
H	-2.7685540	-3.4485577	-0.9981319
C	-2.5761653	-0.0929215	-1.2957074
H	-2.7916884	0.9531607	-1.0431776
C	2.2929246	1.3124659	-2.5649887
H	3.1242821	1.0694906	-3.2654160
H	1.4649930	1.7340187	-3.1828848
C	-1.6036230	-0.5336466	-2.2422012
H	-1.0051856	0.1166964	-2.8920520
F	-1.3629489	2.5933250	0.1367775

vibrational spectrum

# mode	symmetry	wave number cm**(-1)	IR intensity km/mol	selection rules IR	RAMAN
1		-0.00	0.00000	-	-
2		0.00	0.00000	-	-
3		0.00	0.00000	-	-
4		0.00	0.00000	-	-
5		0.00	0.00000	-	-
6		0.00	0.00000	-	-
7	a	30.36	0.02701	YES	YES
8	a	37.06	0.04947	YES	YES
9	a	43.77	0.17586	YES	YES
10	a	56.88	0.13136	YES	YES
11	a	68.19	0.01027	YES	YES
12	a	88.07	0.22126	YES	YES
13	a	93.14	0.18912	YES	YES
14	a	101.01	2.30594	YES	YES
15	a	113.34	2.15716	YES	YES
16	a	142.54	0.44787	YES	YES
17	a	152.96	1.10859	YES	YES
18	a	180.82	5.36976	YES	YES
19	a	192.23	12.42106	YES	YES
20	a	198.99	1.68384	YES	YES
21	a	220.16	1.01332	YES	YES
22	a	237.30	2.21783	YES	YES
23	a	248.28	9.34982	YES	YES
24	a	251.56	0.17342	YES	YES
25	a	267.66	1.38336	YES	YES
26	a	269.93	3.81682	YES	YES
27	a	299.04	4.94564	YES	YES
28	a	309.20	2.93818	YES	YES
29	a	326.20	9.03974	YES	YES
30	a	334.13	18.22724	YES	YES
31	a	379.23	8.37435	YES	YES
32	a	389.50	11.64550	YES	YES
33	a	450.05	5.28417	YES	YES
34	a	480.16	0.79101	YES	YES
35	a	487.99	0.99413	YES	YES
36	a	497.54	4.50452	YES	YES
37	a	525.67	19.68415	YES	YES
38	a	567.09	19.22316	YES	YES
39	a	577.53	4.63310	YES	YES
40	a	589.38	0.37944	YES	YES
41	a	594.53	0.56028	YES	YES
42	a	598.44	0.15615	YES	YES
43	a	599.06	0.21261	YES	YES
44	a	618.87	15.26739	YES	YES
45	a	663.34	27.78177	YES	YES
46	a	685.47	20.70977	YES	YES
47	a	718.18	81.09649	YES	YES
48	a	733.77	9.37092	YES	YES
49	a	777.69	123.38243	YES	YES
50	a	788.86	115.40738	YES	YES

B-F_3

SCF Energy (au) BP86/SV(P)	-1212.891969484	
SCF Energy (au) PBE0/def2-TZVPP	-1212.800579725	
SCF Energy (au) PBE0/def2-TZVPP	-1212.8148894680	(CH_2Cl_2 Correction)
SCF Energy (au) PBE0/def2-TZVPP	-1212.8078085373	(C_6H_6 Correction)
SCF Energy (au) PBE0/def2-TZVPP	-1212.8068996403	(C_6H_{12} Correction)
Zero Point Energy (au)	0.3480648	
Chemical Potential (kJ mol^{-1})	772.82	
Dispersion Correction (au) PBE0/def2-TZVPP	-0.06507800	

xyz coordinates
46

Zr	-0.6950309	-1.1919341	0.0508554
F	-0.4131267	3.3818187	4.2656064
F	-2.3403873	-0.6247372	2.6192793
N	-0.9526702	0.5771876	1.3192192
C	-0.6323175	2.5226058	3.2469462
C	0.0342128	2.6753186	2.0632161
H	0.7376723	3.5089854	1.9384453
C	-1.6162738	0.5323314	2.4840550
C	-1.5540013	1.4515157	3.5005273
H	-2.1142588	1.3579081	4.4383994
C	0.7345635	1.5385032	-0.1527881
C	0.0722732	-3.6369803	0.3176848
H	-0.3885312	-4.4181749	-0.3017353
C	1.5724612	2.7289065	-0.5935718
H	1.9794571	3.2659161	0.2930579
H	0.8931996	3.4608887	-1.0934548
C	-0.3321487	1.8139942	0.9041875
C	1.2473681	-2.8975103	-0.0055785
H	1.8481228	-3.0059133	-0.9196583
C	0.8094725	0.3051964	-0.7377270
C	1.5240890	-2.0061865	1.0785246
H	2.3662927	-1.3057440	1.1411198
C	-3.1498075	-1.2558107	-0.6880931
H	-3.9168257	-1.2544938	0.1001415
C	-0.3979050	-3.1862939	1.5885154
H	-1.2828022	-3.5620251	2.1221657
C	1.8034693	0.0353629	-1.8577759
H	1.3680288	-0.6674181	-2.6076289
H	2.6855643	-0.5107525	-1.4381247
C	-1.5681024	-1.9684407	-2.2135525
H	-0.9383472	-2.6149849	-2.8414413
C	2.7338473	2.3498014	-1.5277467
H	3.1342041	3.2629428	-2.0222325
H	3.5711421	1.9241520	-0.9257863
C	0.5092872	-2.1828349	2.0625061
H	0.4382653	-1.6456316	3.0180874
C	-2.5305163	-2.4116093	-1.2514235
H	-2.7675922	-3.4550034	-1.0025982
C	-2.5775840	-0.0991287	-1.2988399
H	-2.7935852	0.9467975	-1.0458594
C	2.2938350	1.3116781	-2.5661905
H	3.1265562	1.0695745	-3.2651673
H	1.4671772	1.7338005	-3.1853190
C	-1.6064449	-0.5386518	-2.2471802
H	-1.0096464	0.1122359	-2.8979886
F	-1.3726560	2.5728378	0.1349219

vibrational spectrum

# mode	symmetry	wave number cm**(-1)	IR intensity km/mol	selection rules IR	RAMAN
1		-0.00	0.00000	-	-
2		-0.00	0.00000	-	-
3		-0.00	0.00000	-	-
4		-0.00	0.00000	-	-
5		0.00	0.00000	-	-
6		0.00	0.00000	-	-
7	a	29.82	0.02326	YES	YES
8	a	35.61	0.05606	YES	YES
9	a	38.02	0.08134	YES	YES
10	a	54.73	0.05757	YES	YES
11	a	65.89	0.02973	YES	YES
12	a	86.85	0.10643	YES	YES
13	a	91.53	0.12022	YES	YES
14	a	97.78	1.44224	YES	YES
15	a	114.10	1.73214	YES	YES
16	a	132.11	1.50662	YES	YES
17	a	142.41	0.46055	YES	YES
18	a	176.71	6.25742	YES	YES
19	a	189.26	10.43894	YES	YES
20	a	195.53	2.77729	YES	YES
21	a	200.01	0.41545	YES	YES
22	a	217.57	2.37841	YES	YES
23	a	237.90	2.22910	YES	YES
24	a	248.49	9.37305	YES	YES
25	a	251.00	0.19388	YES	YES
26	a	261.69	1.33545	YES	YES
27	a	270.59	3.56524	YES	YES
28	a	303.04	9.02008	YES	YES
29	a	314.40	4.31773	YES	YES
30	a	333.15	15.86639	YES	YES
31	a	347.04	0.79776	YES	YES
32	a	360.65	6.31621	YES	YES
33	a	382.04	6.68945	YES	YES
34	a	398.73	5.63473	YES	YES
35	a	458.44	16.52468	YES	YES
36	a	481.20	0.98686	YES	YES
37	a	502.21	8.41392	YES	YES
38	a	519.26	10.15165	YES	YES
39	a	542.97	2.27153	YES	YES
40	a	567.80	18.84646	YES	YES
41	a	581.46	2.14134	YES	YES
42	a	589.29	0.37397	YES	YES
43	a	594.20	0.69765	YES	YES
44	a	598.33	0.09523	YES	YES
45	a	598.80	0.05095	YES	YES
46	a	618.87	2.81120	YES	YES
47	a	632.44	46.00318	YES	YES
48	a	686.38	24.12826	YES	YES
49	a	713.06	14.21208	YES	YES
50	a	734.13	29.57312	YES	YES

B-F_4

SCF Energy (au) BP86/SV(P) -1312.042640290
SCF Energy (au) PBE0/def2-TZVPP -1311.969507684
SCF Energy (au) PBE0/def2-TZVPP -1311.9831616018 (CH_2Cl_2 Correction)
SCF Energy (au) PBE0/def2-TZVPP -1311.9764007975 (C_6H_6 Correction)
SCF Energy (au) PBE0/def2-TZVPP -1311.9755335936 (C_6H_{12} Correction)
Zero Point Energy (au) 0.3402639
Chemical Potential (kJ mol^{-1}) 747.88
Dispersion Correction (au) PBE0/def2-TZVPP -0.06553122

xyz coordinates
46

Zr	-0.6962389	-1.1765099	0.0701957
H	-0.3872729	3.1004205	4.2091172
F	-2.4201907	-0.6266791	2.4836607
N	-0.9430245	0.6167848	1.3000718
C	-0.5948897	2.4331958	3.3583513
C	0.0645013	2.6043696	2.1728167
F	0.9960265	3.5835777	2.0775906
C	-1.6615636	0.5159307	2.4409239
C	-1.5501862	1.3741433	3.5013477
F	-2.2731399	1.2432027	4.6356121
C	0.7590409	1.5656231	-0.1458564
C	0.0597627	-3.6233339	0.3681566
H	-0.3926620	-4.4092783	-0.2514535
C	1.5471254	2.7570357	-0.6756402
H	1.9571365	3.3569517	0.1611553
H	0.8232852	3.4339739	-1.1900207
C	-0.2955739	1.8378489	0.9279657
C	1.2408672	-2.8884999	0.0566466
H	1.8563709	-3.0065947	-0.8463523
C	0.8271778	0.3164804	-0.6981417
C	1.5025350	-1.9875985	1.1368735
H	2.3465272	-1.2900214	1.2068641
C	-3.1299273	-1.2462397	-0.7431314
H	-3.9196636	-1.2546149	0.0220514
C	-0.4299204	-3.1599696	1.6268292
H	-1.3241180	-3.5286570	2.1497296
C	1.8026930	0.0187809	-1.8279687
H	1.3750463	-0.7353577	-2.5305825
H	2.7080687	-0.4789329	-1.3980391
C	-1.5009190	-1.9399086	-2.2271365
H	-0.8514276	-2.5786944	-2.8426651
C	2.6838667	2.3641535	-1.6332144
H	3.0402592	3.2655218	-2.1802767
H	3.5538221	1.9901062	-1.0432597
C	0.4715057	-2.1533634	2.1059112
H	0.3855226	-1.6071673	3.0554230
C	-2.4881886	-2.3945825	-1.2961665
H	-2.7277735	-3.4407328	-1.0618760
C	-2.5461445	-0.0823326	-1.3288561
H	-2.7835398	0.9606049	-1.0822472
C	2.2418420	1.2699172	-2.6101232
H	3.0598731	1.0130639	-3.3211329
H	1.3882063	1.6430576	-3.2245024
C	-1.5464753	-0.5101967	-2.2521775
H	-0.9364907	0.1482816	-2.8827228
F	-1.2917320	2.7062396	0.2262495

vibrational spectrum

# mode	symmetry	wave number cm**(-1)	IR intensity km/mol	selection rules IR	RAMAN
1		-0.00	0.00000	-	-
2		0.00	0.00000	-	-
3		0.00	0.00000	-	-
4		0.00	0.00000	-	-
5		0.00	0.00000	-	-
6		0.00	0.00000	-	-
7	a	23.76	0.23095	YES	YES
8	a	31.65	0.01622	YES	YES
9	a	35.96	0.06220	YES	YES
10	a	44.92	0.07951	YES	YES
11	a	56.87	0.31223	YES	YES
12	a	72.22	0.19602	YES	YES
13	a	91.95	0.10882	YES	YES
14	a	98.03	1.18041	YES	YES
15	a	110.11	1.55805	YES	YES
16	a	122.66	1.20053	YES	YES
17	a	146.29	0.77345	YES	YES
18	a	163.10	0.70390	YES	YES
19	a	176.26	3.52452	YES	YES
20	a	191.86	10.07771	YES	YES
21	a	198.15	4.83818	YES	YES
22	a	224.79	1.26801	YES	YES
23	a	236.88	1.99060	YES	YES
24	a	246.57	2.61367	YES	YES
25	a	248.75	8.79839	YES	YES
26	a	254.46	0.32998	YES	YES
27	a	266.00	0.88792	YES	YES
28	a	272.61	1.28015	YES	YES
29	a	274.48	4.39864	YES	YES
30	a	300.24	9.02368	YES	YES
31	a	316.45	0.78776	YES	YES
32	a	327.67	10.93087	YES	YES
33	a	336.07	15.72454	YES	YES
34	a	364.44	5.71205	YES	YES
35	a	383.51	6.65346	YES	YES
36	a	401.42	0.58052	YES	YES
37	a	446.69	1.24157	YES	YES
38	a	476.18	5.23078	YES	YES
39	a	477.74	3.39617	YES	YES
40	a	490.51	3.62103	YES	YES
41	a	541.10	22.75002	YES	YES
42	a	567.92	27.10748	YES	YES
43	a	588.96	0.46654	YES	YES
44	a	593.69	14.76767	YES	YES
45	a	594.86	8.47400	YES	YES
46	a	596.31	4.59263	YES	YES
47	a	598.78	0.25135	YES	YES
48	a	600.06	10.47699	YES	YES
49	a	655.88	3.56989	YES	YES
50	a	716.35	64.05144	YES	YES

B-F_5

SCF Energy (au) BP86/SV(P) -1411.200002083
SCF Energy (au) PBE0/def2-TZVPP -1411.150408616
SCF Energy (au) PBE0/def2-TZVPP -1411.1638207327 (CH_2Cl_2 Correction)
SCF Energy (au) PBE0/def2-TZVPP -1411.1571597434 (C_6H_6 Correction)
SCF Energy (au) PBE0/def2-TZVPP -1411.1563083108 (C_6H_{12} Correction)
Zero Point Energy (au) 0.3325507
Chemical Potential (kJ mol^{-1}) 724.02
Dispersion Correction (au) PBE0/def2-TZVPP -0.06575765

xyz coordinates
46

Zr	-0.6920019	-1.1829691	0.0606765
F	-0.3530430	3.2248974	4.3936539
F	-2.4342070	-0.6155301	2.4917241
N	-0.9449040	0.6085610	1.3011741
C	-0.5908500	2.4288521	3.3413796
C	0.0910231	2.5934576	2.1621437
F	1.0197111	3.5744161	2.0885178
C	-1.6621576	0.5137611	2.4394515
C	-1.5568737	1.3722517	3.5011532
F	-2.2872834	1.2746450	4.6277371
C	0.7564158	1.5612431	-0.1489968
C	0.0554611	-3.6285912	0.3649649
H	-0.3988725	-4.4151037	-0.2524665
C	1.5395470	2.7555492	-0.6796845
H	1.9473522	3.3582198	0.1561842
H	0.8126153	3.4286088	-1.1947646
C	-0.2951105	1.8333498	0.9279263
C	1.2384219	-2.8972943	0.0512270
H	1.8531909	-3.0191941	-0.8518039
C	0.8276143	0.3122642	-0.7011688
C	1.5032809	-1.9951063	1.1295032
H	2.3495193	-1.3001627	1.1984727
C	-3.1276150	-1.2487387	-0.7479063
H	-3.9172273	-1.2557443	0.0174745
C	-0.4317427	-3.1621062	1.6234009
H	-1.3260800	-3.5283594	2.1478386
C	1.8044077	0.0177010	-1.8309331
H	1.3797525	-0.7382739	-2.5333128
H	2.7111162	-0.4765406	-1.3999543
C	-1.5012990	-1.9454348	-2.2334120
H	-0.8537990	-2.5856197	-2.8496057
C	2.6776231	2.3663515	-1.6371244
H	3.0303118	3.2688117	-2.1845963
H	3.5489882	1.9961410	-1.0469743
C	0.4723425	-2.1566281	2.0996202
H	0.3901219	-1.6101791	3.0493320
C	-2.4880975	-2.3982914	-1.3008778
H	-2.7290318	-3.4440269	-1.0662076
C	-2.5432519	-0.0860179	-1.3355319
H	-2.7794126	0.9574727	-1.0898852
C	2.2394972	1.2700397	-2.6134075
H	3.0586066	1.0152498	-3.3238092
H	1.3849460	1.6398138	-3.2285219
C	-1.5455445	-0.5157047	-2.2600094
H	-0.9356401	0.1414258	-2.8920618
F	-1.2978216	2.6925334	0.2294605

vibrational spectrum

# mode	symmetry	wave number	IR intensity	selection rules	
#		cm**(-1)	km/mol	IR	RAMAN

1		-0.00	0.00000	-	-
2		-0.00	0.00000	-	-
3		-0.00	0.00000	-	-
4		-0.00	0.00000	-	-
5		0.00	0.00000	-	-
6		0.00	0.00000	-	-
7	a	21.00	0.22653	YES	YES
8	a	30.72	0.01230	YES	YES
9	a	35.39	0.06358	YES	YES
10	a	43.76	0.12144	YES	YES
11	a	55.84	0.30394	YES	YES
12	a	70.77	0.16560	YES	YES
13	a	90.11	0.14989	YES	YES
14	a	97.53	0.75049	YES	YES
15	a	110.33	0.94250	YES	YES
16	a	112.39	1.21416	YES	YES
17	a	141.77	1.05711	YES	YES
18	a	148.45	0.38972	YES	YES
19	a	158.02	1.39513	YES	YES
20	a	176.86	4.47414	YES	YES
21	a	190.12	10.21309	YES	YES
22	a	204.08	3.41576	YES	YES
23	a	219.35	1.49793	YES	YES
24	a	237.54	2.21858	YES	YES
25	a	248.72	9.87829	YES	YES
26	a	249.52	0.76230	YES	YES
27	a	253.96	0.42329	YES	YES
28	a	257.35	0.68357	YES	YES
29	a	268.77	2.59403	YES	YES
30	a	283.01	3.82779	YES	YES
31	a	297.93	0.02769	YES	YES
32	a	301.11	7.22404	YES	YES
33	a	317.90	0.64136	YES	YES
34	a	328.84	13.02809	YES	YES
35	a	337.38	14.32548	YES	YES
36	a	352.60	3.74700	YES	YES
37	a	389.66	4.68996	YES	YES
38	a	416.18	0.89980	YES	YES
39	a	442.95	5.72994	YES	YES
40	a	471.07	5.21866	YES	YES
41	a	482.00	5.52119	YES	YES
42	a	502.68	15.84751	YES	YES
43	a	518.94	12.47929	YES	YES
44	a	564.91	9.53422	YES	YES
45	a	584.22	13.21433	YES	YES
46	a	589.05	1.22786	YES	YES
47	a	594.27	0.46081	YES	YES
48	a	597.43	0.11793	YES	YES
49	a	598.57	0.34557	YES	YES
50	a	610.15	13.37784	YES	YES

C-F1

SCF Energy (au) BP86/SV(P)	-1014.624567931	
SCF Energy (au) PBE0/def2-TZVPP	-1014.483718428	
SCF Energy (au) PBE0/def2-TZVPP	-1014.4972651946	(CH_2Cl_2 Correction)
SCF Energy (au) PBE0/def2-TZVPP	-1014.4905623632	(C_6H_6 Correction)
SCF Energy (au) PBE0/def2-TZVPP	-1014.4897019168	(C_6H_{12} Correction)
Zero Point Energy (au)	0.3651223	
Chemical Potential (kJ mol^{-1})	823.78	
Dispersion Correction (au) PBE0/def2-TZVPP	-0.06400448	

xyz coordinates
46

Zr	-0.2485412	-1.3377647	-0.5771007
F	0.9345420	-1.7667909	-2.1411965
H	-1.8672538	2.9931087	3.8528596
H	-2.3925079	-0.9169995	2.0395760
N	-0.9189233	0.2859553	1.1991978
C	-1.6006864	2.2241656	3.1086709
C	-0.5868272	2.4653414	2.1792102
H	-0.0528412	3.4260722	2.1841599
C	-1.8903071	0.0627362	2.1040016
C	-2.2731185	0.9884471	3.0812204
H	-3.0761390	0.7443879	3.7933689
C	0.8083717	1.6546060	0.2112438
C	-0.4557737	-2.9754733	1.4347704
H	-1.4248221	-3.1125428	1.9342573
C	1.6182384	2.9483107	0.2439057
H	1.9233549	3.1827952	1.2907703
H	0.9737204	3.8052092	-0.0752544
C	-0.2410944	1.4757281	1.2167499
C	0.0362847	-3.7528637	0.3329900
H	-0.5020134	-4.5630470	-0.1778477
C	0.9870105	0.6458776	-0.7093957
C	1.3566905	-3.3180239	0.0440469
H	1.9856446	-3.6808826	-0.7781417
C	-2.5866198	-0.2319189	-1.0652755
H	-2.9027433	0.6443095	-0.4864378
C	0.5725969	-2.0680844	1.8169600
H	0.5309140	-1.3508492	2.6456138
C	2.0175474	0.8190028	-1.8049066
H	1.6151257	0.4094352	-2.7572670
H	2.8724251	0.1353022	-1.5752433
C	-2.2786820	-2.4041160	-1.7828892
H	-2.3232376	-3.5000895	-1.8451266
C	2.8703715	2.8881182	-0.6453149
H	3.2977628	3.9085593	-0.7672826
H	3.6534768	2.2738553	-0.1411368
C	1.6807226	-2.2509695	0.9331785
H	2.6284907	-1.6973178	0.9636708
C	-2.8300071	-1.5945663	-0.7334131
H	-3.3983416	-1.9651466	0.1307959
C	-1.8554916	-0.1949195	-2.2929650
H	-1.5117414	0.7096233	-2.8118731
C	2.5347062	2.2550450	-1.9995552
H	3.4233485	2.2562032	-2.6707251
H	1.7531757	2.8687803	-2.5079516
C	-1.7019305	-1.5371922	-2.7487787
H	-1.1548775	-1.8514171	-3.6461400

vibrational spectrum

# mode	symmetry	wave number cm**(-1)	IR intensity km/mol	selection rules IR	RAMAN
1		-0.00	0.00000	-	-
2		-0.00	0.00000	-	-
3		0.00	0.00000	-	-
4		0.00	0.00000	-	-
5		0.00	0.00000	-	-
6		0.00	0.00000	-	-
7	a	31.73	0.25192	YES	YES
8	a	38.64	0.26942	YES	YES
9	a	42.85	0.09980	YES	YES
10	a	47.94	0.02432	YES	YES
11	a	84.95	0.65565	YES	YES
12	a	91.73	0.68976	YES	YES
13	a	97.89	0.48804	YES	YES
14	a	128.88	0.48852	YES	YES
15	a	147.38	0.47265	YES	YES
16	a	153.26	0.61195	YES	YES
17	a	186.66	0.10790	YES	YES
18	a	191.04	0.28797	YES	YES
19	a	210.40	1.35989	YES	YES
20	a	212.51	3.66170	YES	YES
21	a	227.28	1.43651	YES	YES
22	a	233.61	3.80134	YES	YES
23	a	236.57	5.29216	YES	YES
24	a	256.84	0.10764	YES	YES
25	a	263.47	0.06304	YES	YES
26	a	272.27	7.80064	YES	YES
27	a	288.27	13.23472	YES	YES
28	a	297.40	4.44437	YES	YES
29	a	325.18	30.07247	YES	YES
30	a	338.93	0.95951	YES	YES
31	a	428.22	1.71203	YES	YES
32	a	442.70	1.06964	YES	YES
33	a	450.28	1.19373	YES	YES
34	a	479.21	3.10117	YES	YES
35	a	526.60	14.43068	YES	YES
36	a	529.17	41.20877	YES	YES
37	a	566.29	2.79945	YES	YES
38	a	590.95	0.45058	YES	YES
39	a	596.88	0.74469	YES	YES
40	a	597.56	2.94642	YES	YES
41	a	601.07	2.55519	YES	YES
42	a	627.23	2.51025	YES	YES
43	a	669.45	3.11864	YES	YES
44	a	738.46	31.31122	YES	YES
45	a	762.36	8.26769	YES	YES
46	a	776.03	70.09864	YES	YES
47	a	780.35	28.75106	YES	YES
48	a	783.36	108.17229	YES	YES
49	a	785.46	10.42729	YES	YES
50	a	787.96	59.85534	YES	YES

C-F2

SCF Energy (au) BP86/SV(P)	-1113.799782527	
SCF Energy (au) PBE0/def2-TZVPP	-1113.681271205	
SCF Energy (au) PBE0/def2-TZVPP	-1113.6939685217	(CH_2Cl_2 Correction)
SCF Energy (au) PBE0/def2-TZVPP	-1113.6877236568	(C_6H_6 Correction)
SCF Energy (au) PBE0/def2-TZVPP	-1113.6869164224	(C_6H_{12} Correction)
Zero Point Energy (au)	0.3571624	
Chemical Potential (kJ mol^{-1})	799.93	
Dispersion Correction (au) PBE0/def2-TZVPP	-0.06404460	

xyz coordinates
46

Zr	-0.2808389	-1.3561181	-0.5492469
F	0.9008976	-1.8102840	-2.1034492
H	-1.7369761	3.1547058	3.8492730
F	-2.5586246	-0.9473283	2.1428195
N	-0.9294205	0.3524382	1.2430183
C	-1.5089653	2.3618915	3.1178357
C	-0.4972164	2.5436512	2.1703488
H	0.0722284	3.4822718	2.1491809
C	-1.8800634	0.2165101	2.1619366
C	-2.2347367	1.1604288	3.1315742
H	-3.0418386	0.9452981	3.8460419
C	0.8295599	1.6488028	0.2011913
C	-0.5094698	-3.1406870	1.3259266
H	-1.5101803	-3.3972101	1.6964513
C	1.6734820	2.9219875	0.2006213
H	2.0135355	3.1541761	1.2370143
H	1.0413087	3.7921868	-0.1059548
C	-0.2049462	1.5228793	1.2257692
C	0.1827630	-3.7795213	0.2431221
H	-0.2048141	-4.5957818	-0.3821575
C	0.9648507	0.6233395	-0.7092736
C	1.4707120	-3.1901489	0.1362643
H	2.2201811	-3.4260691	-0.6291689
C	-2.6923078	-0.3848303	-0.9409658
H	-3.1089335	0.3714124	-0.2657179
C	0.3592392	-2.1575480	1.8727546
H	0.1541358	-1.5230415	2.7428498
C	1.9740134	0.7638988	-1.8293816
H	1.5446391	0.3476076	-2.7666980
H	2.8205559	0.0676077	-1.6074889
C	-2.1873694	-2.3982395	-1.9452585
H	-2.1250170	-3.4749326	-2.1556822
C	2.8989981	2.8271584	-0.7214914
H	3.3420722	3.8377900	-0.8662382
H	3.6837941	2.2026666	-0.2327600
C	1.5728900	-2.1562384	1.1115080
H	2.4468894	-1.5157535	1.2899118
C	-2.8253612	-1.7934489	-0.8104237
H	-3.3609525	-2.3212550	-0.0110977
C	-1.9447796	-0.1118076	-2.1322336
H	-1.6823204	0.8830343	-2.5158249
C	2.5128025	2.1864069	-2.0578728
H	3.3815607	2.1611106	-2.7541048
H	1.7287996	2.8088157	-2.5517501
C	-1.6754639	-1.3586607	-2.7673145
H	-1.0893127	-1.4991719	-3.6838581

vibrational spectrum

# mode	symmetry	wave number cm**(-1)	IR intensity km/mol	selection rules IR	RAMAN
1		-0.00	0.00000	-	-
2		-0.00	0.00000	-	-
3		-0.00	0.00000	-	-
4		-0.00	0.00000	-	-
5		0.00	0.00000	-	-
6		0.00	0.00000	-	-
7	a	30.50	0.06886	YES	YES
8	a	36.56	0.02915	YES	YES
9	a	41.46	0.07568	YES	YES
10	a	46.36	0.62555	YES	YES
11	a	81.98	0.94198	YES	YES
12	a	87.84	0.94919	YES	YES
13	a	95.15	0.71241	YES	YES
14	a	111.70	1.64843	YES	YES
15	a	132.76	0.07731	YES	YES
16	a	143.99	0.29623	YES	YES
17	a	179.48	0.07109	YES	YES
18	a	186.89	0.33102	YES	YES
19	a	202.35	0.28844	YES	YES
20	a	210.93	2.11986	YES	YES
21	a	217.36	11.28731	YES	YES
22	a	220.86	0.86498	YES	YES
23	a	229.27	1.43179	YES	YES
24	a	252.69	0.04317	YES	YES
25	a	254.03	0.18749	YES	YES
26	a	270.45	0.90173	YES	YES
27	a	277.22	5.38177	YES	YES
28	a	286.27	7.99787	YES	YES
29	a	300.71	22.80202	YES	YES
30	a	325.75	2.98854	YES	YES
31	a	337.39	12.90394	YES	YES
32	a	391.37	3.51020	YES	YES
33	a	441.99	0.62249	YES	YES
34	a	470.94	3.28022	YES	YES
35	a	485.14	1.38726	YES	YES
36	a	495.24	2.10801	YES	YES
37	a	528.93	52.04467	YES	YES
38	a	540.04	0.94142	YES	YES
39	a	562.35	0.90092	YES	YES
40	a	590.51	0.00810	YES	YES
41	a	596.18	0.77323	YES	YES
42	a	597.82	2.25931	YES	YES
43	a	601.25	2.41503	YES	YES
44	a	646.56	4.05061	YES	YES
45	a	655.67	0.66318	YES	YES
46	a	728.11	13.61497	YES	YES
47	a	745.42	10.59866	YES	YES
48	a	777.85	12.23189	YES	YES
49	a	782.00	192.92097	YES	YES
50	a	785.64	13.21535	YES	YES

C-F_3

SCF Energy (au) BP86/SV(P)	-1212.970134290	
SCF Energy (au) PBE0/def2-TZVPP	-1212.874791258	
SCF Energy (au) PBE0/def2-TZVPP	-1212.8868982252	(CH_2Cl_2 Correction)
SCF Energy (au) PBE0/def2-TZVPP	-1212.8809726815	(C_6H_6 Correction)
SCF Energy (au) PBE0/def2-TZVPP	-1212.8802024829	(C_6H_{12} Correction)
Zero Point Energy (au)	0.3492332	
Chemical Potential (kJ mol^{-1})	775.97	
Dispersion Correction (au) PBE0/def2-TZVPP	-0.06423972	

xyz coordinates
46

Zr	-0.2770061	-1.3624886	-0.5553411
F	0.8931106	-1.8053803	-2.1202324
F	-1.8129879	3.3180847	3.9613971
F	-2.5293507	-0.9678060	2.1686135
N	-0.9173642	0.3379090	1.2474357
C	-1.5179616	2.3498139	3.0847966
C	-0.5031811	2.5480846	2.1490883
H	0.0419680	3.5002272	2.1395642
C	-1.8659514	0.2022701	2.1681475
C	-2.2395859	1.1493388	3.1268778
H	-3.0411507	0.9583190	3.8524022
C	0.8303364	1.6461141	0.2004726
C	-0.5011764	-3.1604628	1.3079185
H	-1.4995254	-3.4249975	1.6796869
C	1.6671542	2.9232352	0.2000780
H	1.9995937	3.1610314	1.2376877
H	1.0324983	3.7888728	-0.1142801
C	-0.2053989	1.5169275	1.2210489
C	0.1900676	-3.7874328	0.2173939
H	-0.1971828	-4.5992162	-0.4137977
C	0.9711235	0.6180841	-0.7066879
C	1.4767027	-3.1946501	0.1131744
H	2.2250651	-3.4235517	-0.6554525
C	-2.6739250	-0.3607046	-0.9449158
H	-3.0695000	0.4144635	-0.2778844
C	0.3668908	-2.1809962	1.8617100
H	0.1626240	-1.5553415	2.7384181
C	1.9831283	0.7605293	-1.8231540
H	1.5585491	0.3405180	-2.7608734
H	2.8302934	0.0662287	-1.5967746
C	-2.2116862	-2.3987030	-1.9198312
H	-2.1714759	-3.4795995	-2.1134968
C	2.8980607	2.8290479	-0.7149302
H	3.3397073	3.8402117	-0.8598971
H	3.6811402	2.2075351	-0.2199628
C	1.5787712	-2.1694882	1.0977546
H	2.4523241	-1.5299920	1.2816671
C	-2.8284808	-1.7649334	-0.7890767
H	-3.3650816	-2.2709523	0.0233699
C	-1.9333309	-0.1202806	-2.1472569
H	-1.6580292	0.8632330	-2.5504067
C	2.5198510	2.1838372	-2.0515447
H	3.3916713	2.1589898	-2.7437055
H	1.7372561	2.8032082	-2.5510845
C	-1.6908891	-1.3817678	-2.7638396
H	-1.1176655	-1.5473699	-3.6842766

vibrational spectrum

# mode	symmetry	wave number cm**(-1)	IR intensity km/mol	selection rules IR	RAMAN
1		-0.00	0.00000	-	-
2		-0.00	0.00000	-	-
3		0.00	0.00000	-	-
4		0.00	0.00000	-	-
5		0.00	0.00000	-	-
6		0.00	0.00000	-	-
7	a	29.05	0.24455	YES	YES
8	a	34.61	0.00515	YES	YES
9	a	40.86	0.07312	YES	YES
10	a	43.97	0.40713	YES	YES
11	a	72.89	0.16338	YES	YES
12	a	86.36	1.05233	YES	YES
13	a	95.16	0.89605	YES	YES
14	a	105.67	1.26825	YES	YES
15	a	129.64	0.30041	YES	YES
16	a	143.72	0.28786	YES	YES
17	a	169.64	0.38824	YES	YES
18	a	181.79	0.10099	YES	YES
19	a	188.03	0.49983	YES	YES
20	a	207.34	0.80804	YES	YES
21	a	212.48	1.51615	YES	YES
22	a	216.47	11.36513	YES	YES
23	a	225.03	0.87195	YES	YES
24	a	229.54	1.53613	YES	YES
25	a	252.05	0.05019	YES	YES
26	a	263.01	0.47193	YES	YES
27	a	270.41	2.86959	YES	YES
28	a	280.16	3.82354	YES	YES
29	a	289.69	6.34272	YES	YES
30	a	308.94	11.65896	YES	YES
31	a	315.17	20.57413	YES	YES
32	a	341.06	6.34158	YES	YES
33	a	361.89	1.27847	YES	YES
34	a	381.78	2.72699	YES	YES
35	a	452.77	1.50926	YES	YES
36	a	480.01	3.19264	YES	YES
37	a	509.81	3.56914	YES	YES
38	a	521.60	1.90412	YES	YES
39	a	530.63	55.11346	YES	YES
40	a	549.21	0.63114	YES	YES
41	a	590.33	0.01667	YES	YES
42	a	596.05	0.75282	YES	YES
43	a	597.62	2.30139	YES	YES
44	a	600.29	1.29754	YES	YES
45	a	602.14	1.31122	YES	YES
46	a	619.22	3.46385	YES	YES
47	a	644.24	0.10226	YES	YES
48	a	713.01	9.39664	YES	YES
49	a	739.23	1.59901	YES	YES
50	a	779.13	10.67122	YES	YES

C-F_4

SCF Energy (au) BP86/SV(P)	-1312.120393689	
SCF Energy (au) PBE0/def2-TZVPP	-1312.044561486	
SCF Energy (au) PBE0/def2-TZVPP	-1312.0568156621	(CH_2Cl_2 Correction)
SCF Energy (au) PBE0/def2-TZVPP	-1312.0508218692	(C_6H_6 Correction)
SCF Energy (au) PBE0/def2-TZVPP	-1312.0500420769	(C_6H_{12} Correction)
Zero Point Energy (au)	0.3410881	
Chemical Potential (kJ mol^{-1})	750.83	
Dispersion Correction (au) PBE0/def2-TZVPP	-0.06456193	

xyz coordinates
46

Zr	-0.2739751	-1.3566203	-0.5580564
F	0.8998575	-1.8253110	-2.1074981
H	-1.8065084	3.1280304	3.8673614
F	-2.5495864	-0.9815699	2.1300379
N	-0.9305198	0.3740462	1.2575451
C	-1.5635957	2.3404081	3.1386739
C	-0.5610154	2.5394283	2.1870828
F	0.0586066	3.7349110	2.2082244
C	-1.8858266	0.1844721	2.1563724
C	-2.2495789	1.1250164	3.1318105
F	-3.2255690	0.8586535	4.0093318
C	0.8299991	1.6683671	0.1978521
C	-0.4886252	-3.1276532	1.3363211
H	-1.4846240	-3.3900554	1.7153475
C	1.7199023	2.9147935	0.1850249
H	2.0691518	3.1422855	1.2148954
H	1.1205069	3.8047000	-0.1214523
C	-0.2098773	1.5443021	1.2255151
C	0.2035432	-3.7726056	0.2575267
H	-0.1814361	-4.5978620	-0.3575125
C	0.9440368	0.6344235	-0.7089616
C	1.4876632	-3.1769025	0.1404874
H	2.2363862	-3.4166842	-0.6244737
C	-2.6841129	-0.3797381	-0.9404358
H	-3.1040999	0.3720097	-0.2620017
C	0.3770640	-2.1347373	1.8711363
H	0.1743509	-1.4970775	2.7396553
C	1.9404503	0.7539408	-1.8449098
H	1.4824732	0.3544635	-2.7761445
H	2.7713018	0.0336977	-1.6430359
C	-2.1815560	-2.3853124	-1.9609089
H	-2.1213496	-3.4602553	-2.1807528
C	2.9348600	2.7741128	-0.7429150
H	3.4140181	3.7690515	-0.8804809
H	3.6985569	2.1164502	-0.2639216
C	1.5878067	-2.1333830	1.1056550
H	2.4593540	-1.4873554	1.2758020
C	-2.8192901	-1.7894988	-0.8214994
H	-3.3590293	-2.3236343	-0.0291010
C	-1.9362120	-0.0975568	-2.1294061
H	-1.6732575	0.9000932	-2.5051981
C	2.5154658	2.1596351	-2.0794648
H	3.3722708	2.1109899	-2.7889572
H	1.7429801	2.8079062	-2.5578753
C	-1.6681625	-1.3393791	-2.7742815
H	-1.0827980	-1.4729961	-3.6924134

vibrational spectrum

# mode	symmetry	wave number cm**(-1)	IR intensity km/mol	selection rules IR	RAMAN
1		0.00	0.00000	-	-
2		0.00	0.00000	-	-
3		0.00	0.00000	-	-
4		0.00	0.00000	-	-
5		0.00	0.00000	-	-
6		0.00	0.00000	-	-
7	a	23.29	0.09071	YES	YES
8	a	34.06	0.00685	YES	YES
9	a	40.46	0.10062	YES	YES
10	a	44.18	0.40038	YES	YES
11	a	60.53	0.01952	YES	YES
12	a	79.85	0.92617	YES	YES
13	a	85.59	1.04245	YES	YES
14	a	98.16	1.08171	YES	YES
15	a	128.35	0.67350	YES	YES
16	a	138.41	0.03974	YES	YES
17	a	142.54	0.32545	YES	YES
18	a	179.06	0.70264	YES	YES
19	a	186.97	0.44739	YES	YES
20	a	191.96	1.58600	YES	YES
21	a	212.63	11.66937	YES	YES
22	a	214.39	0.52215	YES	YES
23	a	226.81	0.28068	YES	YES
24	a	229.71	1.65211	YES	YES
25	a	247.99	0.09039	YES	YES
26	a	250.21	0.39043	YES	YES
27	a	268.23	0.66457	YES	YES
28	a	271.46	2.60636	YES	YES
29	a	285.04	3.85324	YES	YES
30	a	287.34	4.71738	YES	YES
31	a	298.47	22.11341	YES	YES
32	a	317.84	5.96604	YES	YES
33	a	336.07	2.06181	YES	YES
34	a	339.75	11.75744	YES	YES
35	a	385.76	3.47639	YES	YES
36	a	412.15	0.03209	YES	YES
37	a	457.86	0.14194	YES	YES
38	a	467.05	0.78432	YES	YES
39	a	476.88	1.85025	YES	YES
40	a	501.79	2.62732	YES	YES
41	a	533.80	51.67195	YES	YES
42	a	535.14	4.59504	YES	YES
43	a	589.47	7.19411	YES	YES
44	a	591.31	15.06449	YES	YES
45	a	595.34	0.18381	YES	YES
46	a	597.33	1.58425	YES	YES
47	a	600.73	3.54997	YES	YES
48	a	649.26	2.50917	YES	YES
49	a	650.11	1.93910	YES	YES
50	a	707.03	8.09318	YES	YES

C-F_5

SCF Energy (au) BP86/SV(P) -1411.277222419
SCF Energy (au) PBE0/def2-TZVPP -1411.224747450
SCF Energy (au) PBE0/def2-TZVPP -1411.2360606990 (CH_2Cl_2 Correction)
SCF Energy (au) PBE0/def2-TZVPP -1411.2305455972 (C_6H_6 Correction)
SCF Energy (au) PBE0/def2-TZVPP -1411.2298253809(C_6H_{12} Correction)
Zero Point Energy (au) 0.3332158
Chemical Potential (kJ mol^{-1}) 727.00
Dispersion Correction (au) PBE0/def2-TZVPP -0.06479271

xyz coordinates
46

Zr	-0.2733425	-1.3589029	-0.5648616
F	0.8946406	-1.8192570	-2.1200036
F	-1.8729957	3.2847602	3.9950908
F	-2.5336646	-0.9892612	2.1339999
N	-0.9230815	0.3681790	1.2505438
C	-1.5673025	2.3344465	3.1159234
C	-0.5573186	2.5473967	2.1642725
F	0.0389107	3.7501537	2.1977076
C	-1.8763669	0.1795694	2.1508904
C	-2.2562172	1.1122044	3.1258018
F	-3.2260010	0.8609037	4.0091337
C	0.8328153	1.6691244	0.1915063
C	-0.4870337	-3.1292985	1.3313386
H	-1.4815570	-3.3924709	1.7139317
C	1.7247347	2.9136436	0.1840737
H	2.0669755	3.1415878	1.2165603
H	1.1301361	3.8044119	-0.1292964
C	-0.2089442	1.5438938	1.2163808
C	0.2003645	-3.7722884	0.2482575
H	-0.1884656	-4.5958385	-0.3667517
C	0.9458025	0.6353636	-0.7154714
C	1.4854100	-3.1786344	0.1289264
H	2.2320280	-3.4193998	-0.6377574
C	-2.6890291	-0.3910217	-0.9342156
H	-3.1073449	0.3592087	-0.2528321
C	0.3818409	-2.1393331	1.8657569
H	0.1829532	-1.5031051	2.7364528
C	1.9435761	0.7563106	-1.8497788
H	1.4824123	0.3658797	-2.7833343
H	2.7680142	0.0275558	-1.6532139
C	-2.1826381	-2.3945296	-1.9563751
H	-2.1172938	-3.4692874	-2.1754895
C	2.9457344	2.7670730	-0.7351919
H	3.4326472	3.7589367	-0.8672746
H	3.7013218	2.1031957	-0.2521493
C	1.5901341	-2.1378020	1.0967066
H	2.4633612	-1.4942234	1.2679012
C	-2.8169434	-1.8013210	-0.8137366
H	-3.3482706	-2.3379307	-0.0173754
C	-1.9492758	-0.1057794	-2.1275420
H	-1.6949825	0.8929162	-2.5062281
C	2.5310836	2.1585311	-2.0760294
H	3.3929390	2.1043314	-2.7789175
H	1.7684154	2.8146864	-2.5592782
C	-1.6791686	-1.3465477	-2.7732044
H	-1.0990137	-1.4780313	-3.6948478

vibrational spectrum

# mode	symmetry	wave number cm**(-1)	IR intensity km/mol	selection rules IR	RAMAN
1		-0.00	0.00000	-	-
2		-0.00	0.00000	-	-
3		-0.00	0.00000	-	-
4		-0.00	0.00000	-	-
5		0.00	0.00000	-	-
6		0.00	0.00000	-	-
7	a	22.13	0.26575	YES	YES
8	a	34.53	0.00342	YES	YES
9	a	40.83	0.10205	YES	YES
10	a	43.95	0.32029	YES	YES
11	a	56.98	0.00462	YES	YES
12	a	79.19	0.94810	YES	YES
13	a	84.87	0.96923	YES	YES
14	a	97.05	0.92087	YES	YES
15	a	125.22	0.34447	YES	YES
16	a	130.15	0.42214	YES	YES
17	a	142.71	0.35153	YES	YES
18	a	154.15	0.09026	YES	YES
19	a	172.98	1.01695	YES	YES
20	a	184.03	0.47059	YES	YES
21	a	193.33	1.56778	YES	YES
22	a	212.87	11.65026	YES	YES
23	a	215.10	0.45971	YES	YES
24	a	229.72	1.83951	YES	YES
25	a	231.98	0.53839	YES	YES
26	a	248.55	0.07019	YES	YES
27	a	261.32	0.54109	YES	YES
28	a	266.35	0.70057	YES	YES
29	a	270.72	1.73231	YES	YES
30	a	273.48	2.29166	YES	YES
31	a	288.31	6.16219	YES	YES
32	a	292.99	0.93849	YES	YES
33	a	309.68	13.29755	YES	YES
34	a	320.52	25.66550	YES	YES
35	a	336.72	0.84521	YES	YES
36	a	362.42	1.36146	YES	YES
37	a	372.12	3.23316	YES	YES
38	a	399.27	0.14531	YES	YES
39	a	457.62	1.21029	YES	YES
40	a	470.75	1.50115	YES	YES
41	a	472.40	1.71636	YES	YES
42	a	500.30	2.19490	YES	YES
43	a	535.43	50.61034	YES	YES
44	a	544.81	0.39050	YES	YES
45	a	573.61	0.05459	YES	YES
46	a	589.99	0.02078	YES	YES
47	a	595.47	0.85802	YES	YES
48	a	597.02	1.49615	YES	YES
49	a	600.46	2.32216	YES	YES
50	a	627.34	1.20033	YES	YES

Cyclohexene

SCF Energy (au) BP86/SV(P)	-234.4613676966
Zero Point Energy (au)	0.1417302
Chemical Potential (kJ mol^{-1})	296.61

xyz coordinates
16

C	-0.8095563	0.5212374	1.2332968
C	0.3013951	1.3465953	0.6248658
H	0.9200046	1.8005729	1.4341388
H	-0.1403426	2.2160882	0.0767200
C	-1.1616040	-0.7011053	0.7793858
C	-0.5096995	-1.3657651	-0.4117567
H	-1.2902805	-1.8351374	-1.0552712
H	0.1249667	-2.2172920	-0.0599695
C	1.1895896	0.5186312	-0.3232375
H	1.8388451	1.1910140	-0.9273486
H	1.8726786	-0.1227267	0.2819610
C	0.3380698	-0.3791734	-1.2371178
H	0.9830814	-0.9333221	-1.9550798
H	-0.3389558	0.2627158	-1.8485067
H	-1.3490482	0.9547701	2.0962249
H	-1.9691439	-1.2571028	1.2916949

vibrational spectrum

# mode	symmetry	wave number	IR intensity	selection rules IR	RAMAN
#		cm**(-1)	km/mol	IR	RAMAN
1		-0.00	0.00000	-	-
2		-0.00	0.00000	-	-
3		-0.00	0.00000	-	-
4		-0.00	0.00000	-	-
5		0.00	0.00000	-	-
6		0.00	0.00000	-	-
7	a	164.78	0.50480	YES	YES
8	a	272.03	0.05489	YES	YES
9	a	383.77	0.02006	YES	YES
10	a	444.25	1.53272	YES	YES
11	a	488.49	0.04790	YES	YES
12	a	633.46	25.13734	YES	YES
13	a	708.32	7.11031	YES	YES
14	a	805.54	0.95221	YES	YES
15	a	818.94	0.00157	YES	YES
16	a	874.40	6.42958	YES	YES
17	a	901.42	0.51784	YES	YES
18	a	905.89	3.76783	YES	YES
19	a	957.62	0.01199	YES	YES
20	a	985.69	1.11438	YES	YES
21	a	1030.30	5.55745	YES	YES
22	a	1057.78	0.01423	YES	YES
23	a	1062.07	0.04037	YES	YES
24	a	1124.86	0.47433	YES	YES
25	a	1124.93	3.55498	YES	YES
26	a	1207.51	0.39963	YES	YES
27	a	1232.64	0.52492	YES	YES
28	a	1253.34	2.23808	YES	YES
29	a	1306.27	1.00520	YES	YES
30	a	1337.07	2.01721	YES	YES
31	a	1337.84	0.40045	YES	YES
32	a	1346.41	0.74296	YES	YES
33	a	1376.69	0.22431	YES	YES
34	a	1408.03	0.00655	YES	YES

35	a	1415.35	10.83691	YES	YES
36	a	1426.77	5.48237	YES	YES
37	a	1437.75	2.71807	YES	YES
38	a	1679.45	3.17054	YES	YES
39	a	2903.78	1.72341	YES	YES
40	a	2904.14	61.79097	YES	YES
41	a	2933.09	19.61626	YES	YES
42	a	2937.01	17.68285	YES	YES
43	a	2958.18	20.52489	YES	YES
44	a	2958.30	53.79486	YES	YES
45	a	2989.27	42.37227	YES	YES
46	a	2993.70	44.08803	YES	YES
47	a	3051.86	7.92471	YES	YES
48	a	3073.36	39.62142	YES	YES

Cyclohexyne

SCF Energy (au) BP86/SV(P)	-233.1566380815
Zero Point Energy (au)	0.1178449
Chemical Potential (kJ mol^{-1})	234.22

xyz coordinates
14

C	-0.9655376	0.4312543	1.3851041
C	0.0570245	1.4008703	0.9275614
H	0.7047599	1.8202855	1.7296632
H	-0.4148513	2.2687114	0.4109686
C	-1.3179369	-0.6626521	0.9271330
C	-0.7843699	-1.4745742	-0.1913730
H	-1.5520814	-1.9056458	-0.8722985
H	-0.1760553	-2.3288782	0.1865600
C	0.9203889	0.5353415	-0.0722765
H	1.5124053	1.2227090	-0.7193835
H	1.6528702	-0.0526355	0.5268385
C	0.1094793	-0.4310024	-0.9701517
H	0.8115679	-0.9873548	-1.6329209
H	-0.5576635	0.1635707	-1.6354247

vibrational spectrum

# mode	symmetry	wave number	IR intensity	selection rules	
#		cm**(-1)	km/mol	IR	RAMAN
1		-0.00	0.00000	-	-
2		0.00	0.00000	-	-
3		0.00	0.00000	-	-
4		0.00	0.00000	-	-
5		0.00	0.00000	-	-
6		0.00	0.00000	-	-
7	a	212.95	7.27920	YES	YES
8	a	263.16	0.12011	YES	YES
9	a	291.39	0.76000	YES	YES
10	a	391.30	72.40311	YES	YES
11	a	477.38	1.68166	YES	YES
12	a	498.86	17.87423	YES	YES
13	a	779.75	1.58605	YES	YES
14	a	805.49	0.04129	YES	YES
15	a	823.47	11.42455	YES	YES
16	a	872.59	6.01721	YES	YES
17	a	903.07	0.02330	YES	YES
18	a	906.96	21.60128	YES	YES
19	a	999.61	2.84762	YES	YES
20	a	1040.77	1.54538	YES	YES
21	a	1095.81	1.43493	YES	YES
22	a	1117.64	0.74527	YES	YES
23	a	1124.87	5.08968	YES	YES
24	a	1191.93	1.18645	YES	YES
25	a	1193.84	0.10987	YES	YES
26	a	1256.76	11.51663	YES	YES
27	a	1295.45	2.55805	YES	YES
28	a	1296.19	0.71251	YES	YES
29	a	1332.40	5.08753	YES	YES
30	a	1408.00	1.95440	YES	YES
31	a	1409.40	5.33202	YES	YES
32	a	1420.70	2.46469	YES	YES
33	a	1429.83	0.24051	YES	YES
34	a	2129.12	6.66746	YES	YES
35	a	2942.24	21.23109	YES	YES
36	a	2944.18	3.27379	YES	YES
37	a	2945.66	78.40894	YES	YES

38	a	2947.27	20.43573	YES	YES
39	a	2987.43	8.32226	YES	YES
40	a	2988.98	26.46513	YES	YES
41	a	2996.99	3.37600	YES	YES
42	a	3006.49	47.76601	YES	YES

4 References

1 A. M. Borys, *Organometallics*, **2023**, *42*, 182.

2 R. K Harris, E. D. Becker, S. M. Cabral De Menezes, R. Goodfellow and P. Granger, *Pure Appl. Chem.*, **2001**, *73*, 1795.

3 R. K. Harris, E. D. Becker, S. M. C. de Menezes, P. Granger, R. E. Hoffman and K. W. Zilm, *Pure Appl. Chem.*, **2008**, *80*, 59.

4 A. Spaggiari, D. Vaccari, P. Davoli, G. Torre and F. Prati, *J. Org. Chem.*, **2007**, *72*, 2216.

5 K. Garrec and S. P. Fletcher, *Org. Lett*., **2016**, *18*, 3814.

6 S. L. Buchwald, R. Y. Lum and J. C. Dewan, *J. Am. Chem. Soc.*, **1986**, *108*, 7441.

7 J. Skotnitzki, A. Kremsmair, D. Keefer, Y. Gong, R. de Vivie-Riedle and P. Knochel, *Angew. Chem. Int. Ed.*, **2020**, *59*, 320.

8 J. Schwabedissen, P. C. Trapp, H. G. Stammler, B. Neumann, J. H. Lamm, Y. V. Vishnevskiy, L. A. Körte and N. W. Mitzel, *Chem. Eur. J.*, **2019**, *25*, 7339.

9 H. Bauer, M. Alonso, C. Fischer, B. Rösch, H. Elsen and S. Harder, *Angew. Chem. Int. Ed.*, **2018**, *57*, 15177.

10 K. M. Doxsee, E. M. Hanawalt and T. J. R. Weakley, *Inorg. Chem*., **2002**, *31*, 4420.

11 M. Wiesemann, H.-G. Stammler, B. Neumann and B. Hoge, *Eur. J. Inorg. Chem.*, **2017**, 4733.

12 P. Császár and P. Pulay, *J. Mol. Struct.*, **1984**, *114*, 31.

13 R. Ahlrichs, M. Bär, M. Häser, H. Horn and C. Kölmel, *Chem. Phys. Lett.*, **1989**, *162*, 165.

14 P. Deglmann, F. Furche and R. Ahlrichs, *Chem. Phys. Lett.*, **2002**, *362*, 511.

15 P. Deglmann, K. May, F. Furche and R. Ahlrichs, *Chem. Phys. Lett.*, **2004**, *384*, 103.

16 K. Eichkorn, O. Treutler, H. Öhm, M. Häser and R. Ahlrichs, *Chem. Phys. Lett.*, **1995**, *242*, 652.

17 K. Eichkorn, F. Weigend, O. Treutler and R. Ahlrichs, *Theor. Chem. Acc.*, **1997**, *97*, 119.

18 O. Treutler and R. Ahlrichs, *J. Chem. Phys.*, **1995**, *102*, 346.

19 M. von Arnim and R. Ahlrichs, *J. Chem. Phys.*, **1999**, *111*, 9183.

20 A. Klamt and G. Schuurmann, *J. Chem. Soc., Perkin Trans.* 2, **1993**, 799.

21 S. Grimme, J. Antony, S. Ehrlich and Helge Krieg,*J. Chem. Phys.*, **2010**, *132*, 154104.

22 S. Grimme, S. Ehrlich and L. Goerigk, *J. Comput. Chem.,* **2011**, *32*, 1456.
